# Cell-type-specific effects of autism-associated 15q duplication syndrome in the human brain

**DOI:** 10.1016/j.ajhg.2024.07.002

**Published:** 2024-07-29

**Authors:** Caroline Dias, Alisa Mo, Chunhui Cai, Liang Sun, Kristen Cabral, Catherine A. Brownstein, Shira Rockowitz, Christopher A. Walsh

**Affiliations:** 1 Division of Developmental Medicine, Boston Children’s Hospital, Boston, MA 02115, USA; 2 Division of Genetics and Genomics, Manton Center for Orphan Disease Research, Boston Children’s Hospital, Boston, MA 02115, USA; 3 Department of Pediatrics, Harvard Medical School, Boston, MA 02115, USA; 4 Department of Neurology, Boston Children’s Hospital, Harvard Medical School, Boston, MA 02115, USA; 5 Research Computing, Department of Information Technology, Boston Children’s Hospital, Boston, MA 02115, USA; 6 Howard Hughes Medical Institute, Boston Children’s Hospital, Boston, MA 02115, USA

**Keywords:** dup15q, autism, snRNA-seq, ATAC-seq, copy-number variant, neurodevelopment

## Abstract

Recurrent copy-number variation represents one of the most well-established genetic drivers in neurodevelopmental disorders, including autism spectrum disorder. Duplication of 15q11–q13 (dup15q) is a well-described neurodevelopmental syndrome that increases the risk of autism more than 40-fold. However, the effects of this duplication on gene expression and chromatin accessibility in specific cell types in the human brain remain unknown. To identify the cell-type-specific transcriptional and epigenetic effects of dup15q in the human frontal cortex, we conducted single-nucleus RNA sequencing and multi-omic sequencing on dup15q-affected individuals (*n* = 6) as well as individuals with non-dup15q autism (*n* = 7) and neurotypical control individuals (*n* = 7). Cell-type-specific differential expression analysis identified significantly regulated genes, critical biological pathways, and differentially accessible genomic regions. Although there was overall increased gene expression across the duplicated genomic region, cellular identity represented an important factor mediating gene-expression changes. As compared to other cell types, neuronal subtypes showed greater upregulation of gene expression across a critical region within the duplication. Genes that fell within the duplicated region and had high baseline expression in control individuals showed only modest changes in dup15q, regardless of cell type. Of note, dup15q and autism had largely distinct signatures of chromatin accessibility but shared the majority of transcriptional regulatory motifs, suggesting convergent biological pathways. However, the transcriptional binding-factor motifs implicated in each condition implicated distinct biological mechanisms: neuronal JUN and FOS networks in autism vs. an inflammatory transcriptional network in dup15q microglia. This work provides a cell-type-specific analysis of how dup15q changes gene expression and chromatin accessibility in the human brain, and it finds evidence of marked cell-type-specific effects of this genetic driver. These findings have implications for guiding therapeutic development in dup15q syndrome, as well as understanding the functional effects of copy-number variants more broadly in neurodevelopmental disorders.

## Teaser

Recurrent copy-number variation represents a well-established genetic driver in neurodevelopmental disorders. We applied single-nucleus approaches to postmortem brain tissue from individuals with autism-associated dup15q (*n* = 6), autism (*n* = 7), and neurotypical controls (*n* = 7). We found unexpected cell-type-specific gene expression and chromatin accessibility changes that will guide future research and therapeutic development.

## Introduction

**Figure 1:**
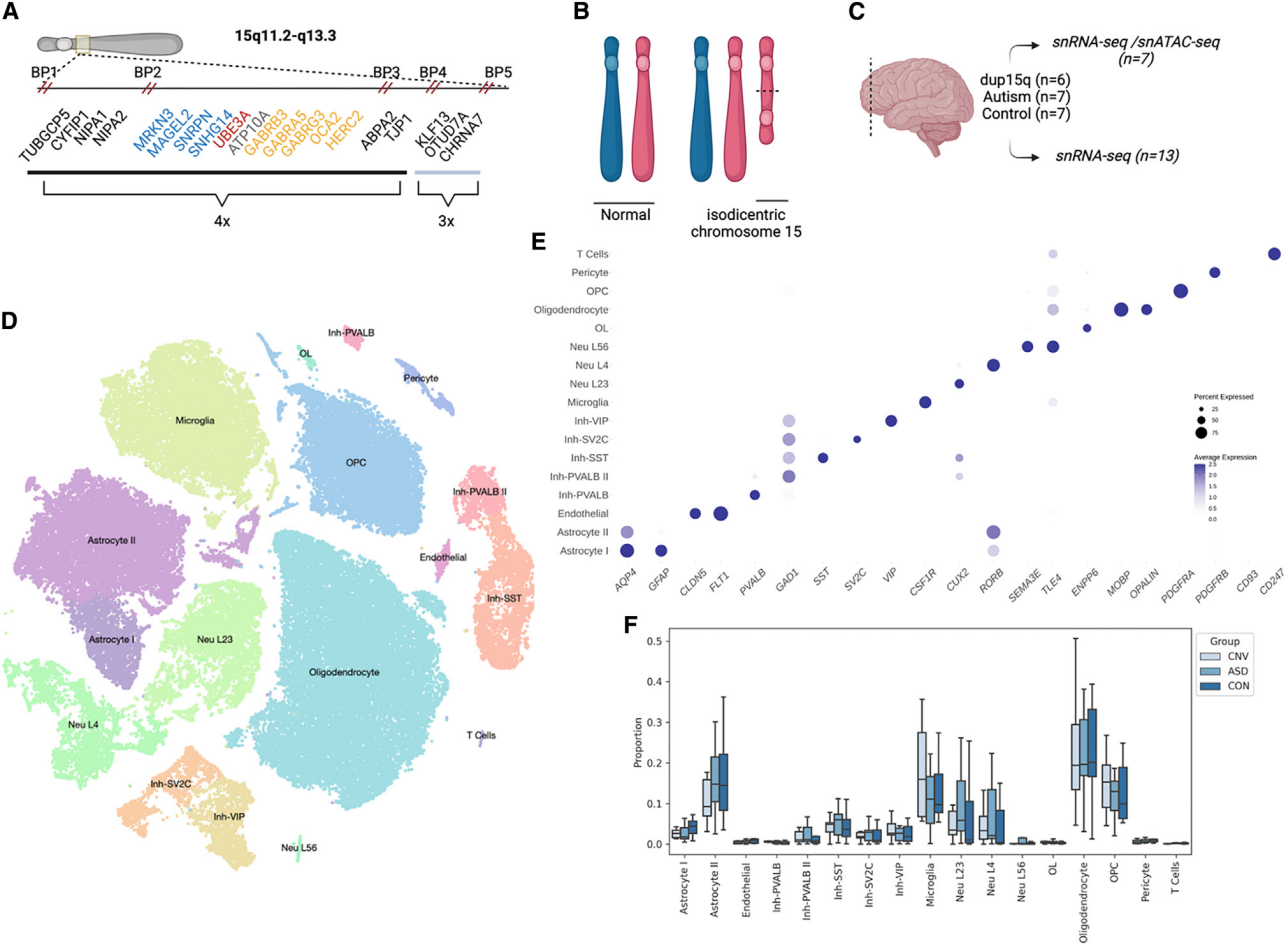
Single-nucleus analysis of dup15q in the human brain (A) Genomic architecture of chromosome 15 with only select genes shown for clarity. Inset shows the location of genes, with breakpoints and copy-number indicated. Critical imprinting locus between breakpoints 2 and 3 (BP2 and BP3). Blue: paternally imprinted. Red: maternally imprinted. Yellow: bi-allelic. Gray: conflicting reports. (B) Cartoon representation of dup15q structure due to isodicentric maternal chromosome 15. (C) Samples used from frontal cortex. (D) Visualization of single-nucleus data with t-SNE dimension reduction. (E) Cell-type-specific gene expression indicates representation of expected cell types in human frontal cortex and accurate clustering. (F) Cell-type compositional analysis indicates no significant changes in proportion of cell types between conditions. Boxplot shows median, interquartile range (box), and ±1.5 interquartile range (error bars) of the proportion of each cell type.

The proximal end of the long arm of chromosome 15 is a genomic region containing several segmental duplications, making it particularly susceptible to complex rearrangements at recurrent breakpoints (Figure 1A).^1^ Parental chromosome-specific deletions in this region lead to the imprinting disorders Angelman syndrome (MIM: 105830) and Prader-Willi syndrome (MIM: 176270), whereas maternal duplication of 15q11–q13, referred to hereafter as dup15q syndrome (MIM: 608636), is associated with autism spectrum disorder (ASD), intellectual disability, hypotonia, and epilepsy.^2^ There is over a 40-fold increased risk of ASD in individuals carrying this duplication, making it one of the most significant and highly penetrant genetic drivers of ASD.^3^^,^^4^

Most cases of dup15q are caused by an isodicentric chromosome 15 (Figure 1B),^5^ and many of these are in turn driven by an asymmetric recombination event between breakpoints 4 and 5. This leads to tetrasomy from the centromere to breakpoint 4 (a region that includes the Prader-Willi/Angelman critical region [PWACR] between breakpoints 2 and 3) and trisomy from breakpoint 4 to 5 (Figure 1A).^2^ Given that most cases are caused by maternally derived duplications and that the duplication contains a critical imprinting locus within the PWACR, copy-number alterations in maternally imprinted, dosage-sensitive genes such as *UBE3A* (MIM: 601623) have been hypothesized to underlie the dup15q clinical phenotype. However, the dup15q region also contains a GABA receptor cluster, and some individuals with dup15q have atypical responses to benzodiazepines, potentially implicating these and other genes as well.

The cell-type-specific effects of dup15q on gene expression in the human brain are unknown. Prior work in postmortem brains has shown that in bulk tissue, *UBE3A* expression does not match changes in gene dosage in dup15q-affected individuals.^6^ However, bulk brain tissue analysis cannot identify whether only specific cell types showed changes in gene expression. Understanding how cell-type-specific heterogeneity in gene expression and chromatin accessibility contributes to pathophysiology is the first step in identifying potential therapeutic targets and advancing precision medicine for this disorder.

It is also critical to understand the similarities and differences on the molecular and cellular level between dup15q and ASD not related to dup15q. Although prior studies have demonstrated similarities on the molecular level in the brain between individuals with dup15q and ASD, suggesting convergent biological processes,^7^^,^^8^^,^^9^ there are also important clinical distinctions in the developmental and behavioral profile of these subgroups. Individuals with dup15q syndrome might have an initially preserved social smile and yet more marked neurological co-morbidities, including greater motor impairment.^10^^,^^11^ The complexity of this genomic region, in addition with the long-standing challenge of studying the heterogeneity of brain tissue, has made it difficult to directly parse the effects of this variant in the human brain. In this study we sought to examine the transcriptional and epigenetic landscape of dup15q brain cell types compared to those of both neurotypical control individuals and individuals with non-dup15q ASD; we hypothesized that cell-type-specific effects, previously masked by brain-tissue heterogeneity, would reveal important biological distinctions in cellular pathophysiology and provide foundational information for future mechanistic and translational studies.

## Material and methods

### Brain tissue

Tissue was obtained from the NIH NeuroBioBank and Autism BrainNet with approval from their institutional review boards after written informed consent was obtained. Initial dissection of tissue for brain-bank specimens was done with sequential sectioning under standardized procedures. Research on these deidentified specimens and data was performed at Boston Children’s Hospital with approval from the Committee on Clinical Investigation. Postmortem frozen frontal cortex was obtained from six affected individuals with dup15q, seven individuals with non-dup15q autism spectrum disorder (ASD), and seven neurotypical control individuals. 13 samples were processed for single-nucleus RNA sequencing (snRNA-seq), and seven for multi-omic sequencing (Figure 1C). All of the dup15q affected individuals were validated through copy-number analysis of the PWACR (tetrasomy)^6^^,^^7^ and/or optical genome mapping (Figure S1). Methylation status in the brain has also been previously assessed in the majority of dup15q-affected individuals.^6^^,^^12^ Groups were matched for age and sex (14%–17% female), and there were no significant differences between the RNA integrity number (RIN) and postmortem interval (PMI) between groups (Table 1; Figure S2). Frozen tissue was stored at −80°C and kept frozen until processing. ∼25 mg pieces of tissue were dissected, with pre-chilled sterile forceps and scalpels, off the larger tissue block on a pre-chilled cryostat maintained at −20°C.

**Table 1:** Demographic information for samples

**ID**	**Condition**	**Age**	**Sex**	**PMI**	**RIN**	**Seizure history**
797	ASD	9	M	13	7.7	–
UMB1790	control	13	M	18	8.2	–
1793	control	11	M	19	–	–
UMB4643	control	42	F	4	8.2	–
5023	ASD	45	M	–	–	–
5278	ASD	15	F	13	6.9	yes
5391	control	8	M	19	6.9	–
5408	control	6	M	12	–	–
5497	control	68	M	24	8	–
7TJ5	ASD	13	M	26	6.7	–
862K	dup15q	12	M	30	5.4	yes
AN05983	dup15q	24	M	36	7.6	yes
AN06365	dup15q	10	M	18	8.1	yes
AN09412	ASD	29	M	38	7.3	–
AN11931	dup15q	39	F	33	8.5	yes
AN14067	ASD	38	M	46	8.7	yes
AN14762	dup15q	9	M	14	–	yes
PGEQ	control	16	M	23	6.8	–
UMB5565	ASD	12	M	22	–	–
Z2WU	dup15q	13	M	17	7.4	yes

PMI, postmortem interval. RIN, RNA integrity number.

### snRNA-sequencing and multi-omic sequencing

For nuclear isolation, tissue was subjected to glass Dounce homogenization followed by sucrose gradient centrifugation to isolate nuclei, as previously described.^13^ Results presented here are from one technical replicate of each sample, but samples were run in subsets of matched groups over multiple days and sequencing runs so that batch effects would be minimized. For snRNA-seq, nuclei were resuspended in a nuclear resuspension buffer (1% BSA in PBS with 1 U/uL RNase inhibitor) and stained with Hoechst immediately prior to fluorescent-activated nuclear sorting on a BD Aria II, during which gating was used to remove debris, dying nuclei, and doublets. 10,000 nuclei/sample were sorted with a 100 μm low-pressure nozzle and immediately processed for 10× encapsulation as described below so that single-nucleus libraries could be generated. After encapsulation on the 10× Chromium controller, libraries were prepared as per the 10× Genomics Single Cell 3′ Kit. cDNA and library quality control were ensured through assessment of DNA on the Agilent Bioanalyzer High Sensitivity Chip.

For generation of multiome libraries, 50,000 nuclei were sorted as above directly into buffer (10 mM Tris-HCl [pH 7.4], 10 mM NaCl, 3 mM MgCl_2_, 1% BSA, 1 mM DTT, Rnase inhibitor 1 U/uL). 1× Lysis Buffer (10 mM Tris-HCl, 10 mM NaCl, 3 mM MgCl_2_, 0.1% Tween 20, 0.1% NP-40, 0.01% Digitonin, 1% BSA, 1 mM DTT, 1 U/uL Rnase inhibitor) was added to a final concentration of 0.1×, and the nuclei were incubated on ice for 2 min. Tween 20 was added (final concentration 0.1%), and nuclei were immediately pelleted by centrifugation at 500*g* × 5 min at 4°C in a bucket centrifuge. The pellet was resuspended in Diluted Nuclei Buffer (1× Nuclei Buffer (10× Genomics), 1 mM DTT, 1 U/uL RNase inhibitor) and centrifuged. The number of nuclei in the pellet was quantified with a hemocytometer before use of the 10× Genomics Chromium Next GEM Single Cell Multiome ATAC + Gene Expression kit according to the manufacturer’s instructions.

Libraries were sequenced with paired-end 150 bp reads on an Illumina NovaSeq 6000.

### Bioinformatics analysis

For snRNA-seq analysis, the following steps were taken to process the samples: demultiplexing of raw data, alignment (to GRCh382020-A), quality control and filtering (see below), dimensionality reduction and unsupervised clustering. R (v.4.2.3), Cell Ranger (v.6.1.1.), and Seurat (v.4.3.0)^14^ were used on the Boston Children’s computing cluster E2. SoupX^15^ was applied so that ambient RNAs would be removed, and scds^16^ was used so that doublets and cells with extreme library sizes (out of the 95% confidence interval), an extreme number of features, and high content of mitochondrial reads (>10%) would be filtered out. We do not omit any sample based on the RIN given past work suggesting the RIN itself may be of more limited utility in the postmortem brain.^17^^,^^18^^,^^19^^,^^20^ Quality control was performed over each independent sample, and ∼50%–80% of nuclei remained after bioinformatic filtering, depending on the sample; there were equivalent metrics between conditions (Figure S3). Post-hoc analysis of known cell-type-specific and layer-specific markers were applied to the remaining nuclei for cluster identification and resolution optimization. One cluster representing a small percentage of nuclei (<1%) was manually removed given signatures of ambient RNA contamination as previously reported.^21^ There is increased variability in snRNA-seq studies from human tissue. Thus, in addition to ensuring that there were no significant differences between age, sex, RIN, or PMI (Table 1; Figure S2), we also assessed the impact of various demographic factors on the variability of gene expression. We found minimal impact of age and sex via principal component analysis (Figure S2) and thus conducted differential-expression analysis with the FindMarkers functionality in Seurat by using default settings for the Wilcoxon rank-sum test, minimum log fold change > 0.2, and padj < 0.01 for all cell clusters. Our power calculations suggest that approximately 400 nuclei/condition are needed to detect 80% of gene expression changes with a false-discovery rate of 5%. We omitted downsampling to preserve power. For gene ontology (GO) enrichment, we used clusterProfiler R package.^22^^,^^23^ A pathway is treated as enriched if an adjusted *p* values (with Benjamin-Hochberg correction) were smaller than 0.05. To identify changes in cellular composition, we used scCODA (single-cell compositional data analysis), a Bayesian hierarchical model developed for analyzing compositional data from single-cell RNA-sequencing studies.^24^ It identifies cell types that are differentially abundant between conditions while accounting for the compositional nature of single-cell data.

We employed inferCNV (v.1.14.2),^25^ a computational tool designed to infer copy-number variation (CNV) from single-cell RNA sequencing data, to investigate genomic instability across various conditions and cell types by using default parameters. We also utilized the R packages NGCHM (v.0.13.0) and infercnvNGCHM (v.0.1.1) for generating NGCHM files, thereby enhancing our inferCNV data interpretation and visualization with the NGCHM (Next-Generation Clustered HeatMap) interactive viewer.^26^

For multiome analysis, we mapped each sample to the human reference genome (CRCh38-2020-A-2.0.0) by using CellRanger Arc (v.2.0.0) with stringent snATAC cell filtering criteria, including counts per cell ranging from 1,000 to 1,000,000, nucleosome signal less than 2, and transcription start site (TSS) enrichment score larger than 1. Data preprocessing and normalization were conducted with Signac (v.1.9.0),^27^ and sample integration was achieved through Harmony (v.0.1.1).^28^ Cell-type annotations were informed by snRNAseq analysis results. We conducted motif enrichment analysis by using Signac; we added motif information to the Seurat object via the AddMotifs function and identified overrepresented motifs between various conditions by using FindMarkers. We computed motif activities per cell by using chromVAR (v.1.5.0).^29^ For visualization, we generated multiple genomic plot types through Signac; we included accessibility tracks and gene annotations and leveraged the CoveragePlot() function for comprehensive genome-browser-style presentations. This integrative approach allowed for a nuanced exploration of genomic landscapes, highlighting differential accessibility and gene expression in various cell types.

To orthogonally assess the overlap between gene expression and ATAC-seq data, we implemented SCENIC (*s*ingle-*c*ell r*e*gulatory *n*etwork *i*nference and *c*lustering)^30^ through its fast, python-based implementation (pySCENIC). Note that we could not directly compare the Signac and Seurat output with that of SCENIC because of differences in motif assignment. Thus, the associated transcription factors for all analyses were related and compared. We identified a statistically significant overlap in the dup15q vs. control comparison: our approach identified 382 predicted regulatory transcription factors, SCENIC identified 64, and there was an overlap of 22 (hypergeometic test, *p* < 0.01).

To generate a list of high-interest genes related to the duplicated region used for visualization and to focus our analysis, we utilized the GRCh38/hg38 assembly accessed through genome.ucsc.edu in conjunction with prior publications^1^^,^^6^^,^^7^^,^^31^; given individual differences in precise breakpoints, we also included genes beyond breakpoint 5 for comparative purposes.

### Optical mapping

A custom protocol for human brain tissue was utilized (Bionano Genomics DN30400, Rev A). See supplemental methods for detailed protocol.

## Results

After bioinformatic filtering, ∼78,815 high-quality nuclei remained for the combined analysis (Figure 1D). Although there was individual variability in cell-type breakdown, each condition had comparable numbers of nuclei (Tables 2 and 3). Post-hoc annotation of cluster types from unsupervised clustering confirmed the expected distribution of layer-specific excitatory neurons, inhibitory neuron subtypes, glia, and other non-neuronal subtypes that have been previously described in cortical human tissue (Figure 1E).^13^^,^^32^^,^^33^^,^^34^ No statistically significant changes in the composition of cell types between conditions was observed (Table 2; Figure 1F), consistent with independent reports.^35^

**Table 2:** Nuclei number per condition and totals

**Cell type**	**dup15q/CNV**	**ASD**	**Control**	**Total**
*Astrocyte I*	624	627	1,257	2,508
*Astrocyte II*	3,214	3,973	4,461	11,648
Endothelial	107	139	239	485
Inh-PVALB	158	103	113	374
Inh-PVALB II	431	416	473	1,320
Inh-SST	1,034	969	1,324	3,327
Inh-SV2C	699	454	683	1,836
Inh-VIP	871	475	776	2,122
*Microglia*	4,228	2,929	3,806	10,963
Neu L23	1,869	1,722	2,623	6,214
Neu L4	1,240	1,250	1,733	4,223
Neu L56	32	149	70	251
*OL*	110	120	84	314
*Oligodendrocyte*	5,409	5,869	10,888	22,166
*OPC*	3,662	2,916	3,650	10,228
Pericyte	165	196	313	674
T-cells	33	51	78	162
Total	23,886	22,358	32,571	78,815

Italics indicate cell types that are subsequently grouped in “glia” category, and all inhibitory (Inh) and excitatory (Neu) neurons are grouped as “neuron,” for “low-resolution” analysis.

**Table 3:** Number of nuclei per sample

	**Astrocyte I**	**Astrocyte II**	**Endothelial**	**Inh-PVALB**	**Inh-PVALB II**	**Inh-SST**	**Inh-SV2C**	**Inh-VIP**	**Microglia**	**Neu L23**	**Neu L4**	**Neu L56**	**OL**	**Oligodendrocyte**	**OPC**	**Pericyte**	**T cell**
AN14762	114	659	4	16	19	89	54	84	901	124	172	2	24	460	998	5	2
AN06365	44	98	2	0	0	0	0	1	909	0	1	0	13	1614	489	2	16
AN05983	178	1410	35	23	32	198	86	126	268	151	40	4	12	927	631	36	6
AN11931	111	331	2	54	133	250	320	261	180	1001	240	4	0	149	145	6	0
ABN_862K	61	288	35	24	57	217	76	101	1,565	90	91	3	30	1395	309	42	6
ABN_Z2WU	116	428	29	41	190	280	163	298	405	503	696	19	31	864	1,090	74	3
797	64	988	24	12	50	161	55	70	1,004	265	96	7	17	784	842	74	5
5278	184	980	36	1	9	13	27	4	862	35	41	0	40	1778	602	30	14
AN09412	17	58	9	26	63	105	64	37	1	245	209	45	2	29	19	4	3
AN14067	169	392	3	0	2	26	0	6	390	5	2	0	3	1,377	272	6	8
5023	138	1,006	1	19	36	254	98	131	159	430	64	2	3	772	211	12	6
ABN_7TJ5	15	76	40	27	149	183	113	82	337	555	388	48	13	498	461	41	11
UMB5565	40	473	26	18	107	227	97	145	176	187	450	47	42	631	509	29	4
5408	228	789	42	0	1	9	6	6	1,029	0	1	0	21	613	935	40	36
5391	99	676	18	27	118	323	116	111	581	1,518	899	26	7	1,012	412	18	10
1793	136	938	3	2	20	91	25	79	557	27	10	0	8	33	636	22	2
5497	308	988	58	6	23	157	39	56	549	28	9	0	0	1,672	241	111	2
UMB1790	44	188	70	54	245	359	322	299	290	908	681	36	16	1,082	706	61	0
ABN_PGEQ	125	495	32	24	66	378	171	220	288	140	131	8	24	920	340	43	17
UMB4643	317	387	16	0	0	7	4	5	512	2	2	0	8	5,556	380	18	11
Total	2508	11,648	485	374	1,320	3,327	1,836	2,122	10,963	6,214	4,223	251	314	22,166	10,228	674	162

**Figure 2:**
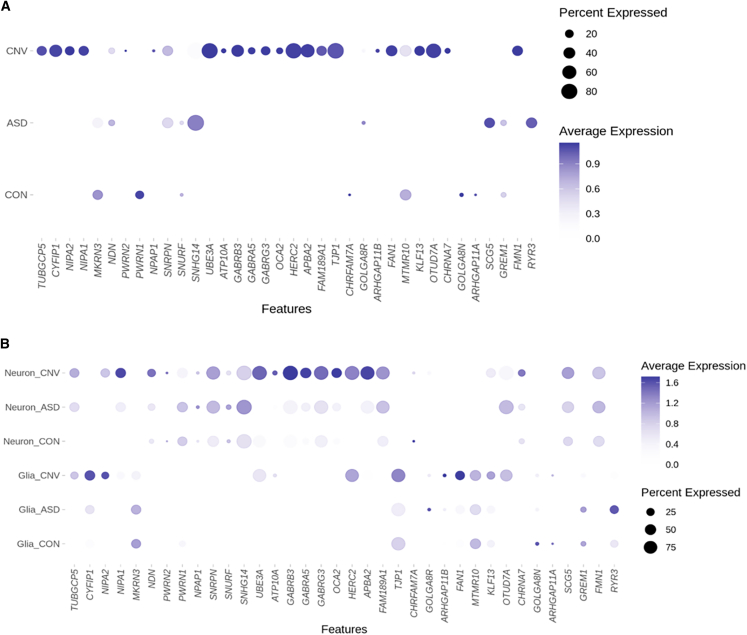
Expression changes within the proximal region of the long arm of chromosome 15 in ASD, dup15q (CNV), and control (CON) individuals (A) Expected upregulation of the duplicated region when all nuclei are examined. (B) Neuronal and glial nuclei broken down by condition demonstrate distinct patterns of changes in gene expression within this region; neurons demonstrate marked upregulation of the PWACR. The color heatmap is scaled average expression. Note that because visualization includes all cell types and scaled average expression is used, basal and ceiling effects might mask visualization of some changes between conditions. The list of significant differentially regulated genes can be found in Data S1.

Using all nuclei in a pseudo-bulk tissue analysis demonstrated the expected upregulation of gene expression within the duplicated region, including *UBE3A*, in dup15q tissue compared to control tissue, consistent with prior published bulk RNA-seq work^7^ (Figure 2A, Data S1; Table S1). We also identified more DEGs globally in dup15q vs. control tissue, as compared to ASD vs. control or dup15q vs. ASD, suggesting unique biological perturbations in dup15q (Table S1).

We then conducted both high- and low-resolution expression analyses. First, for a “low-resolution” approach, we grouped broad neuronal subtypes (inhibitory and excitatory) and glial subtypes (oligodendrocyte lineage, microglia, and astrocytes). This approach maximized our power to detect expression changes within these categories.^36^ Genome-wide, dup15q vs. control comparisons demonstrated the largest number of differentially expressed genes, in both neurons and glia, consistent with the broad analysis described above (Table S1). Compared to glia, neurons showed more marked increased expression across the duplicated region (Figure 2B). Genes within the PWACR region demonstrated preferential upregulation in neurons vs. glia in dup15q cases. (Data S1, Figure 2B). Not all genes demonstrated upregulation in both neurons and glia, although some, such as *UBE3A* and *HERC2*, did.

**Figure 3:**
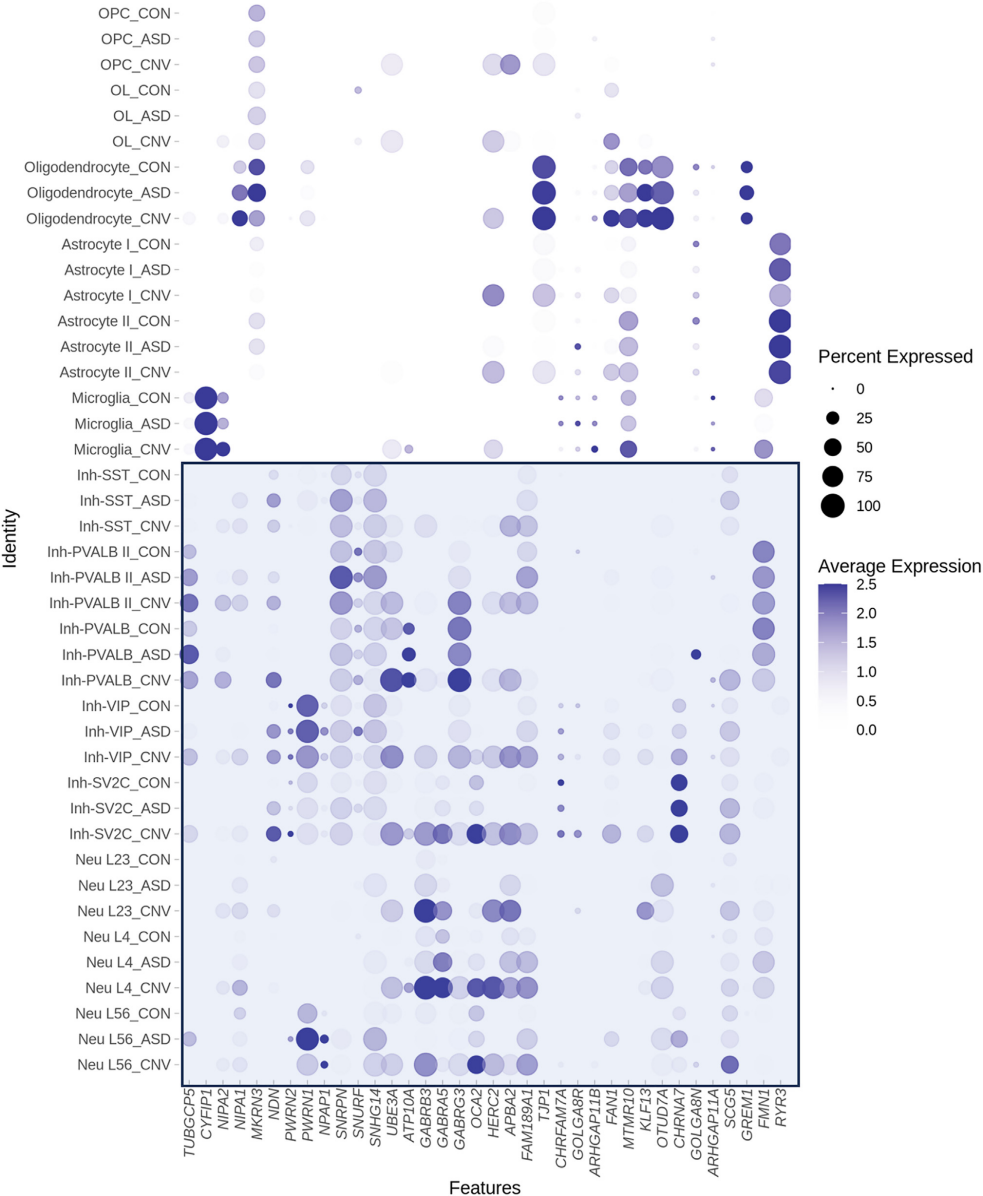
Dot plot depicting global cell-type-specific expression within the proximal region of chromosomal region 15q The blue box highlights neuronal (bottom) subtypes. Note that because visualization includes all cell types, basal and ceiling effects might mask visualization of some changes between conditions. The list of significant differentially regulated genes can be found in Data S1.

We also conducted “high-resolution” analysis that assessed changes in gene expression in more finely resolved individual cell types (e.g., layer 2/3 excitatory neurons and microglia). This analysis reinforced our findings above—namely, that cellular context is a critical parameter in gene expression changes, and cell-type-specific effects are observed even within neuron or glial subclasses (Data S1, Figure 3; Table S2). For example, the cluster of GABA receptor genes located within the PWACR demonstrated heterogeneity in gene expression changes between individual neuronal subtypes (Figure 3, Data S1). GABRB3 was upregulated in OPCs, and all inhibitory and excitatory neuron subtypes except for neurons in layers 5 and 6. GABRA5, on the other hand, was upregulated only in SV2C inhibitory neurons and excitatory neurons in layers 2–4. Thus, there was unexpected heterogeneity even across similar genes and cell types (Figure 3).

We took an orthogonal bioinformatic approach to confirm these cell-type-specific changes in gene expression in dup15q-affected individuals across the duplication. Bioinformatic tools, such as inferCNV, have been developed to infer somatic copy-number-variant status from single-cell transcriptomic studies in the cancer biology field, in which structural genomic alterations can confer cellular survival advantages and underlie clonal expansion.^25^ We reasoned that although dup15q is a germline event, applying such tools to our data would reveal underlying heterogeneity in gene expression across the duplication. InferCNV applies a hidden Markov model to predict six states of gene dosage ranging from complete loss (state 1) to three or more copies (state 6) on the basis of single-cell RNA sequencing. As expected, applying inferCNV to all dup15q nuclei resulted in the prediction of a higher proportion of high-gene-dosage states in dup15q nuclei as compared to ASD nuclei (chi-square *p* < 0 .001). However, as expected, upregulation across the region of duplication was heterogeneous (Figure S5A). This again suggests that there is marked individual variability in dup15q changes and furthermore that not all cell types demonstrate “expected” changes within the duplicated region in dup15q in the human brain.

We next sought to identify potential mediating factors of gene expression changes. Differential gene expression studies typically focus on cells in which genes of interest are highly expressed at baseline, given the logic that those would be the cell types critical to understanding cellular pathophysiology; but copy-number gains create a situation where cell types with normally low expression may demonstrate greater fold changes in gene expression. We observed such a pattern with *CYFIP1* (MIM# 606322), located within the duplicated region. *CYFIP1* is normally highly expressed in microglia, and prior work has examined the role of microglial CYFIP1 in neurodevelopment.^37^^,^^38^ In all nuclei taken together, *CYFIP1* mRNA was identified as significantly upregulated in dup15q (Data S1); this upregulation in expression was associated with increases in chromatin accessibility (Figure S4). However, these global changes were not observed in microglia, likely due to its high basal expression (Figure S4). Rather, cell types that demonstrated significant upregulation of *CYFIP1* were all neuronal, including inhibitory VIP and PVALB I and II neurons, and excitatory neurons in layers 2–4 (Data S1). Thus, our approach reveals that genes previously implicated in neurodevelopment can become mis-expressed in non-canonical cell types in dup15q.

**Figure 4:**
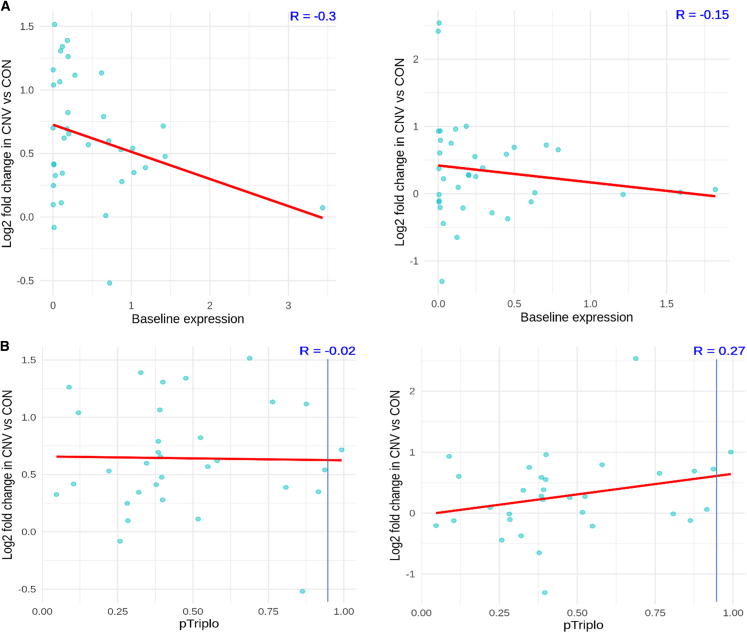
dup15q expression changes are not explained by baseline expression or genome-wide metrics (A) Relationship of baseline expression of dup15q genes and relative change in neurons (left) vs. glia (right). Regardless of cell type, genes with high baseline expression show modest increases in dup15q-affected individuals. (B) Relationship of pTriplo metric and relative change in dup15q genes in neurons (left) and glia (right). pTriplo > 0.94 is categorized as “triplosensitive.” Note the dearth of dup15q genes meeting this criteria.

To determine whether baseline expression could be mediating cell-type-specific effects on gene expression within the duplicated region, we examined the relationship between baseline expression in control nuclei and the change in expression in dup15q nuclei. There was no shift in distribution in the baseline expression of genes of interest in the duplicated region in control nuclei in neurons versus glia. In dup15q nuclei, genes with the highest baseline expression demonstrated a modest change, a finding robust among different cell types. (Figure 4A) Thus, the neuronal-specific signature of increased expression was not due to neurons' having different baseline expression of the genes within this region.

Given that not all genes within a CNV are necessarily critical to the phenotype, we sought to clarify which genes would be both dysregulated and critical to the dup15q phenotype. To do this, we made use of recent advances in quantifying genome-wide dosage sensitivity to overexpression. Specifically, the “pTriplo” metric, or the probability of triplosensitivity, was recently developed from a cross-disorder catalog of rare CNVs that confer susceptibility to human disease; a number closer to one indicates a higher likelihood of being triplosensitive.^39^ We hypothesized that many genes within the locus, particularly within the PWACR, previously deemed critical for the clinical phenotype, would demonstrate a high triplosensitive score and that a subset of those furthermore would show the highest relative change in expression in dup15q-affected individuals. Surprisingly, most genes of interest within the locus did not meet criteria for being triplosensitive as defined by the cutoff score of >0.94. (Figure S5). Furthermore, genes with a higher triplosensitivity metric again showed modest changes in gene expression, suggestive that homeostatic regulatory mechanisms are at play, regardless of cell type examined.

Carriers of dup15q have higher rates of epilepsy,^5^^,^^40^ and in fact all of the dup15q-affected individuals analyzed here have a history of this co-morbidity. To determine whether this might be influencing changes in gene expression within the region of the duplication, we separated out the (non-dup15q) ASD cases by the presence or absence of seizure diagnosis. We observed no global difference between the presence of an epilepsy or seizure diagnosis and its absence in non-dup15 ASD within our region of interest, and neither of these sub-groups resembled dup15q-affected individuals, arguing against the presence of seizures as a major confounding factor (Figure S6). We did observe notable heterogeneity from sample to sample, but no individual sample drove these findings, again suggesting that our results are robust and not due to individual sample differences in either demographic or technical factors (Figure S6; Table S3).

Our dataset also affords an opportunity to compare the analyses presented here to independent cell-type-specific reports on ASD. Encouragingly, we replicated past findings of neurodevelopmental disorders in our own gene expression data; these findings included enrichment of known pathogenic ASD risk genes, FMR1 protein target genes, and dysregulation of layer 2/3 excitatory neurons (Figures S7A and S7B).^7^^,^^9^^,^^32^^,^^35^ Using gene ontology analysis, we also found evidence for biological processes involved in synaptic function in both excitatory and inhibitory subtypes in ASD and dup15q (Figure S7C).

**Figure 5:**
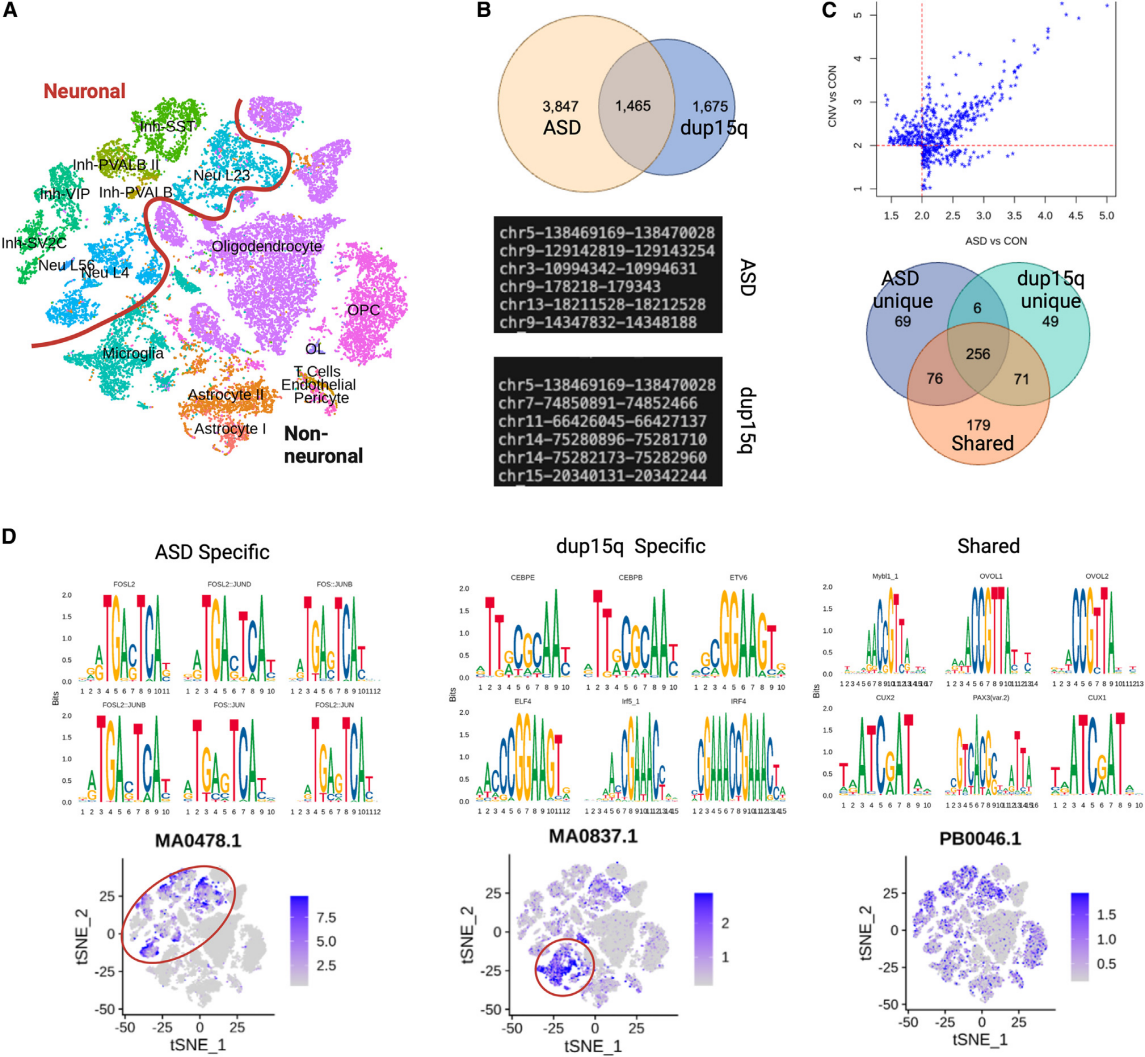
Multi-omic analysis reveals cell-type-specific transcriptional regulatory activity in dup15q-affected individuals (A) Visualization of integration of snRNA-seq and snATAC-seq. (B) Differential binding analysis revealed twice as many unique peaks in ASD vs. control nuclei as compared to dup15q nuclei vs. control. Examples of genomic locations for ASD and dup15q shown, note that dup15q contains loci both within and outside of the duplicated region. (C) The identified peaks in (B) were used to assess relative enrichment of overrepresented motifs within differentially accessible genomic regions. The venn diagram demonstrates the overlap of significant (i.e., *n*-fold enrichment >2 and *p*_adj_ < 0.05) identified overrepresented motifs. (D) FOS/JUN motifs were identified in the ASD-specific differentially accessible genomic regions. The per-cell-motif activity calculation revealed higher activity in neuronal subtypes (circled). Conversely, motifs involved in immune and inflammation signaling were enriched in peaks unique to dup15q, and corresponding microglial activity (circled) was observed. Motifs enriched from the “shared” group, (i.e., identified from differentially accessible sites present in both dup15q vs. control nuclei and ASD vs. control nuclei) lacked this cell-type specificity. Representative JASPAR matrix profiles MA0478.1 (FOSL2), MA.0837.1 (CEBPE), and PB0046.1 (Mybl1_1) are shown.

We examined global changes in DNA accessibility by using snRNA-seq and snATAC-seq samples (i.e., multi-omic sequencing) (Figure 5A) and identifying differentially accessible genomic regions between conditions (Figure 5B, Data S1) and overrepresented motifs (Figure 5C, Data S1). Although gene-expression analysis showed a greater number of DEGs in dup15q individuals versus control individuals, as compared to non-dup15q ASD versus control individuals, we identified twice as many differentially accessible peaks in non-dup15q ASD versus control individuals (Figure 5B), consistent with the conclusion that dup15q primarily affects a specific genetic region. Additionally, there were almost four times as many differentially accessible peaks that were unique to either comparison, suggesting divergent patterns of genome-wide regulation in ASD and dup15q (Figure 5B).

Understanding the regulatory modules in ASD and dup15q is important for clarifying the underlying neurobiology. For example, our analyses suggested that specific genomic regions demonstrating differential accessibility are largely unique in ASD and dup15q but that there could be shared transcriptional regulatory modules as a result of convergent biological processes. We thus assessed enrichment for specific transcription-factor motifs in the differentially accessible genomic regions that were unique to each condition vs. those that were shared. Most motifs were not unique to any peak list (Figure 5C); these 256 motifs most likely represent shared developmentally critical hubs of transcriptional regulation. This is consistent with their activity enrichment across different cell types in our dataset (Figure 5D). Among the top motifs that were unique to ASD (69 total) were FOS- and JUN-related sites; genome-wide analysis demonstrated that these sites showed preferential activation in neuronal subtypes (Figure 5D). Examining the top enriched motifs unique to dup15q (49 total) demonstrated a distinct pattern—motifs critical to immune and inflammatory signaling, such as CEBPE, ELF4 and IRF4, were evident. In contrast to the top ASD-unique motifs that demonstrated enrichment of neuronal activity, our analyses highlighted microglial activity of these dup15q-unique motifs (Figure 5D). Furthermore, our snRNA-seq data showed that among non-neuronal subtypes, microglia contained the highest number of differentially expressed genes in dup15q (Table S2). Additionally, gene-ontology results for microglia in dup15q vs. control individuals revealed biological processes relevant to synaptic pruning, which was absent from the ASD vs. control comparison (Figure S8). Thus, we uncovered an unexpected inflammatory signature unique to dup15-affected individuals.

Finally, our approach of incorporating snATAC-seq afforded the opportunity to explore the relationship between gene expression and chromatin accessibility in more depth. In general, we found that, in all nuclei, any given gene within the dup15q locus demonstrated variable peak linkage, and the number of peaks associated with any given gene did not clearly correlate with pTriplo (Figure S9). However, when we explored the dup15q vs. control condition, we identified a significant positive association between differential chromatin accessibility and gene expression for dup15q genes and peaks in comparisons of neurons to glia (Figure S10). Thus, global chromatin reorganization is associated with the cell-type-specific observed changes in gene expression in dup15q.

## Discussion

Here, we confirmed prior bulk RNA-seq work showing overall increased expression of genes within the region duplicated in dup15q brains^41^ but further identified unexpected cell-type-specific heterogeneity in expression changes of genes within the duplicated region, specifically implicating dysregulation of the PWACR in neurons. Gene-expression changes demonstrated different patterns of regulation not fully explained by gene dosage, function, or even imprinting status. Other reports of dup15q-derived neurons demonstrated that cell type rather than genotype was the main determining factor in gene-expression changes, findings our work agrees with.^35^^,^^42^ Unexpectedly, we found that the most highly expressed genes in any given cell type seemed to be spared from the most profound effects of increased gene dosage and that prior genome-wide metrics of dosage sensitivity did not necessarily capture gene-expression changes. Our work thus highlights the importance of unbiased and creative approaches in functionally validating the effects of copy-number variants directly in the tissue of interest in neurodevelopmental disorders.^43^^,^^44^

We also identified transcriptional regulatory networks implicating neuroinflammation and microglial function. However, microglial *CYFIP1*, a gene within the duplicated region, did not demonstrate evidence for significant regulation in dup15q-affected individuals, despite the fact that our study was well-powered to detect such changes in this cell type. Our work, which is observational in nature, cannot parse whether the microglial signal observed here represents a direct effect of the duplication or a response by the brain to dysfunction in other cell types. Future experimental work will be required to address this. This finding is nonetheless intriguing in light of the emerging role of microglia in brain disorders across the lifespan.^38^^,^^45^^,^^46^^,^^47^ We also resolved notable cell-type-specific changes in expression of the GABA receptor cluster within the duplicated region. Given the close genetic proximity of the three receptors, one might anticipate shared regulatory mechanisms, but we identified the most prominent dysregulation of GABRB3 in dup15q. This could be related to several factors, including regional differences that occur in the epigenetic landscape and are unique to frontal cortex or subtle parent-of-origin effects that persist despite biallelic expression.^48^ Given findings that individuals with dup15q have an EEG biomarker mimicking increased GABAergic signaling, as well as recent clinical trials that target GABA transmission, this information could prove useful in guiding future precision therapeutics.^49^^,^^50^

Reassuringly, our work is concordant with past studies, which reinforces the biological plausibility of our unexpected findings of cell-type specificity of dup15q. For example, our multi-omic sequencing revealed enrichment of JUN and FOS motifs in differentially accessible regions in ASD but not in dup15q. This is consistent with existing literature implicating these networks in neurodevelopmental disorders, including ASD.^51^^,^^52^ Replication of independent findings of enrichment of SFARI ASD risk genes and FMR1 protein networks, as well as layer 2/3 neuronal dysregulation in the pathogenesis of neurodevelopmental disorders, also indicated that our approach detected meaningful biological perturbations.^7^^,^^9^^,^^32^^,^^35^^,^^41^ Indeed, results here confirmed independent laboratories’ work using cellular models, as well as past experiments in human brain bulk tissue.^6^^,^^42^

Our data show that the effects of copy-number duplications are not straightforward. Given that dup15q is a germline event, it was unexpected that neurons demonstrated more marked dysregulation of gene expression within the duplicated region than glia. Although this implicates neuronal dysfunction in dup15q pathogenesis, it also provokes questions about what endogenous factors related to the epigenetic landscape moderate the effects of this germline structural variant. It is interesting that we found that cell types with high gene expression at baseline may conversely engage homeostatic mechanisms to minimize further increases, which could be potentially toxic to the cell. Better understanding of these differences could be used in the future to guide therapeutic strategies, for example through the harnessing of glial homeostatic mechanisms to tamp down effects of the duplication in neurons. Our unbiased, empiric identification of cell-type-specific changes in gene expression and the epigenetic landscape can also guide future therapeutic work. For example, non-canonical cell types might be critical in mediating the pathophysiology of dup15q and thus could be targeted in future precision-medicine therapeutic interventions, results that could easily be otherwise missed.

Limitations of this report include a small sample size of affected individuals. Thus, certain features, such as individual variability of dup15q or the effects of the duplication on very rare cell types, cannot be completely parsed from our methodological approach. However, our main findings, of unexpected cell-type-specific determinants of the effect of dup15q, are unlikely to be artifacts of our sample size. Our “low-resolution” analysis of glia and neurons corroborate the findings of the more subtle “high-resolution” approach. It is possible that the unique microglial inflammatory signature in dup15q is an artifact of the relatively high nuclei number that this cell type comprises. This is unlikely given that other cell types (astrocytes, for example) are present at similar or even higher proportions and were not implicated in multi-omic analysis in the same way. Future work will be needed to study whether the changes we identified are a direct result of alterations in the integrity of the imprinting center or more general genomic alterations. Additionally, we identified significant individual heterogeneity even within the dup15 cases, and this finding warrants future investigation into how genome-wide determinants might influence the effects of dup15, such as single-nucleotide polymorphisms. Nonetheless, as an important report of the cell-type-specific effects of copy-number gains in the human brain in neurodevelopmental disorders, our work reveals unexpected gene-expression changes that could fuel novel paths of investigation for therapeutic approaches. We also demonstrate the importance of direct, unbiased, and cell-type-specific studies in the human brain to complement mouse and *in vitro* cellular models.

### Conclusions

We identified marked cell-type-specific regulation of gene expression and chromatin accessibility in human brain specimens from dup15q vs. non-dup15q ASD individuals and neurotypical control individuals, both within the region of the duplication and genome-wide. This work demonstrates that gene-expression changes caused by copy-number variants in neurodevelopmental disorders result from a complex interplay of factors as opposed to simply reflecting gene dosage. Neurons demonstrate marked evidence of altered gene expression in dup15q, particularly within the PWACR. We also identified evidence of an unexpected microglial inflammatory response unique to dup15q. This unexpected pattern of genomic regulation demonstrates the importance of directly studying the functional effects of germline genetic structural variants in the human brain in neurodevelopment, particularly in the context of copy-number gains that might behave unexpectedly. Our findings also demonstrate the importance of studying primary brain tissue directly with techniques that afford cell-type specificity and unbiased approaches. Our work will guide future mechanistic studies as well as rational therapeutic development in dup15q.

## Data and code availability

Sequencing data are made available to the research community as per the informed consent of the donors and their families. Data from the NIH NeuroBioBank is available through dbGAP (accession number phs000639v3p1). Data from Autism BrainNet is deposited in SFARI Base, a publicly available database accessible at https://www.sfari.org/resource/sfari-base/.

## Supplemental information

Document S1. Figures S1–S10, Tables S1–S3, and supplemental methods

Data S1. Supplemental Excel and CSV files

Document S2. Article plus supplemental information
