# Supplementary material for: Cell-type-specific effects of autism-associated 15q duplication syndrome in the human brain
Source: Am J Hum Genet. 2024 Jul 29;111(8):1544–58. doi: 10.1016/j.ajhg.2024.07.002 (PMC11339625; doi:10.1016/j.ajhg.2024.07.002)
Supplement: Document S1. Figures S1–S10, Tables S1–S3, and supplemental methods [file mmc1.pdf]

The American Journal of Human Genetics, Volume *111*

Supplemental information

Cell-type-specific effects of autism-associated 15q duplication syndrome in the human brain

Caroline Dias, Alisa Mo, Chunhui Cai, Liang Sun, Kristen Cabral, Catherine A. Brownstein, Shira Rockowitz, and Christopher A. Walsh

Supplemental Figures

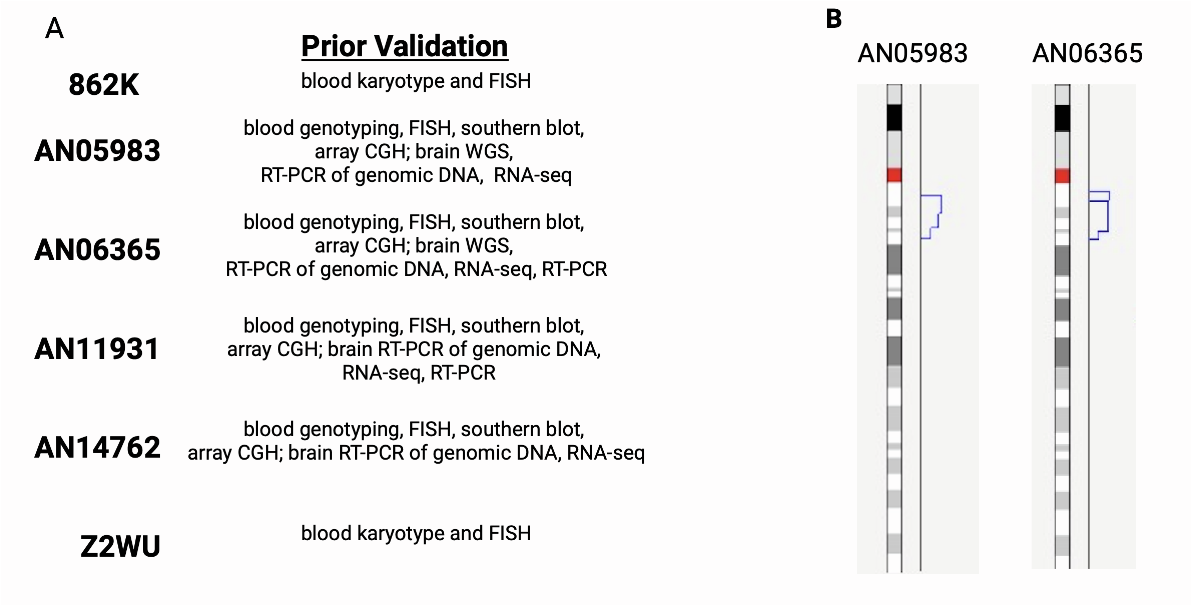

Figure S1: Dup15q sample validation. (All samples have tetrasomy of the PWACR) **A.** Dup15q samples have been extensively validated in the literature[6,41]. **B.** Two samples were also validated with optical genome mapping. Note that the ideogram represents only the copy number changes and not the chromosomal structure.

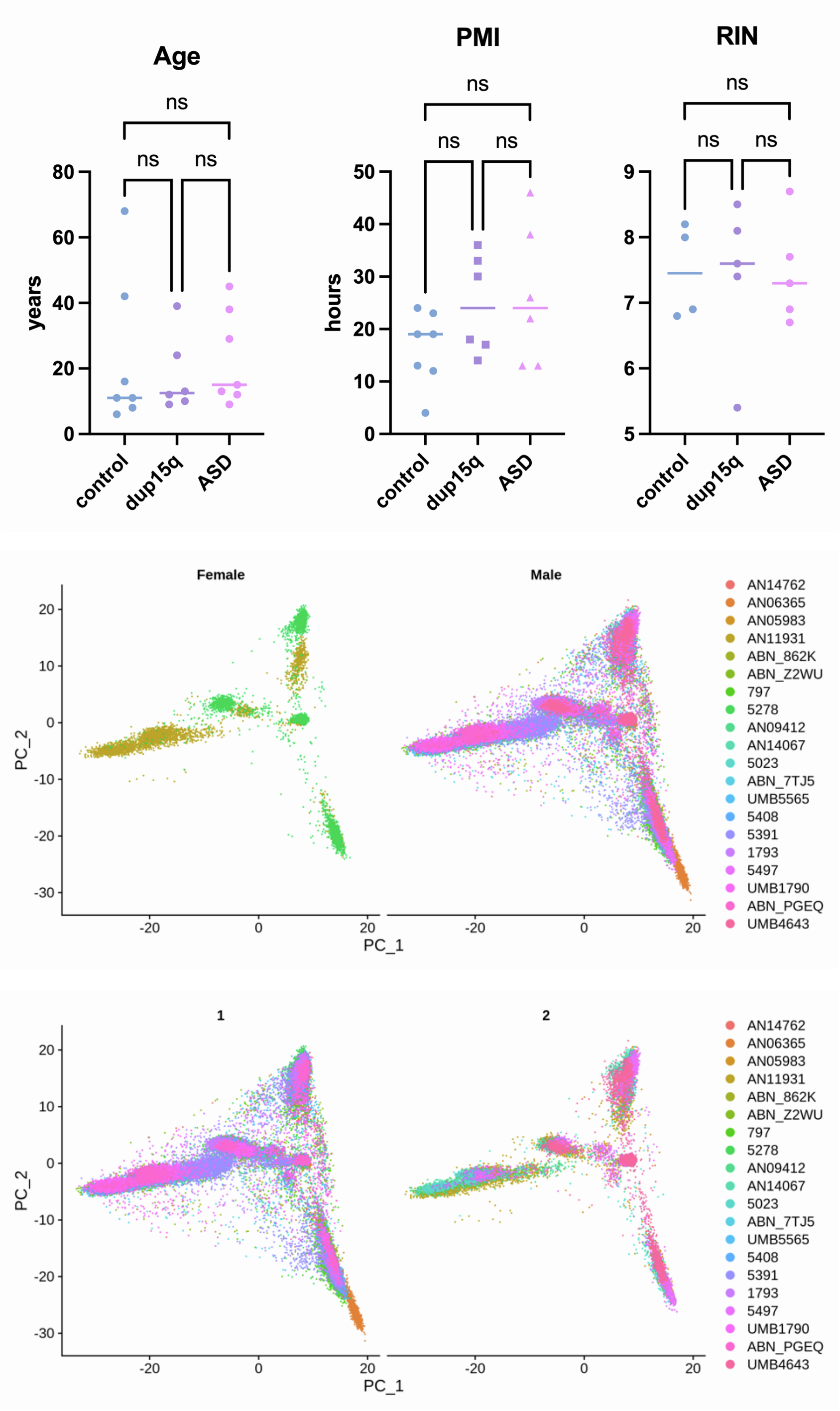

Figure S2: Impact of demographics. Top panel: Sample comparison. No significant differences in age, PMI or RIN. ($p > .05$, 1 way ANOVA, Tukey post-hoc test. There was also no difference in the female percentage between groups ($p > .05$, chi-square). Middle and bottom panel: PCA plots demonstrate sex and age (split as < and > 21 years of age) are not major determinants of gene expression variability.

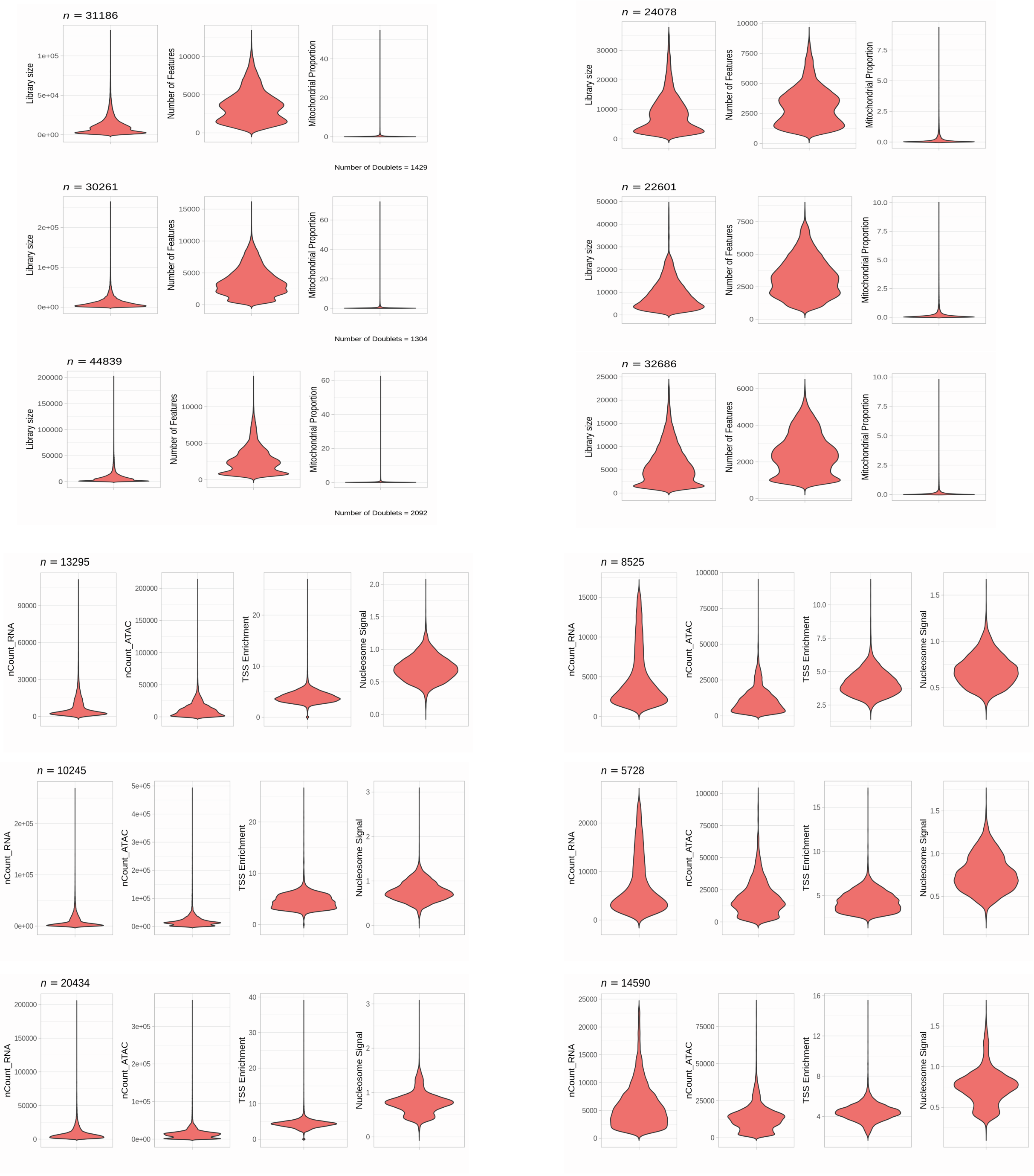

Figure S3: Quality control and filtering. snRNA-seq (top 3) and multi-omic (bottom 3) quality metrics of raw (left) and filtered (right) nuclei. Each panel demonstrates library size, gene number, and proportion of mitochondrial genes (from left to right) and for ATAC-seq- ncount RNA, ATAC, TSS enrichment and nucleosome signal are shown. Top is dup15q, middle is ASD, and bottom is control. Number of nuclei in top left corner, number of doublets in each group prior to filtering also indicated.

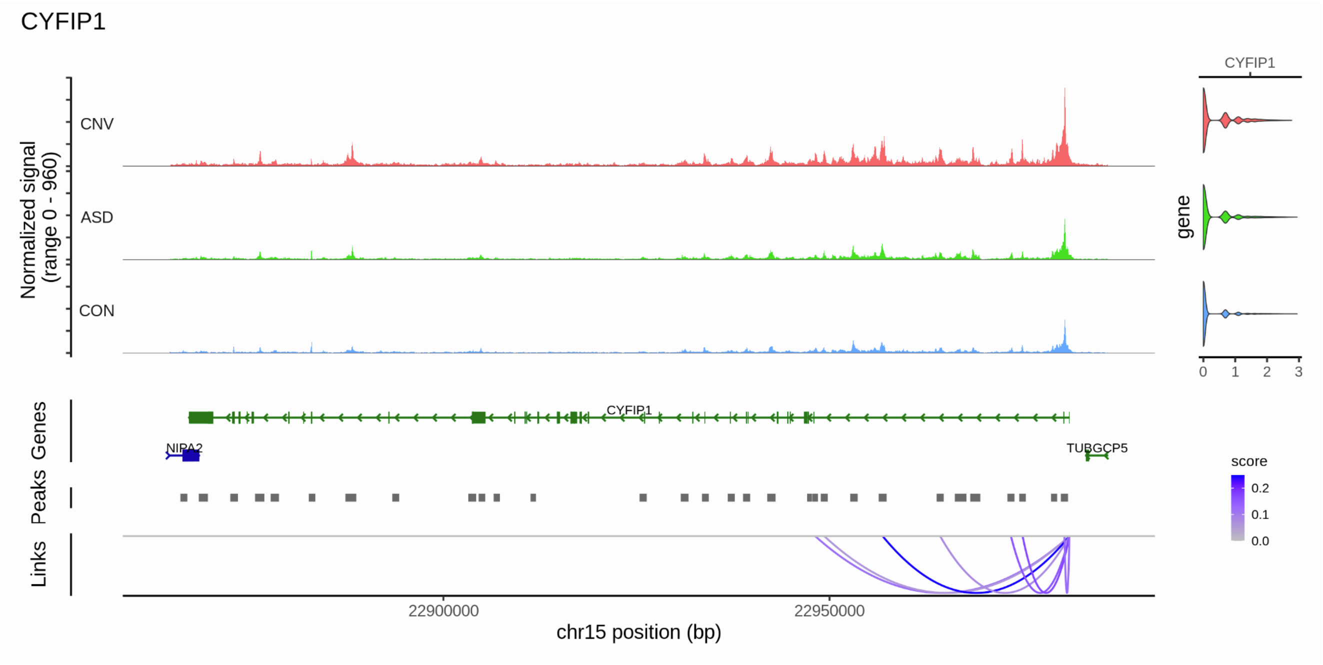

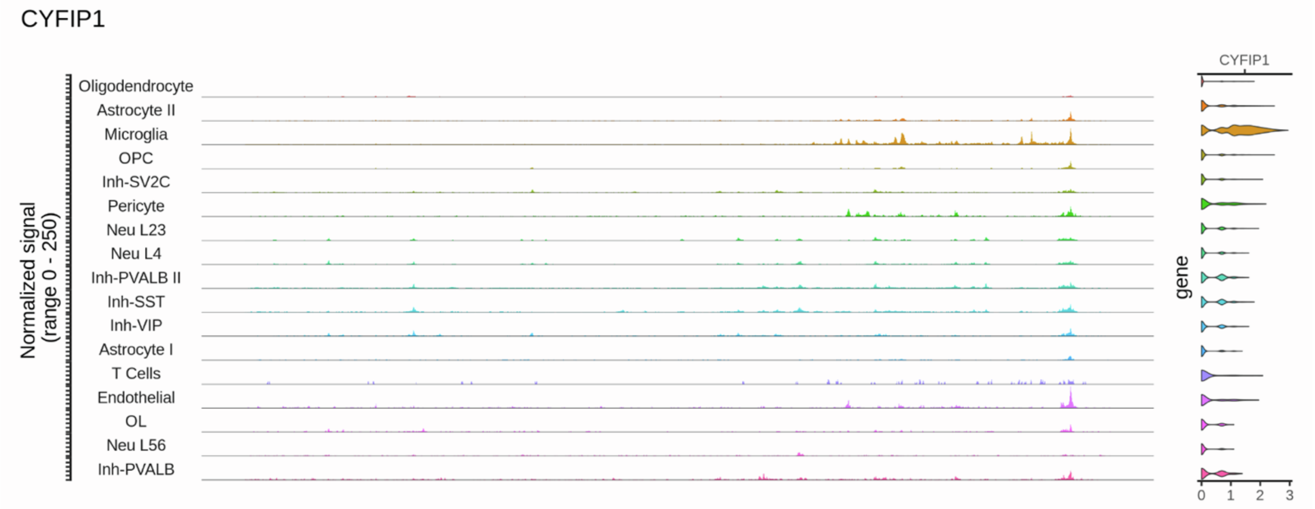

Figure S4: *CYFIP1* chromatin accessibility. Increased expression and chromatin accessibility at *CYFIP1* in dup15q samples shown, as well as peak to peak linkage and cell-type-specific chromatin accessibility. Top panels represent signal from all nuclei combined.

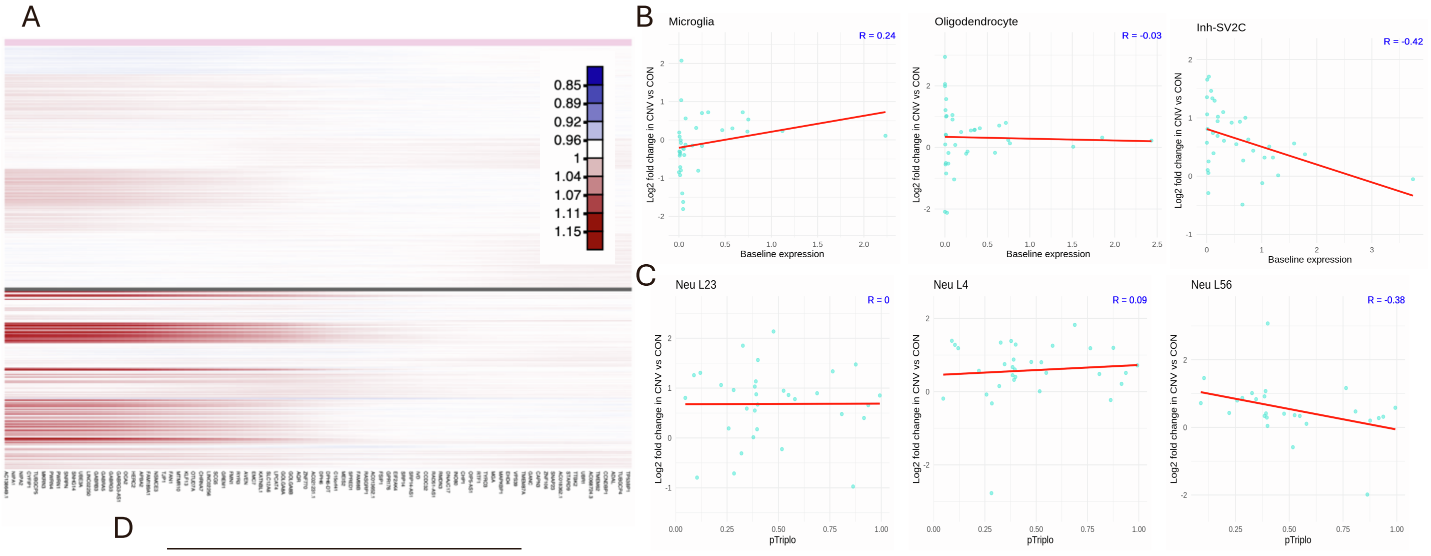

D

Gene	pTriplo
TUBGCP5	0.3846449
CYFIP1	0.87568458
NIPA1	0.52507227
NIPA2	0.39983122
MAGEL2	0.35396615
SNRPN	0.22130561
UBE3A	0.93655862
ATP10A	0.68789539
GABRB3	0.99263489
GABRA5	0.32659342
GABRG3	0.39635259
OCA2	0.08859845
HERC2	0.7635295
APBA2	0.38461863
TJP1	0.91646147
KLF13	0.47624642
OTUD7A	0.80825112
CHRNA7	0.57981061

Figure S5. Assessing mediating factors to dup15q expression changes. A. inferCNV reveals heterogeneous expression increases in region of duplication in dup15q cases (below grey line) across samples as compared to control (above grey line) within all nuclei (each row). Heatmap color scale indicates decreased (blue) or increased expression. **B.** Cell-type-specific examples of the association between baseline expression in controls and the fold change expression in dup15q. Within the duplicated region, genes that are highly expressed in different cell types demonstrate modest changes in expression in dup15q cases. p-values are non-significant except for in Inh-SV2C (p=.01, not adjusted for multiple comparisons) **C.** Association of pTriplo in cell-type-specific examples. All p-values > .050. **D.** pTriplo metric for select dup15q genes

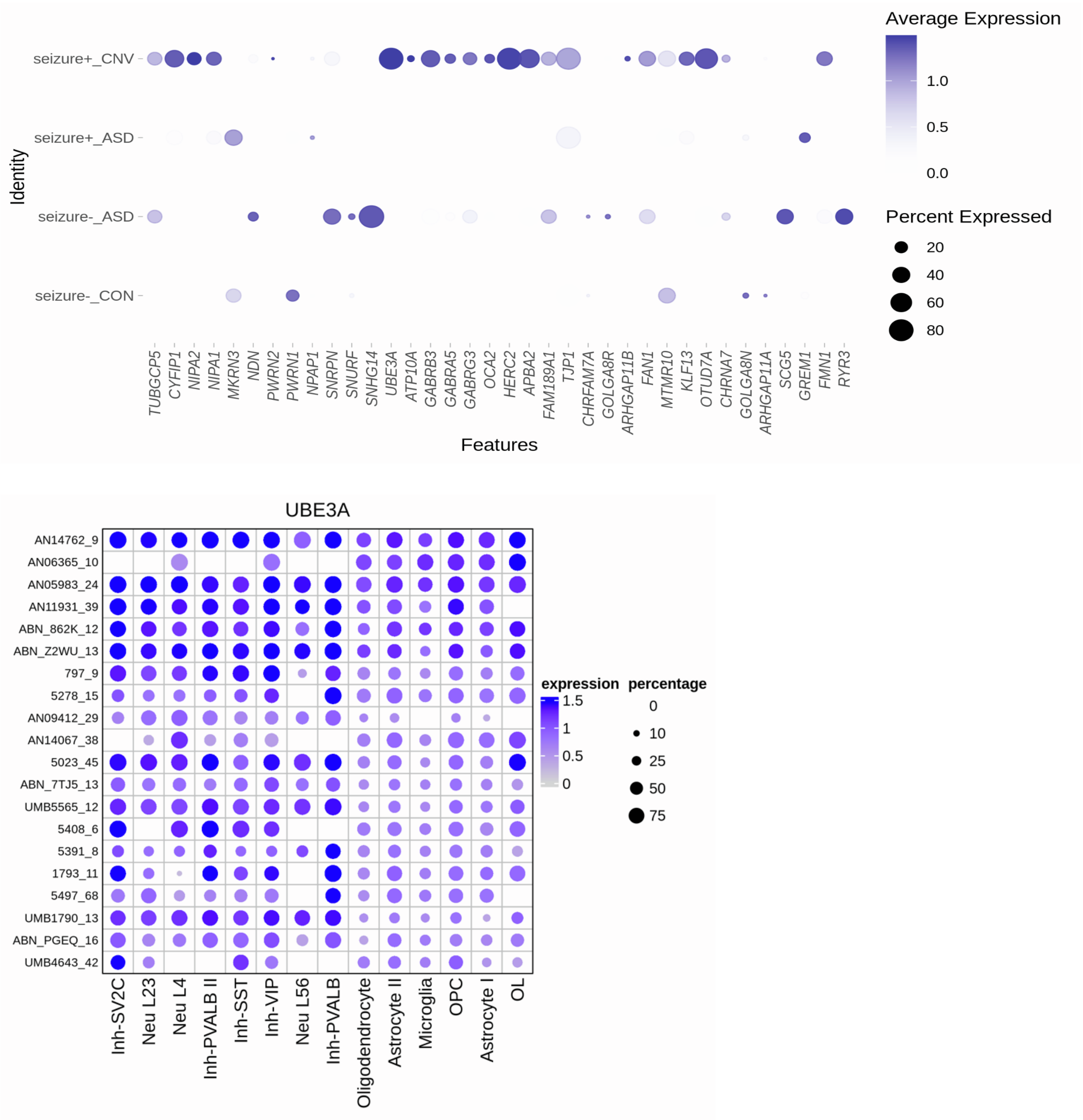

Figure S6: Impact of seizure diagnosis or other sources of individual variability on dup15q gene expression changes. Top: Presence of seizure diagnosis in ASD cases does not recapitulate dup15q gene expression changes observed within the duplicated (CNV) region as visualized with dot plot. Bottom: UBE3A expression is separated out by each individual sample (sample_age) and cell type to demonstrate that although there is notable heterogeneity between samples, effects are not being driven by an individual sample or factor such as age.

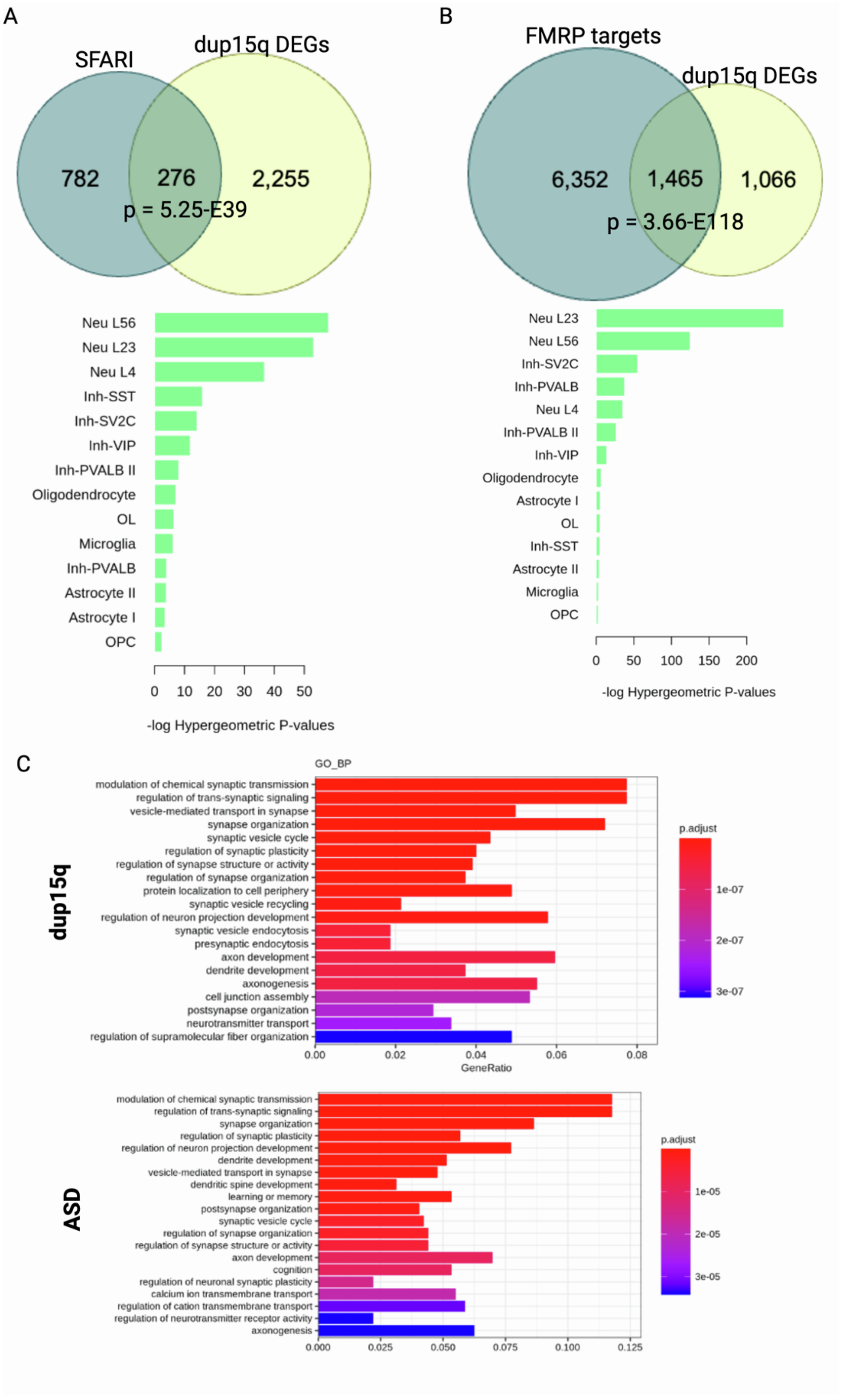

Figure S7: Neurobiological substrates in dup15q. Overlap of known (A) SFARI pathogenic ASD risk genes as well as (B) FMR1 protein target genes, demonstrates significant enrichment (p-value indicated in figure, hypergeometric test) These genes demonstrate notable enrichment in excitatory neuron subtypes. **C.** Using gene ontology analysis, we also found evidence for biological process terms involved in synaptic function. Presented are results from differential expression analysis in layer 2/3 neurons in each condition vs. control.

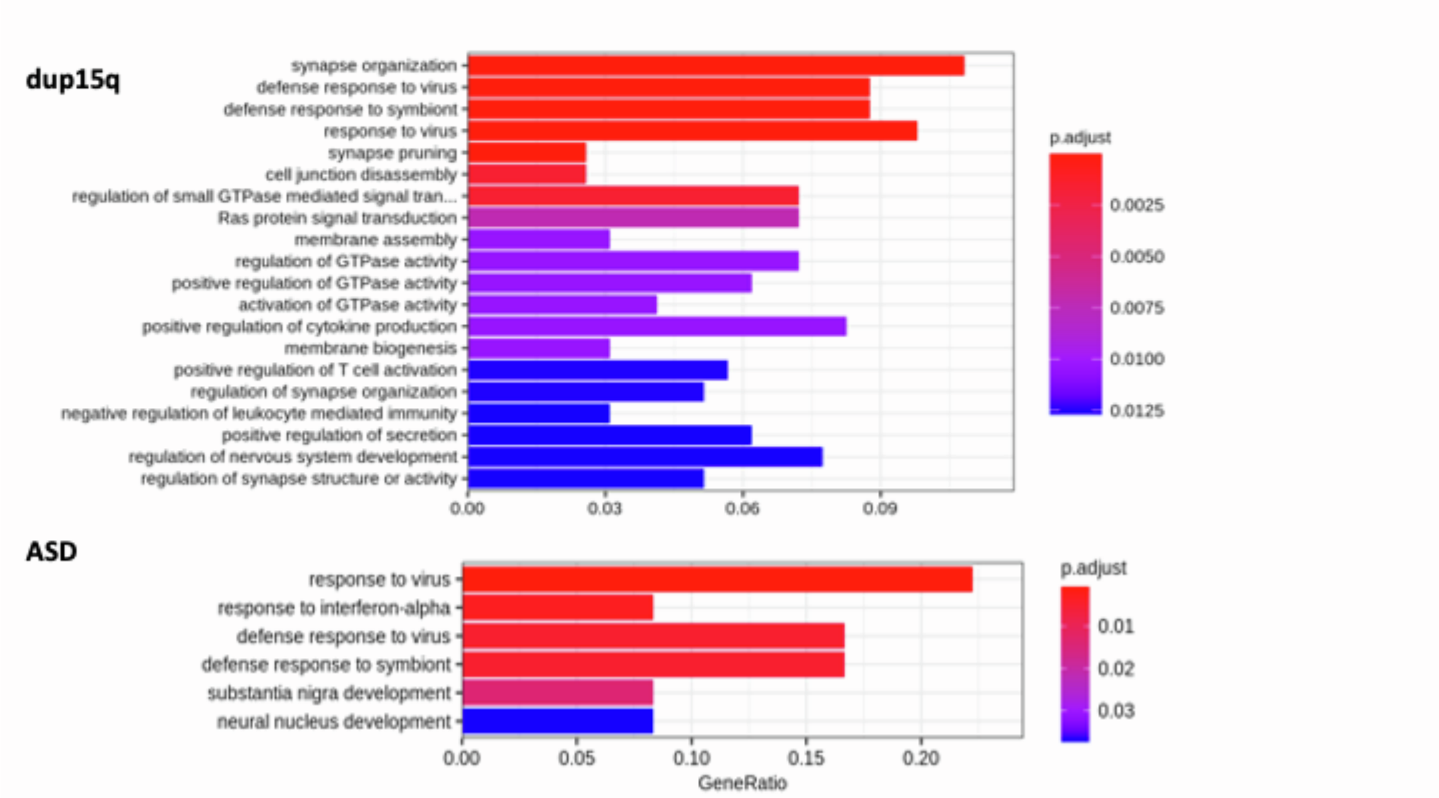

Figure S8: Microglia gene ontology analysis. Using gene ontology analysis, we found evidence suggesting dysregulation of synaptic pruning only in dup15q microglia, but not ASD (compared to control).

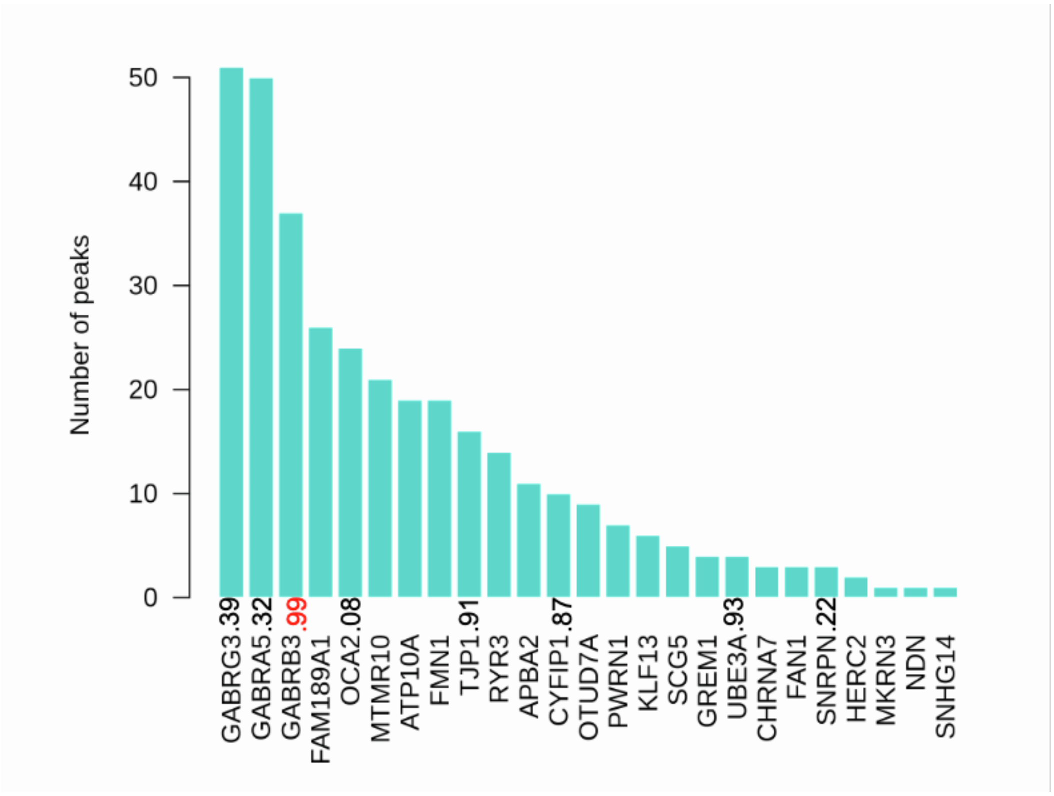

Figure S9: Dup15q gene-peak analysis. The LinkPeaks functionality in Signac was applied to the integrated multi-omic data-set, to identify significant peaks associated with genes within the duplicated region, which was calculated given GC content, accessibility, and length of the peak. The default thresholding was used which included p value < .05, Z-score < .05, and distance = 500,000 base pairs. There was variability in the number of peaks associated with genes within the duplication; there was not a clear correlation between number of peaks and pTriplo (Select pTriplo metrics shown above gene name for clarity, with significant pTriplo score in red.)

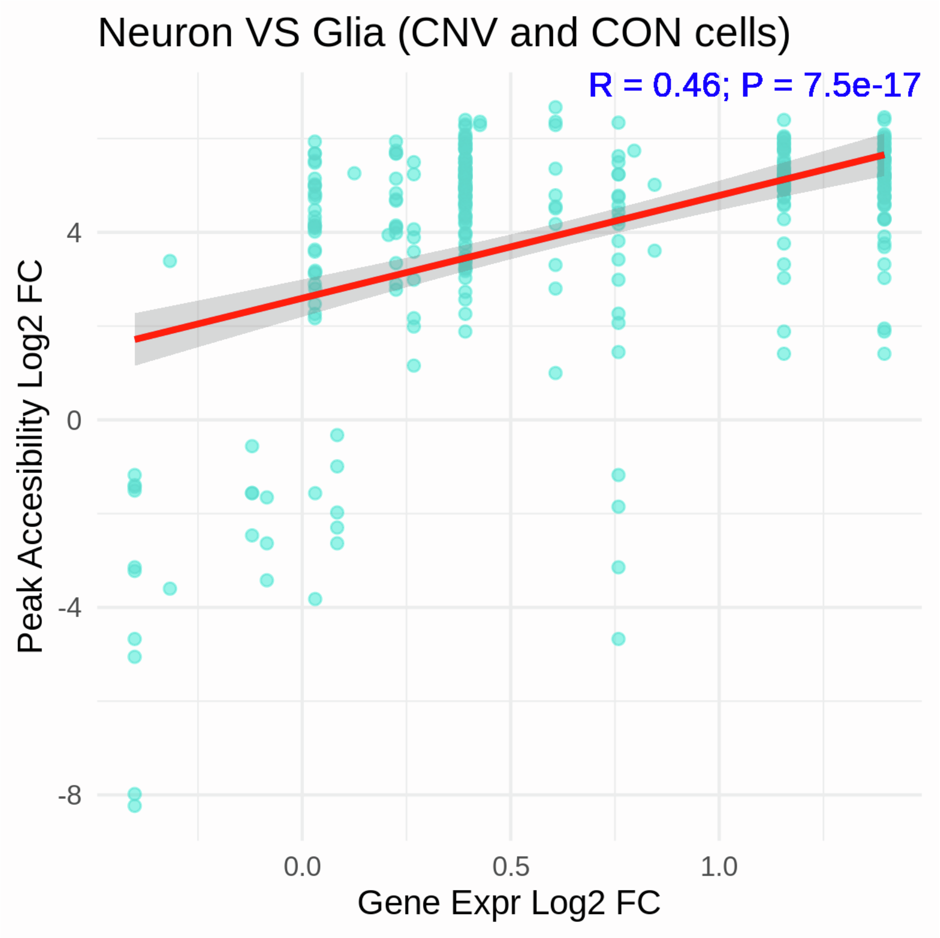

Figure S10: Association between differential chromatin accessibility and gene expression for peaks significantly associated with dup15q genes in the CNV vs Control comparison. Correlation analysis demonstrated a significant positive relationship (R=0.46, p < .001) between differential peak accessibility and gene expression for dup15q associated genes/peaks in neuron vs. glia comparison. After exclusion of ASD samples, the intersection of dup15q DEG lists between 1) CNV and control and 2) neurons and glia (both padj <.05 using FindMarkers function) was used to then create a list of linked peaks (with the LinkPeaks function) associated with dup15q region DEGs. Significantly differentially accessible peaks for the above DEG associated peaks were identified as above. Finally, the fold change expression in dup15q region DEGs was correlated with the differential accessibility of the associated peaks.

Supplemental Tables

Logfc = 0.2	# DEGs (all cells)	# DEGs (neuron cells)	# DEGs (glia cells)
CNV vs CON	126	623	206
ASD vs CON	34	329	45
CNV vs ASD	52	101	113

Table S1: **Differentially expressed genes.** Number of significant differentially expressed genes per comparison.

Cell Type	# DEGs	# Upreg	# Downreg	Cell Type	# DEGs	# Upreg	# Downreg
Non-neuronal				Neuronal			
Pericyte	48	23	25	Neu L56	3	2	1
Endothelial	11	7	4	Neu L4	308	239	69
Microglia	240	124	116	Neu L23	1,307	1,162	145
Astrocyte I	115	60	55	Inh-SV2C	315	290	25
Astrocyte II	124	82	42	Inh-VIP	107	84	23
Oligodendrocyte	119	78	41	Inh-PVALB	175	165	10
OL	5	3	2	Inh-SST	74	40	34
OPC	22	14	8	Inh-PVALB II	205	193	12
T Cells	1	1	0				

Table S2: **Cell-type-specific differentially expressed genes.** Number of significant differentially expressed genes in high resolution differential expression analysis, in dup15q vs control comparison (p-value adj<0.05).

Cell Ranger (RNA-seq)	Estimated Number of Cells	Mean Reads per Cell or ATAC Mean raw read pairs per cell	Median Genes per Cell or ATAC # peaks	Number of Reads or ATAC sequenced read pairs
AN14762	4,545	62,551	3,226	284,295,419
AN06365	3,823	79,570	3,669	304,195,930
AN05983	5,447	71,327	4,181	388,517,150
AN11931	4,336	67,944	5,156	294,603,243
797	6,304	63,125	3,064	397,937,622
5278	5,781	144,309	3,778	834,250,234
AN09412	1,458	176,120	3,969	256,783,196
AN14067	3,036	102,840	3,491	312,221,137
5023	4,285	65,205	3,529	279,402,186
5408	4,390	59,474	3,149	261,092,263
5391	11,489	29,686	1,052	341,060,553
1793	3,151	73,108	3,218	230,363,514
5497	5,557	72,214	3,029	401,292,261
**Arc (ATAC-seq)**				
ABN_862K	6019	41998.3567	107796	252,788,109
ABN_Z2WU	7276	38336.3718	184784	278,935,441
ABN_7TJ5	5867	40012.9637	195266	234,756,058
UMB5565	4378	59595.0763	155245	260,907,244
UMB1790	7189	34747.195	209764	249,797,585
ABN_PGEQ	4918	48866.7477	133719	240,326,665
UMB4643	8327	27741.7383	129083	231,005,455

Table S3: Technical sample information. Read coverage breakdown by sample for snRNA-seq and snATAC-seq components as indicated from Cell Ranger and Arc output.

Supplemental Methods

Optical Mapping of genomic DNA from post-mortem brain: 15-20 mg of brain tissue was homogenized for 10 seconds in Bionano Genomics Homogenization Buffer (P/N 20406) using a TissueRuptor, passed through a 40μm filter, then pelleted at 2000 x g for 5 minutes. The pellet was resuspended and re-pelleted from Bionano Genomics Wash Buffer A (P/N 20407). Homogenized tissue was then digested in 50μL of Protease solution and Bionano Genomics Lysis and Binding Buffer (LBB) (P/N 20375) on a HulaMixer for 15 minutes at 10 rpm. Tissue was further digested in Proteinase K on a HulaMixer for 15 minutes at 10 rpm. Enzymes were inhibited by PMSF.

gDNA binding was performed in the presence of Bionano Genomics Salting Buffer (P/N 20404) and 100% Isopropanol to a Bionano Genomics Nanobind Disk (P/N 20402) on a HulaMixer for 30 minutes at 10 rpm. Nanobinds were washed with Bionano Genomics Wash Buffer 1 (P/N 20376) and Wash Buffer 2 (P/N 20377) (2 rounds each) using a Dynamag Tube Rack. gDNA was eluted from Nanobind using Bionano Genomics Elution Buffer (P/N 20378). gDNA was homogenized for 1 hour at 15 rpm and incubated at room temperature for at least 3 days before labeling and staining. gDNA was fluorescently labeled and stained using the Bionano Genomics Direct Label and Stain Kit (P/N 80005) following Bionano Prep Direct Label and Stain protocol (P/N 30206).

Data was collected on the Saphyr instrument to a target throughput of 1500 Gbp or 400X effective coverage of the GRCh38 reference. Data was submitted to the Rare Variant Analysis pipeline on Bionano Solve version 3.6.1. The Rare Variant Pipeline aligns molecules to the reference to detect relative differences in the sample, forms consensus maps of molecule support in these loci and in the presence of sufficient molecule support, a structural variant call is made. The molecule data was subsequently down-sampled to 400Gbp or 100X coverage of the GRCh38 reference and submitted to the De Novo Assembly pipeline in Bionano Solve version 3.6.1 during which molecules were assembled de novo to form haplotype consensus maps, which were then aligned to the GRCh38 reference for structural variant detection and calling. In both scenarios, global reference coverage is used to determine copy number aberrations in addition to the aforementioned structural variant calling. Results were reviewed in Bionano Access 1.6.1. During review, structural variant and copy number calls were filtered against the Bionano provided mask files which remove structural variant and copy number calls in complex or poorly constructed regions of the GRCh38 reference. Variant calls were filtered against the Bionano control database of healthy individuals retaining only variants present in less than 1% of the control database. Variants were also filtered according to recommended confidence score filtering; 0 for indels, 0.7 for inversions, 0.3 for intrachromosomal fusions, 0.65 for intrachromosomal translocations, and no confidence scores were calculated for duplications where a placeholder value of -1 is used. Variants remaining after filtering were further curated by careful review of the data.
