# Supplementary material for: Cell-type-specific effects of autism-associated 15q duplication syndrome in the human brain
Source: Am J Hum Genet. 2024 Jul 29;111(8):1544–58. doi: 10.1016/j.ajhg.2024.07.002 (PMC11339625; doi:10.1016/j.ajhg.2024.07.002)
Supplement: Document S2. Article plus supplemental information [file mmc3.pdf]

Cell-type-specific effects of autism-associated 15q duplication syndrome in the human brain

Authors

Caroline Dias, Alisa Mo, Chunhui Cai, ..., Catherine A. Brownstein, Shira Rockowitz, Christopher A. Walsh

Correspondence

caroline.dias@childrenscolorado.org (C.D.), christopher.walsh@childrens.harvard.edu (C.A.W.)

Recurrent copy-number variation represents a well-established genetic driver in neurodevelopmental disorders. We applied single-nucleus approaches to postmortem brain tissue from individuals with autism-associated dup15q ($n = 6$), autism ($n = 7$), and neurotypical controls ($n = 7$). We found unexpected cell-type-specific gene expression and chromatin accessibility changes that will guide future research and therapeutic development.

Dias et al., 2024, The American Journal of Human Genetics *111*, 1544–1558

August 8, 2024 © 2024 The Authors.

https://doi.org/10.1016/j.ajhg.2024.07.002

ARTICLE

Cell-type-specific effects of autism-associated 15q duplication syndrome in the human brain

Caroline Dias,[1,2,3,7,8,*] Alisa Mo,[4,8] Chunhui Cai,[5] Liang Sun,[5] Kristen Cabral,[2] Catherine A. Brownstein,[2,3] Shira Rockowitz,[2,5] and Christopher A. Walsh[2,3,4,6,*]

Summary

Recurrent copy-number variation represents one of the most well-established genetic drivers in neurodevelopmental disorders, including autism spectrum disorder. Duplication of 15q11–q13 (dup15q) is a well-described neurodevelopmental syndrome that increases the risk of autism more than 40-fold. However, the effects of this duplication on gene expression and chromatin accessibility in specific cell types in the human brain remain unknown. To identify the cell-type-specific transcriptional and epigenetic effects of dup15q in the human frontal cortex, we conducted single-nucleus RNA sequencing and multi-omic sequencing on dup15q-affected individuals ($n = 6$) as well as individuals with non-dup15q autism ($n = 7$) and neurotypical control individuals ($n = 7$). Cell-type-specific differential expression analysis identified significantly regulated genes, critical biological pathways, and differentially accessible genomic regions. Although there was overall increased gene expression across the duplicated genomic region, cellular identity represented an important factor mediating gene-expression changes. As compared to other cell types, neuronal subtypes showed greater upregulation of gene expression across a critical region within the duplication. Genes that fell within the duplicated region and had high baseline expression in control individuals showed only modest changes in dup15q, regardless of cell type. Of note, dup15q and autism had largely distinct signatures of chromatin accessibility but shared the majority of transcriptional regulatory motifs, suggesting convergent biological pathways. However, the transcriptional binding-factor motifs implicated in each condition implicated distinct biological mechanisms: neuronal JUN and FOS networks in autism vs. an inflammatory transcriptional network in dup15q microglia. This work provides a cell-type-specific analysis of how dup15q changes gene expression and chromatin accessibility in the human brain, and it finds evidence of marked cell-type-specific effects of this genetic driver. These findings have implications for guiding therapeutic development in dup15q syndrome, as well as understanding the functional effects of copy-number variants more broadly in neurodevelopmental disorders.

Introduction

The proximal end of the long arm of chromosome 15 is a genomic region containing several segmental duplications, making it particularly susceptible to complex rearrangements at recurrent breakpoints (Figure 1A).[1] Parental chromosome-specific deletions in this region lead to the imprinting disorders Angelman syndrome (MIM: 105830) and Prader-Willi syndrome (MIM: 176270), whereas maternal duplication of 15q11–q13, referred to hereafter as dup15q syndrome (MIM: 608636), is associated with autism spectrum disorder (ASD), intellectual disability, hypotonia, and epilepsy.[2] There is over a 40-fold increased risk of ASD in individuals carrying this duplication, making it one of the most significant and highly penetrant genetic drivers of ASD.[3,4]

Most cases of dup15q are caused by an isodicentric chromosome 15 (Figure 1B),[5] and many of these are in turn driven by an asymmetric recombination event between breakpoints 4 and 5. This leads to tetrasomy from the centromere to breakpoint 4 (a region that includes the Prader-Willi/Angelman critical region [PWACR] between breakpoints 2 and 3) and trisomy from breakpoint 4 to 5 (Figure 1A).[2] Given that most cases are caused by maternally derived duplications and that the duplication contains a critical imprinting locus within the PWACR, copy-number alterations in maternally imprinted, dosage-sensitive genes such as *UBE3A* (MIM: 601623) have been hypothesized to underlie the dup15q clinical phenotype. However, the dup15q region also contains a GABA receptor cluster, and some individuals with dup15q have atypical responses to benzodiazepines, potentially implicating these and other genes as well.

The cell-type-specific effects of dup15q on gene expression in the human brain are unknown. Prior work in postmortem brains has shown that in bulk tissue, *UBE3A* expression does not match changes in gene dosage in dup15q-affected individuals.[6] However, bulk brain tissue analysis cannot identify whether only specific cell types showed changes in gene expression. Understanding how

[1]Division of Developmental Medicine, Boston Children's Hospital, Boston, MA 02115, USA; [2]Division of Genetics and Genomics, Manton Center for Orphan Disease Research, Boston Children's Hospital, Boston, MA 02115, USA; [3]Department of Pediatrics, Harvard Medical School, Boston, MA 02115, USA; [4]Department of Neurology, Boston Children's Hospital, Harvard Medical School, Boston, MA 02115, USA; [5]Research Computing, Department of Information Technology, Boston Children's Hospital, Boston, MA 02115, USA; [6]Howard Hughes Medical Institute, Boston Children's Hospital, Boston, MA 02115, USA
[7]Present address: Department of Pediatrics, Section of Developmental Pediatrics, Section of Genetics and Metabolism, Children's Hospital Colorado, University of Colorado Anschutz Medical Campus, Aurora, CO 80045, USA
[8]These authors contributed equally
*Correspondence: caroline.dias@childrenscolorado.org (C.D.), christopher.walsh@childrens.harvard.edu (C.A.W.)
https://doi.org/10.1016/j.ajhg.2024.07.002.
© 2024 The Authors. This is an open access article under the CC BY license (http://creativecommons.org/licenses/by/4.0/).

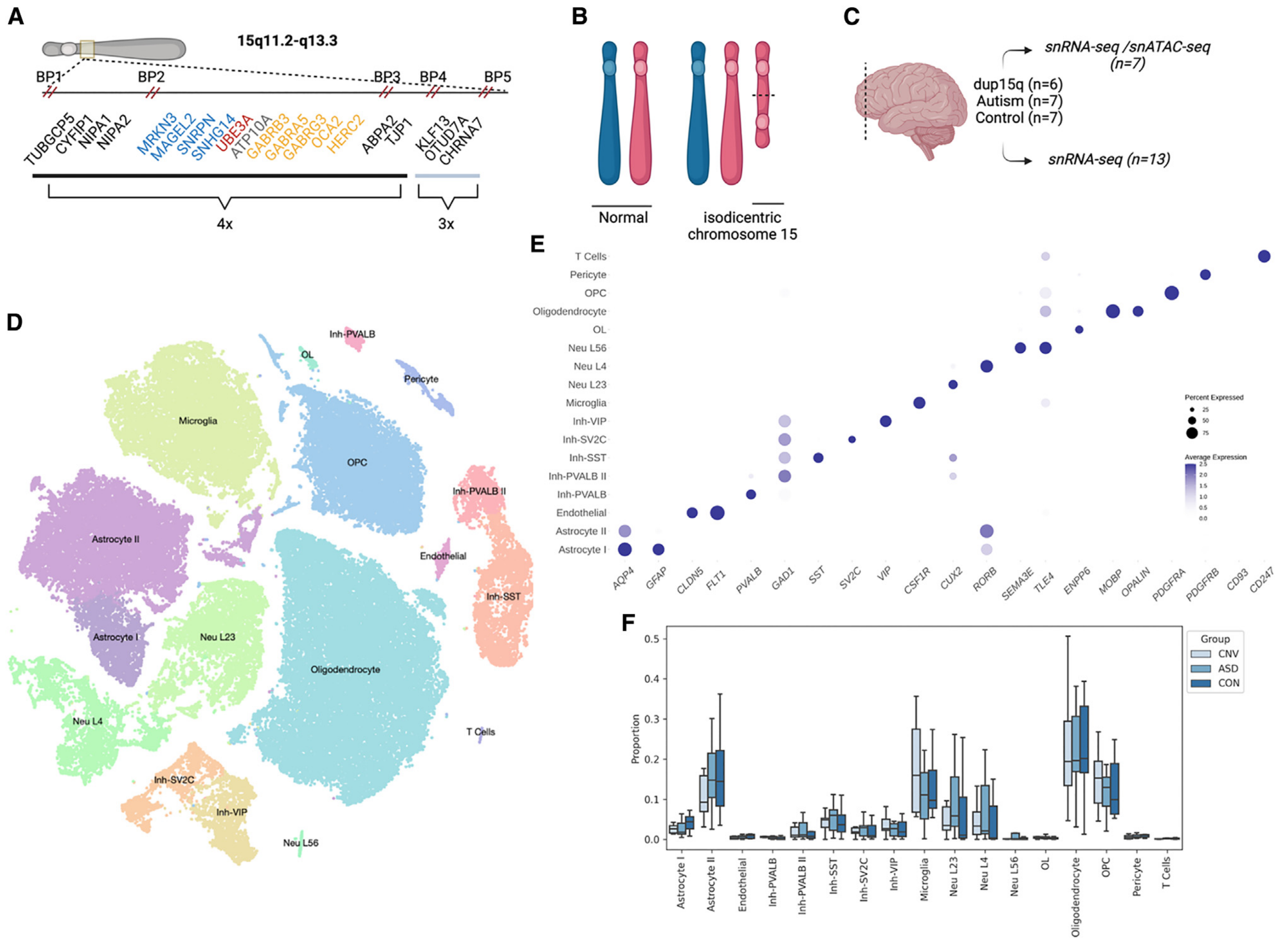

Figure 1. Single-nucleus analysis of dup15q in the human brain

(A) Genomic architecture of chromosome 15 with only select genes shown for clarity. Inset shows the location of genes, with breakpoints and copy-number indicated. Critical imprinting locus between breakpoints 2 and 3 (BP2 and BP3). Blue: paternally imprinted. Red: maternally imprinted. Yellow: bi-allelic. Gray: conflicting reports.

(B) Cartoon representation of dup15q structure due to isodicentric maternal chromosome 15.

(C) Samples used from frontal cortex.

(D) Visualization of single-nucleus data with t-SNE dimension reduction.

(E) Cell-type-specific gene expression indicates representation of expected cell types in human frontal cortex and accurate clustering.

(F) Cell-type compositional analysis indicates no significant changes in proportion of cell types between conditions. Boxplot shows median, interquartile range (box), and ±1.5 interquartile range (error bars) of the proportion of each cell type.

cell-type-specific heterogeneity in gene expression and chromatin accessibility contributes to pathophysiology is the first step in identifying potential therapeutic targets and advancing precision medicine for this disorder.

It is also critical to understand the similarities and differences on the molecular and cellular level between dup15q and ASD not related to dup15q. Although prior studies have demonstrated similarities on the molecular level in the brain between individuals with dup15q and ASD, suggesting convergent biological processes,[7–9] there are also important clinical distinctions in the developmental and behavioral profile of these subgroups. Individuals with dup15q syndrome might have an initially preserved social smile and yet more marked neurological co-morbidities, including greater motor impairment.[10,11] The complexity of this genomic region, in addition with the long-standing challenge of studying the heterogeneity of brain tissue, has made it difficult to directly parse the effects of this variant in the human brain. In this study we sought to examine the transcriptional and epigenetic landscape of dup15q brain cell types compared to those of both neurotypical control individuals and individuals with non-dup15q ASD; we hypothesized that cell-type-specific effects, previously masked by brain-tissue heterogeneity, would reveal important biological distinctions in cellular pathophysiology and provide foundational information for future mechanistic and translational studies.

Material and methods

Brain tissue

Tissue was obtained from the NIH NeuroBioBank and Autism BrainNet with approval from their institutional review boards after

Table 1. Demographic information for samples

ID	Condition	Age	Sex	PMI	RIN	Seizure history
797	ASD	9	M	13	7.7	–
UMB1790	control	13	M	18	8.2	–
1793	control	11	M	19	–	–
UMB4643	control	42	F	4	8.2	–
5023	ASD	45	M	–	–	–
5278	ASD	15	F	13	6.9	yes
5391	control	8	M	19	6.9	–
5408	control	6	M	12	–	–
5497	control	68	M	24	8	–
7TJ5	ASD	13	M	26	6.7	–
862K	dup15q	12	M	30	5.4	yes
AN05983	dup15q	24	M	36	7.6	yes
AN06365	dup15q	10	M	18	8.1	yes
AN09412	ASD	29	M	38	7.3	–
AN11931	dup15q	39	F	33	8.5	yes
AN14067	ASD	38	M	46	8.7	yes
AN14762	dup15q	9	M	14	–	yes
PGEQ	control	16	M	23	6.8	–
UMB5565	ASD	12	M	22	–	–
Z2WU	dup15q	13	M	17	7.4	yes

PMI, postmortem interval. RIN, RNA integrity number.

written informed consent was obtained. Initial dissection of tissue for brain-bank specimens was done with sequential sectioning under standardized procedures. Research on these deidentified specimens and data was performed at Boston Children's Hospital with approval from the Committee on Clinical Investigation. Postmortem frozen frontal cortex was obtained from six affected individuals with dup15q, seven individuals with non-dup15q autism spectrum disorder (ASD), and seven neurotypical control individuals. 13 samples were processed for single-nucleus RNA sequencing (snRNA-seq), and seven for multi-omic sequencing (Figure 1C). All of the dup15q affected individuals were validated through copy-number analysis of the PWACR (tetrasomy)[6,7] and/or optical genome mapping (Figure S1). Methylation status in the brain has also been previously assessed in the majority of dup15q-affected individuals.[6,12] Groups were matched for age and sex (14%–17% female), and there were no significant differences between the RNA integrity number (RIN) and postmortem interval (PMI) between groups (Table 1; Figure S2). Frozen tissue was stored at −80°C and kept frozen until processing. ~25 mg pieces of tissue were dissected, with pre-chilled sterile forceps and scalpels, off the larger tissue block on a pre-chilled cryostat maintained at −20°C.

snRNA-sequencing and multi-omic sequencing

For nuclear isolation, tissue was subjected to glass Dounce homogenization followed by sucrose gradient centrifugation to isolate nuclei, as previously described.[13] Results presented here are from one technical replicate of each sample, but samples were run in subsets of matched groups over multiple days and sequencing runs so that batch effects would be minimized. For snRNA-seq, nuclei were resuspended in a nuclear resuspension buffer (1% BSA in PBS with 1 U/uL RNase inhibitor) and stained with Hoechst immediately prior to fluorescent-activated nuclear sorting on a BD Aria II, during which gating was used to remove debris, dying nuclei, and doublets. 10,000 nuclei/sample were sorted with a 100 μm low-pressure nozzle and immediately processed for 10× encapsulation as described below so that single-nucleus libraries could be generated. After encapsulation on the 10× Chromium controller, libraries were prepared as per the 10× Genomics Single Cell 3′ Kit. cDNA and library quality control were ensured through assessment of DNA on the Agilent Bioanalyzer High Sensitivity Chip.

For generation of multiome libraries, 50,000 nuclei were sorted as above directly into buffer (10 mM Tris-HCl [pH 7.4], 10 mM NaCl, 3 mM $MgCl_2$, 1% BSA, 1 mM DTT, Rnase inhibitor 1 U/uL). 1× Lysis Buffer (10 mM Tris-HCl, 10 mM NaCl, 3 mM $MgCl_2$, 0.1% Tween 20, 0.1% NP-40, 0.01% Digitonin, 1% BSA, 1 mM DTT, 1 U/uL Rnase inhibitor) was added to a final concentration of 0.1×, and the nuclei were incubated on ice for 2 min. Tween 20 was added (final concentration 0.1%), and nuclei were immediately pelleted by centrifugation at 500*g* × 5 min at 4°C in a bucket centrifuge. The pellet was resuspended in Diluted Nuclei Buffer (1× Nuclei Buffer (10× Genomics), 1 mM DTT, 1 U/uL RNase inhibitor) and centrifuged. The number of nuclei in the pellet was quantified with a hemocytometer before use of the 10× Genomics Chromium Next GEM Single Cell Multiome

ATAC + Gene Expression kit according to the manufacturer's instructions.

Libraries were sequenced with paired-end 150 bp reads on an Illumina NovaSeq 6000.

Bioinformatics analysis

For snRNA-seq analysis, the following steps were taken to process the samples: demultiplexing of raw data, alignment (to GRCh382020-A), quality control and filtering (see below), dimensionality reduction and unsupervised clustering. R (v.4.2.3), Cell Ranger (v.6.1.1.), and Seurat (v.4.3.0)[14] were used on the Boston Children's computing cluster E2. SoupX[15] was applied so that ambient RNAs would be removed, and scds[16] was used so that doublets and cells with extreme library sizes (out of the 95% confidence interval), an extreme number of features, and high content of mitochondrial reads (>10%) would be filtered out. We do not omit any sample based on the RIN given past work suggesting the RIN itself may be of more limited utility in the postmortem brain.[17–20] Quality control was performed over each independent sample, and ~50%–80% of nuclei remained after bioinformatic filtering, depending on the sample; there were equivalent metrics between conditions (Figure S3). Post-hoc analysis of known cell-type-specific and layer-specific markers were applied to the remaining nuclei for cluster identification and resolution optimization. One cluster representing a small percentage of nuclei (<1%) was manually removed given signatures of ambient RNA contamination as previously reported.[21] There is increased variability in snRNA-seq studies from human tissue. Thus, in addition to ensuring that there were no significant differences between age, sex, RIN, or PMI (Table 1; Figure S2), we also assessed the impact of various demographic factors on the variability of gene expression. We found minimal impact of age and sex via principal component analysis (Figure S2) and thus conducted differential-expression analysis with the FindMarkers functionality in Seurat by using default settings for the Wilcoxon rank-sum test, minimum log fold change > 0.2, and padj < 0.01 for all cell clusters. Our power calculations suggest that approximately 400 nuclei/condition are needed to detect 80% of gene expression changes with a false-discovery rate of 5%. We omitted downsampling to preserve power. For gene ontology (GO) enrichment, we used clusterProfiler R package.[22,23] A pathway is treated as enriched if an adjusted *p* values (with Benjamin-Hochberg correction) were smaller than 0.05. To identify changes in cellular composition, we used scCODA (single-cell compositional data analysis), a Bayesian hierarchical model developed for analyzing compositional data from single-cell RNA-sequencing studies.[24] It identifies cell types that are differentially abundant between conditions while accounting for the compositional nature of single-cell data.

We employed inferCNV (v.1.14.2),[25] a computational tool designed to infer copy-number variation (CNV) from single-cell RNA sequencing data, to investigate genomic instability across various conditions and cell types by using default parameters. We also utilized the R packages NGCHM (v.0.13.0) and infercnvNGCHM (v.0.1.1) for generating NGCHM files, thereby enhancing our inferCNV data interpretation and visualization with the NGCHM (Next-Generation Clustered HeatMap) interactive viewer.[26]

For multiome analysis, we mapped each sample to the human reference genome (CRCh38-2020-A-2.0.0) by using CellRanger Arc (v.2.0.0) with stringent snATAC cell filtering criteria, including counts per cell ranging from 1,000 to 1,000,000, nucleosome signal less than 2, and transcription start site (TSS) enrichment score larger than 1. Data preprocessing and normalization were conducted with Signac (v.1.9.0),[27] and sample integration was achieved through Harmony (v.0.1.1).[28] Cell-type annotations were informed by snRNAseq analysis results. We conducted motif enrichment analysis by using Signac; we added motif information to the Seurat object via the AddMotifs function and identified overrepresented motifs between various conditions by using FindMarkers. We computed motif activities per cell by using chromVAR (v.1.5.0).[29] For visualization, we generated multiple genomic plot types through Signac; we included accessibility tracks and gene annotations and leveraged the CoveragePlot() function for comprehensive genome-browser-style presentations. This integrative approach allowed for a nuanced exploration of genomic landscapes, highlighting differential accessibility and gene expression in various cell types.

To orthogonally assess the overlap between gene expression and ATAC-seq data, we implemented SCENIC (*s*ingle-*c*ell r*e*gulatory *n*etwork *i*nference and *c*lustering)[30] through its fast, python-based implementation (pySCENIC). Note that we could not directly compare the Signac and Seurat output with that of SCENIC because of differences in motif assignment. Thus, the associated transcription factors for all analyses were related and compared. We identified a statistically significant overlap in the dup15q vs. control comparison: our approach identified 382 predicted regulatory transcription factors, SCENIC identified 64, and there was an overlap of 22 (hypergeometric test, $p < 0.01$).

To generate a list of high-interest genes related to the duplicated region used for visualization and to focus our analysis, we utilized the GRCh38/hg38 assembly accessed through genome.ucsc.edu in conjunction with prior publications[1,6,7,31]; given individual differences in precise breakpoints, we also included genes beyond breakpoint 5 for comparative purposes.

Optical mapping

A custom protocol for human brain tissue was utilized (Bionano Genomics DN30400, Rev A). See supplemental methods for detailed protocol.

Results

After bioinformatic filtering, ~78,815 high-quality nuclei remained for the combined analysis (Figure 1D). Although there was individual variability in cell-type breakdown, each condition had comparable numbers of nuclei (Tables 2 and 3). Post-hoc annotation of cluster types from unsupervised clustering confirmed the expected distribution of layer-specific excitatory neurons, inhibitory neuron subtypes, glia, and other non-neuronal subtypes that have been previously described in cortical human tissue (Figure 1E).[13,32–34] No statistically significant changes in the composition of cell types between conditions was observed (Table 2; Figure 1F), consistent with independent reports.[35]

Using all nuclei in a pseudo-bulk tissue analysis demonstrated the expected upregulation of gene expression within the duplicated region, including *UBE3A*, in dup15q tissue compared to control tissue, consistent with prior published bulk RNA-seq work[7] (Figure 2A, Data S1; Table S1). We also identified more DEGs globally in

Table 2. Nuclei number per condition and totals

Cell type	dup15q/CNV	ASD	Control	Total
Astrocyte I	624	627	1,257	2,508
Astrocyte II	3,214	3,973	4,461	11,648
Endothelial	107	139	239	485
Inh-PVALB	158	103	113	374
Inh-PVALB II	431	416	473	1,320
Inh-SST	1,034	969	1,324	3,327
Inh-SV2C	699	454	683	1,836
Inh-VIP	871	475	776	2,122
Microglia	4,228	2,929	3,806	10,963
Neu L23	1,869	1,722	2,623	6,214
Neu L4	1,240	1,250	1,733	4,223
Neu L56	32	149	70	251
OL	110	120	84	314
Oligodendrocyte	5,409	5,869	10,888	22,166
OPC	3,662	2,916	3,650	10,228
Pericyte	165	196	313	674
T-cells	33	51	78	162
Total	23,886	22,358	32,571	78,815

Italics indicate cell types that are subsequently grouped in "glia" category, and all inhibitory (Inh) and excitatory (Neu) neurons are grouped as "neuron," for "low-resolution" analysis.

dup15q vs. control tissue, as compared to ASD vs. control or dup15q vs. ASD, suggesting unique biological perturbations in dup15q (Table S1).

We then conducted both high- and low-resolution expression analyses. First, for a "low-resolution" approach, we grouped broad neuronal subtypes (inhibitory and excitatory) and glial subtypes (oligodendrocyte lineage, microglia, and astrocytes). This approach maximized our power to detect expression changes within these categories.[36] Genome-wide, dup15q vs. control comparisons demonstrated the largest number of differentially expressed genes, in both neurons and glia, consistent with the broad analysis described above (Table S1). Compared to glia, neurons showed more marked increased expression across the duplicated region (Figure 2B). Genes within the PWACR region demonstrated preferential upregulation in neurons vs. glia in dup15q cases. (Data S1, Figure 2B). Not all genes demonstrated upregulation in both neurons and glia, although some, such as *UBE3A* and *HERC2*, did.

We also conducted "high-resolution" analysis that assessed changes in gene expression in more finely resolved individual cell types (e.g., layer 2/3 excitatory neurons and microglia). This analysis reinforced our findings above—namely, that cellular context is a critical parameter in gene expression changes, and cell-type-specific effects are observed even within neuron or glial subclasses (Data S1, Figure 3; Table S2). For example, the cluster of GABA receptor genes located within the PWACR demonstrated heterogeneity in gene expression changes between individual neuronal subtypes (Figure 3, Data S1). GABRB3 was upregulated in OPCs, and all inhibitory and excitatory neuron subtypes except for neurons in layers 5 and 6. GABRA5, on the other hand, was upregulated only in SV2C inhibitory neurons and excitatory neurons in layers 2–4. Thus, there was unexpected heterogeneity even across similar genes and cell types (Figure 3).

We took an orthogonal bioinformatic approach to confirm these cell-type-specific changes in gene expression in dup15q-affected individuals across the duplication. Bioinformatic tools, such as inferCNV, have been developed to infer somatic copy-number-variant status from single-cell transcriptomic studies in the cancer biology field, in which structural genomic alterations can confer cellular survival advantages and underlie clonal expansion.[25] We reasoned that although dup15q is a germline event, applying such tools to our data would reveal underlying heterogeneity in gene expression across the duplication. InferCNV applies a hidden Markov model to predict six states of gene dosage ranging from complete loss (state 1) to three or more copies (state 6) on the basis of single-cell RNA sequencing. As expected, applying inferCNV to all dup15q nuclei resulted in the prediction of a higher proportion of high-gene-dosage states in dup15q nuclei as compared to ASD nuclei (chi-square $p < 0 .001$). However, as expected, upregulation across the region of duplication was heterogeneous (Figure S5A). This again suggests that there

Table 3. Number of nuclei per sample

	Astrocyte I	Astrocyte II	Endothelial	Inh-PVALB	Inh-PVALB II	Inh-SST	Inh-SV2C	Inh-VIP	Microglia	Neu L23	Neu L4	Neu L56	OL	Oligodendrocyte	OPC	Pericyte	T cell
AN14762	114	659	4	16	19	89	54	84	901	124	172	2	24	460	998	5	2
AN06365	44	98	2	0	0	0	0	1	909	0	1	0	13	1614	489	2	16
AN05983	178	1410	35	23	32	198	86	126	268	151	40	4	12	927	631	36	6
AN11931	111	331	2	54	133	250	320	261	180	1001	240	4	0	149	145	6	0
ABN_862K	61	288	35	24	57	217	76	101	1,565	90	91	3	30	1395	309	42	6
ABN_ Z2WU	116	428	29	41	190	280	163	298	405	503	696	19	31	864	1,090	74	3
797	64	988	24	12	50	161	55	70	1,004	265	96	7	17	784	842	74	5
5278	184	980	36	1	9	13	27	4	862	35	41	0	40	1778	602	30	14
AN09412	17	58	9	26	63	105	64	37	1	245	209	45	2	29	19	4	3
AN14067	169	392	3	0	2	26	0	6	390	5	2	0	3	1,377	272	6	8
5023	138	1,006	1	19	36	254	98	131	159	430	64	2	3	772	211	12	6
ABN_7TJ5	15	76	40	27	149	183	113	82	337	555	388	48	13	498	461	41	11
UMB5565	40	473	26	18	107	227	97	145	176	187	450	47	42	631	509	29	4
5408	228	789	42	0	1	9	6	6	1,029	0	1	0	21	613	935	40	36
5391	99	676	18	27	118	323	116	111	581	1,518	899	26	7	1,012	412	18	10
1793	136	938	3	2	20	91	25	79	557	27	10	0	8	33	636	22	2
5497	308	988	58	6	23	157	39	56	549	28	9	0	0	1,672	241	111	2
UMB1790	44	188	70	54	245	359	322	299	290	908	681	36	16	1,082	706	61	0
ABN_ PGEQ	125	495	32	24	66	378	171	220	288	140	131	8	24	920	340	43	17
UMB4643	317	387	16	0	0	7	4	5	512	2	2	0	8	5,556	380	18	11
Total	2508	11,648	485	374	1,320	3,327	1,836	2,122	10,963	6,214	4,223	251	314	22,166	10,228	674	162

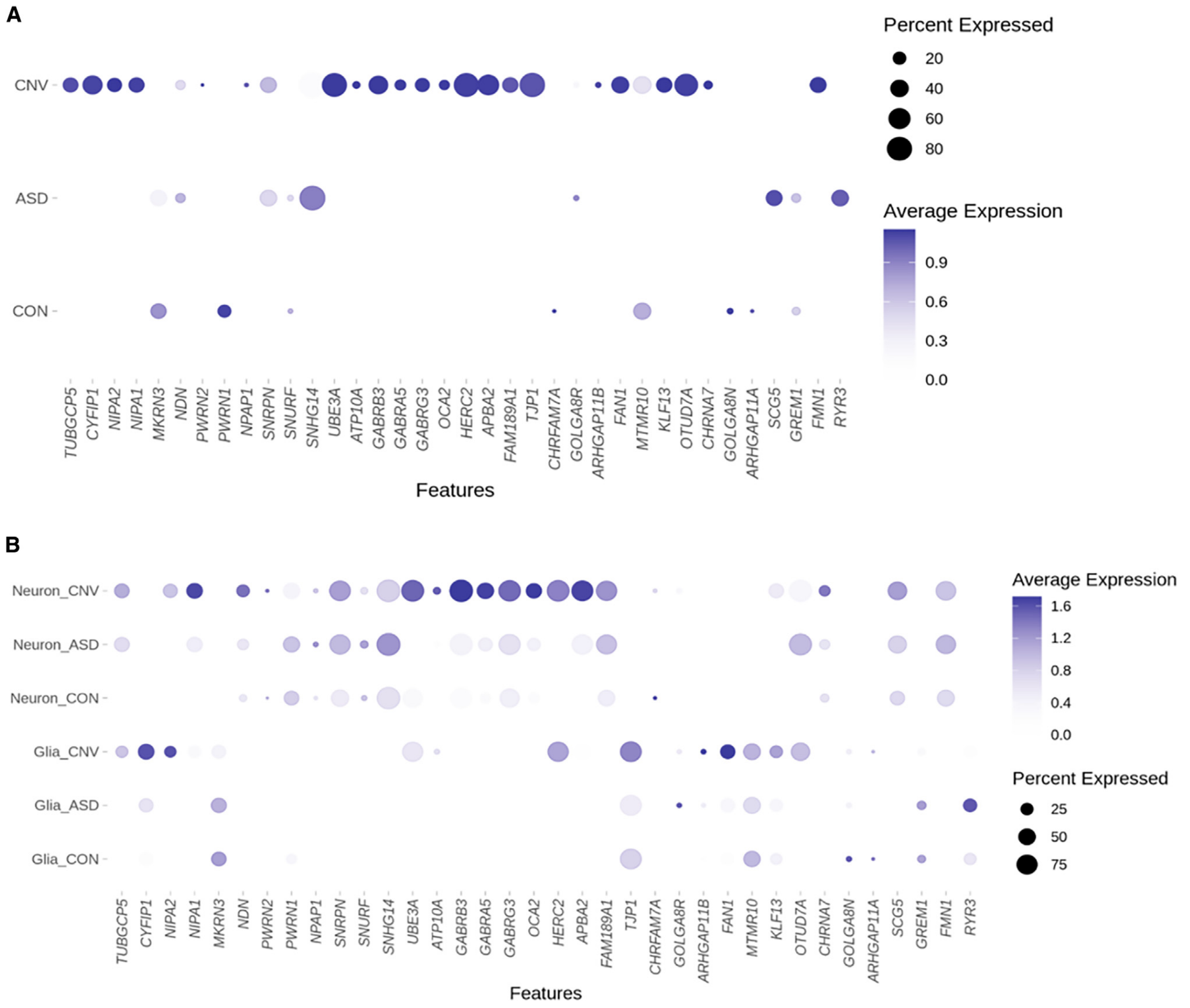

Figure 2. Expression changes within the proximal region of the long arm of chromosome 15 in ASD, dup15q (CNV), and control (CON) individuals

(A) Expected upregulation of the duplicated region when all nuclei are examined.

(B) Neuronal and glial nuclei broken down by condition demonstrate distinct patterns of changes in gene expression within this region; neurons demonstrate marked upregulation of the PWACR. The color heatmap is scaled average expression. Note that because visualization includes all cell types and scaled average expression is used, basal and ceiling effects might mask visualization of some changes between conditions. The list of significant differentially regulated genes can be found in Data S1.

is marked individual variability in dup15q changes and furthermore that not all cell types demonstrate "expected" changes within the duplicated region in dup15q in the human brain.

We next sought to identify potential mediating factors of gene expression changes. Differential gene expression studies typically focus on cells in which genes of interest are highly expressed at baseline, given the logic that those would be the cell types critical to understanding cellular pathophysiology; but copy-number gains create a situation where cell types with normally low expression may demonstrate greater fold changes in gene expression. We observed such a pattern with *CYFIP1* (MIM# 606322), located within the duplicated region. *CYFIP1* is normally highly expressed in microglia, and prior work has examined the role of microglial CYFIP1 in neurodevelopment.[37,38] In all nuclei taken together, *CYFIP1* mRNA was identified as significantly upregulated in dup15q (Data S1); this upregulation in expression was associated with increases in chromatin accessibility (Figure S4). However, these global changes were not observed in microglia, likely due to its high basal expression (Figure S4). Rather, cell types that demonstrated significant upregulation of *CYFIP1* were all neuronal, including inhibitory VIP and PVALB I and II neurons, and excitatory neurons in layers 2–4 (Data S1). Thus, our approach reveals that genes previously implicated in neurodevelopment can become misexpressed in non-canonical cell types in dup15q.

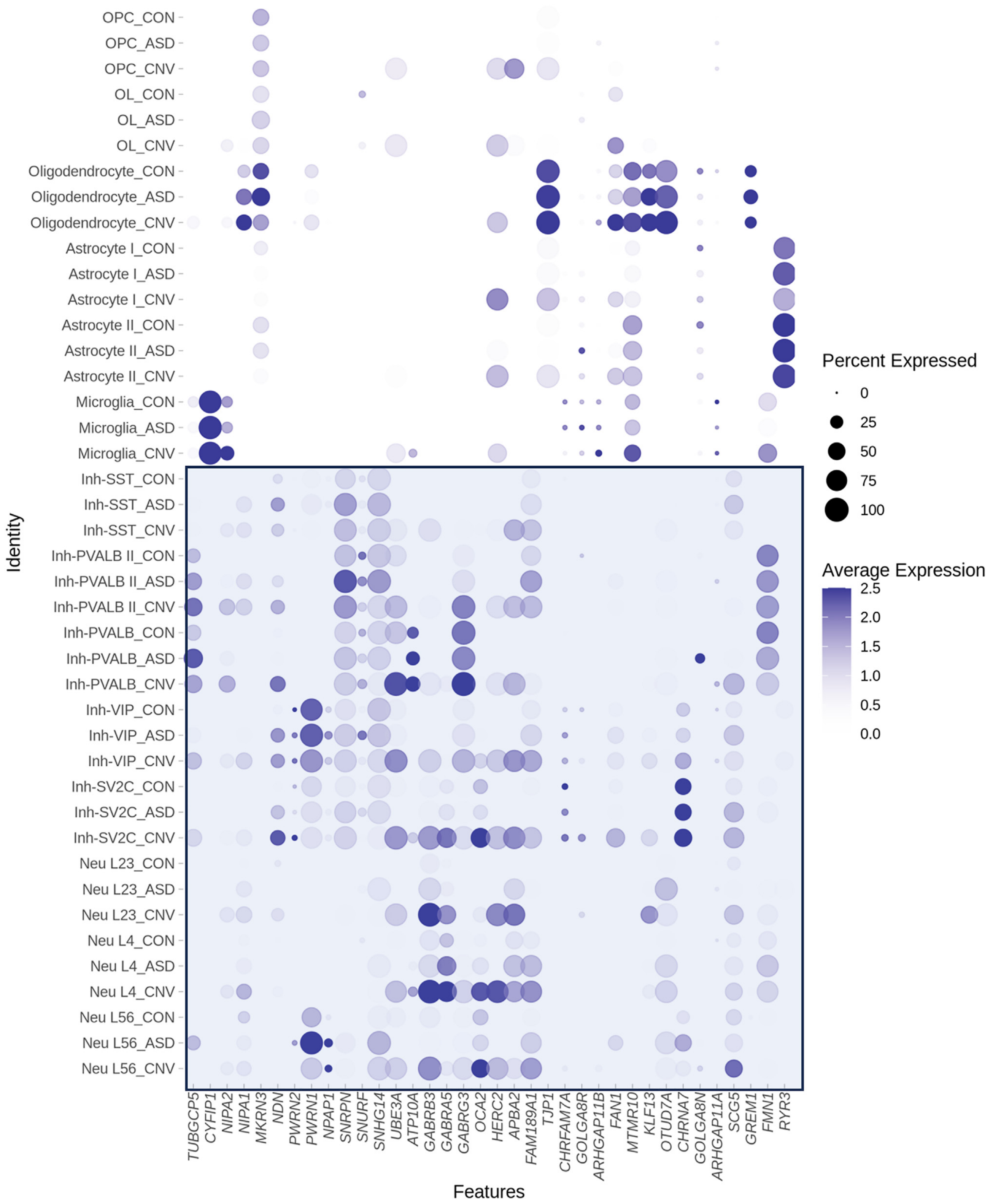

Figure 3. Dot plot depicting global cell-type-specific expression within the proximal region of chromosomal region 15q
The blue box highlights neuronal (bottom) subtypes. Note that because visualization includes all cell types, basal and ceiling effects might mask visualization of some changes between conditions. The list of significant differentially regulated genes can be found in Data S1.

To determine whether baseline expression could be mediating cell-type-specific effects on gene expression within the duplicated region, we examined the relationship between baseline expression in control nuclei and the change in expression in dup15q nuclei. There was no shift in distribution in the baseline expression of genes of

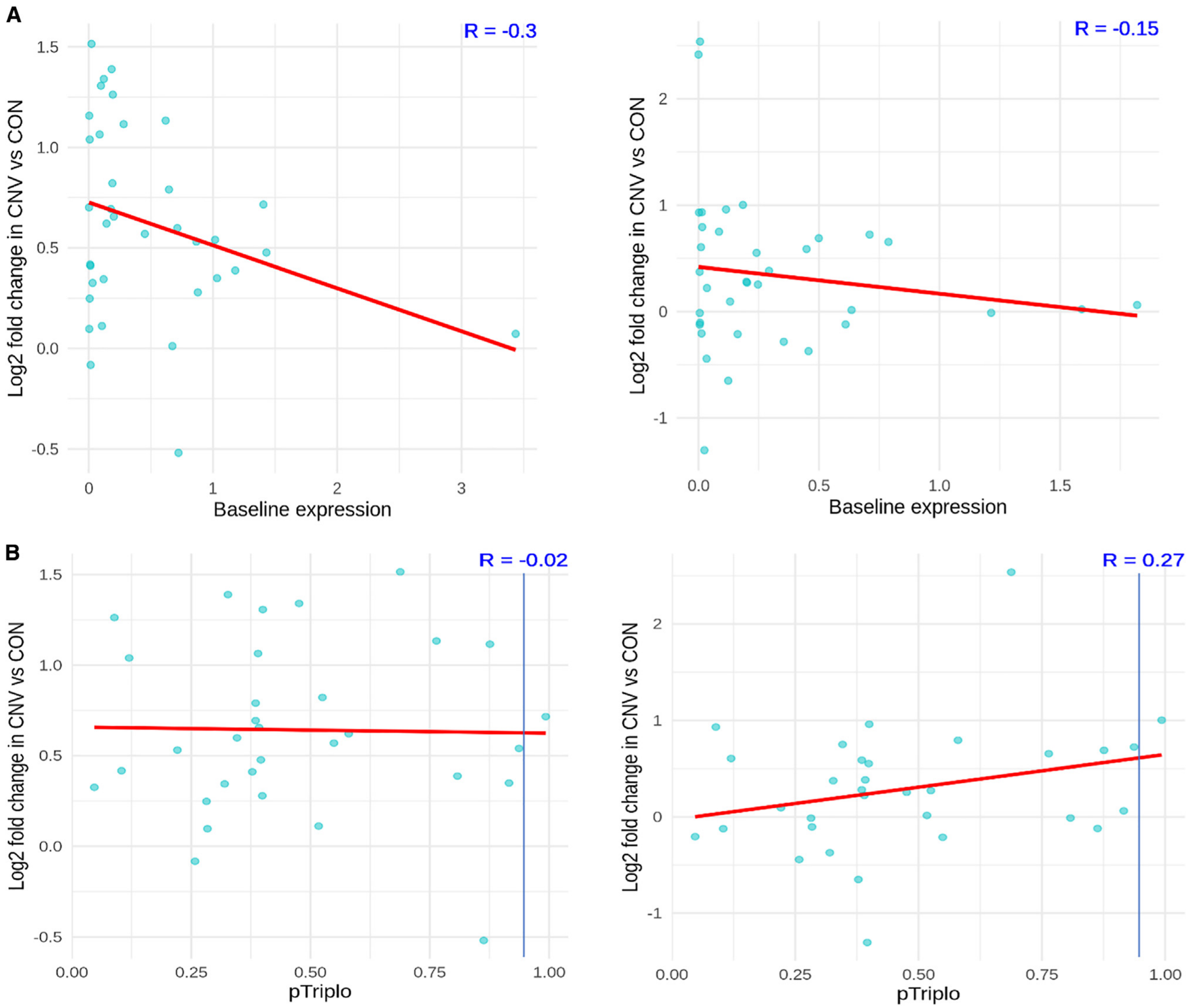

Figure 4. dup15q expression changes are not explained by baseline expression or genome-wide metrics
(A) Relationship of baseline expression of dup15q genes and relative change in neurons (left) vs. glia (right). Regardless of cell type, genes with high baseline expression show modest increases in dup15q-affected individuals.
(B) Relationship of pTriplo metric and relative change in dup15q genes in neurons (left) and glia (right). pTriplo > 0.94 is categorized as "triplosensitive." Note the dearth of dup15q genes meeting this criteria.

interest in the duplicated region in control nuclei in neurons versus glia. In dup15q nuclei, genes with the highest baseline expression demonstrated a modest change, a finding robust among different cell types. (Figure 4A) Thus, the neuronal-specific signature of increased expression was not due to neurons' having different baseline expression of the genes within this region.

Given that not all genes within a CNV are necessarily critical to the phenotype, we sought to clarify which genes would be both dysregulated and critical to the dup15q phenotype. To do this, we made use of recent advances in quantifying genome-wide dosage sensitivity to overexpression. Specifically, the "pTriplo" metric, or the probability of triplosensitivity, was recently developed from a cross-disorder catalog of rare CNVs that confer susceptibility to human disease; a number closer to one indicates a higher likelihood of being triplosensitive.[39] We hypothesized that many genes within the locus, particularly within the PWACR, previously deemed critical for the clinical phenotype, would demonstrate a high triplosensitive score and that a subset of those furthermore would show the highest relative change in expression in dup15q-affected individuals. Surprisingly, most genes of interest within the locus did not meet criteria for being triplosensitive as defined by the cutoff score of >0.94. (Figure S5). Furthermore, genes with a higher triplosensitivity metric again showed modest changes in gene expression, suggestive that homeostatic regulatory mechanisms are at play, regardless of cell type examined.

Carriers of dup15q have higher rates of epilepsy,[5,40] and in fact all of the dup15q-affected individuals analyzed here have a history of this co-morbidity. To determine whether

this might be influencing changes in gene expression within the region of the duplication, we separated out the (non-dup15q) ASD cases by the presence or absence of seizure diagnosis. We observed no global difference between the presence of an epilepsy or seizure diagnosis and its absence in non-dup15 ASD within our region of interest, and neither of these sub-groups resembled dup15q-affected individuals, arguing against the presence of seizures as a major confounding factor (Figure S6). We did observe notable heterogeneity from sample to sample, but no individual sample drove these findings, again suggesting that our results are robust and not due to individual sample differences in either demographic or technical factors (Figure S6; Table S3).

Our dataset also affords an opportunity to compare the analyses presented here to independent cell-type-specific reports on ASD. Encouragingly, we replicated past findings of neurodevelopmental disorders in our own gene expression data; these findings included enrichment of known pathogenic ASD risk genes, FMR1 protein target genes, and dysregulation of layer 2/3 excitatory neurons (Figures S7A and S7B).[7,9,32,35] Using gene ontology analysis, we also found evidence for biological processes involved in synaptic function in both excitatory and inhibitory subtypes in ASD and dup15q (Figure S7C).

We examined global changes in DNA accessibility by using snRNA-seq and snATAC-seq samples (i.e., multi-omic sequencing) (Figure 5A) and identifying differentially accessible genomic regions between conditions (Figure 5B, Data S1) and overrepresented motifs (Figure 5C, Data S1). Although gene-expression analysis showed a greater number of DEGs in dup15q individuals versus control individuals, as compared to non-dup15q ASD versus control individuals, we identified twice as many differentially accessible peaks in non-dup15q ASD versus control individuals (Figure 5B), consistent with the conclusion that dup15q primarily affects a specific genetic region. Additionally, there were almost four times as many differentially accessible peaks that were unique to either comparison, suggesting divergent patterns of genome-wide regulation in ASD and dup15q (Figure 5B).

Understanding the regulatory modules in ASD and dup15q is important for clarifying the underlying neurobiology. For example, our analyses suggested that specific genomic regions demonstrating differential accessibility are largely unique in ASD and dup15q but that there could be shared transcriptional regulatory modules as a result of convergent biological processes. We thus assessed enrichment for specific transcription-factor motifs in the differentially accessible genomic regions that were unique to each condition vs. those that were shared. Most motifs were not unique to any peak list (Figure 5C); these 256 motifs most likely represent shared developmentally critical hubs of transcriptional regulation. This is consistent with their activity enrichment across different cell types in our dataset (Figure 5D). Among the top motifs that were unique to ASD (69 total) were FOS- and JUN-related sites; genome-wide analysis demonstrated that these sites showed preferential activation in neuronal subtypes (Figure 5D). Examining the top enriched motifs unique to dup15q (49 total) demonstrated a distinct pattern—motifs critical to immune and inflammatory signaling, such as CEBPE, ELF4 and IRF4, were evident. In contrast to the top ASD-unique motifs that demonstrated enrichment of neuronal activity, our analyses highlighted microglial activity of these dup15q-unique motifs (Figure 5D). Furthermore, our snRNA-seq data showed that among non-neuronal subtypes, microglia contained the highest number of differentially expressed genes in dup15q (Table S2). Additionally, gene-ontology results for microglia in dup15q vs. control individuals revealed biological processes relevant to synaptic pruning, which was absent from the ASD vs. control comparison (Figure S8). Thus, we uncovered an unexpected inflammatory signature unique to dup15-affected individuals.

Finally, our approach of incorporating snATAC-seq afforded the opportunity to explore the relationship between gene expression and chromatin accessibility in more depth. In general, we found that, in all nuclei, any given gene within the dup15q locus demonstrated variable peak linkage, and the number of peaks associated with any given gene did not clearly correlate with pTriplo (Figure S9). However, when we explored the dup15q vs. control condition, we identified a significant positive association between differential chromatin accessibility and gene expression for dup15q genes and peaks in comparisons of neurons to glia (Figure S10). Thus, global chromatin reorganization is associated with the cell-type-specific observed changes in gene expression in dup15q.

Discussion

Here, we confirmed prior bulk RNA-seq work showing overall increased expression of genes within the region duplicated in dup15q brains[41] but further identified unexpected cell-type-specific heterogeneity in expression changes of genes within the duplicated region, specifically implicating dysregulation of the PWACR in neurons. Gene-expression changes demonstrated different patterns of regulation not fully explained by gene dosage, function, or even imprinting status. Other reports of dup15q-derived neurons demonstrated that cell type rather than genotype was the main determining factor in gene-expression changes, findings our work agrees with.[35,42] Unexpectedly, we found that the most highly expressed genes in any given cell type seemed to be spared from the most profound effects of increased gene dosage and that prior genome-wide metrics of dosage sensitivity did not necessarily capture gene-expression changes. Our work thus highlights the importance of unbiased and creative approaches in functionally validating the effects of copy-number variants directly in the tissue of interest in neurodevelopmental disorders.[43,44]

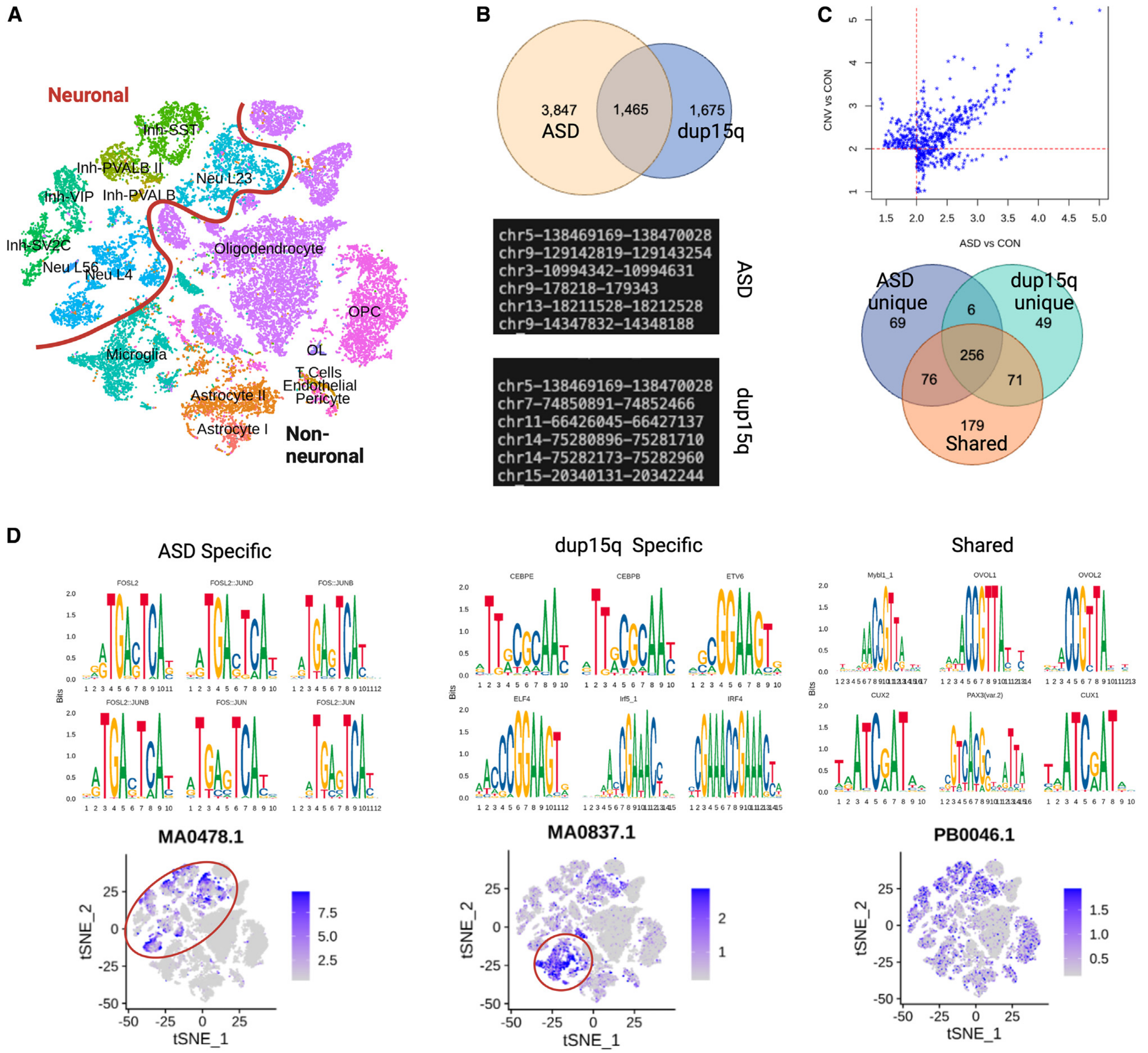

Figure 5. Multi-omic analysis reveals cell-type-specific transcriptional regulatory activity in dup15q-affected individuals
(A) Visualization of integration of snRNA-seq and snATAC-seq.
(B) Differential binding analysis revealed twice as many unique peaks in ASD vs. control nuclei as compared to dup15q nuclei vs. control. Examples of genomic locations for ASD and dup15q shown, note that dup15q contains loci both within and outside of the duplicated region.
(C) The identified peaks in (B) were used to assess relative enrichment of overrepresented motifs within differentially accessible genomic regions. The venn diagram demonstrates the overlap of significant (i.e., n-fold enrichment >2 and $p_{\text{adj}} < 0.05$) identified overrepresented motifs.
(D) FOS/JUN motifs were identified in the ASD-specific differentially accessible genomic regions. The per-cell-motif activity calculation revealed higher activity in neuronal subtypes (circled). Conversely, motifs involved in immune and inflammation signaling were enriched in peaks unique to dup15q, and corresponding microglial activity (circled) was observed. Motifs enriched from the "shared" group, (i.e., identified from differentially accessible sites present in both dup15q vs. control nuclei and ASD vs. control nuclei) lacked this cell-type specificity. Representative JASPAR matrix profiles MA0478.1 (FOSL2), MA.0837.1 (CEBPE), and PB0046.1 (Mybl1_1) are shown.

We also identified transcriptional regulatory networks implicating neuroinflammation and microglial function. However, microglial *CYFIP1*, a gene within the duplicated region, did not demonstrate evidence for significant regulation in dup15q-affected individuals, despite the fact that our study was well-powered to detect such changes in this cell type. Our work, which is observational in nature, cannot parse whether the microglial signal observed here represents a direct effect of the duplication or a response by the brain to dysfunction in other cell types. Future experimental work will be required to address this. This finding is nonetheless intriguing in light of the

emerging role of microglia in brain disorders across the lifespan.[38,45–47] We also resolved notable cell-type-specific changes in expression of the GABA receptor cluster within the duplicated region. Given the close genetic proximity of the three receptors, one might anticipate shared regulatory mechanisms, but we identified the most prominent dysregulation of GABRB3 in dup15q. This could be related to several factors, including regional differences that occur in the epigenetic landscape and are unique to frontal cortex or subtle parent-of-origin effects that persist despite biallelic expression.[48] Given findings that individuals with dup15q have an EEG biomarker mimicking increased GABAergic signaling, as well as recent clinical trials that target GABA transmission, this information could prove useful in guiding future precision therapeutics.[49,50]

Reassuringly, our work is concordant with past studies, which reinforces the biological plausibility of our unexpected findings of cell-type specificity of dup15q. For example, our multi-omic sequencing revealed enrichment of JUN and FOS motifs in differentially accessible regions in ASD but not in dup15q. This is consistent with existing literature implicating these networks in neurodevelopmental disorders, including ASD.[51,52] Replication of independent findings of enrichment of SFARI ASD risk genes and FMR1 protein networks, as well as layer 2/3 neuronal dysregulation in the pathogenesis of neurodevelopmental disorders, also indicated that our approach detected meaningful biological perturbations.[7,9,32,35,41] Indeed, results here confirmed independent laboratories' work using cellular models, as well as past experiments in human brain bulk tissue.[6,42]

Our data show that the effects of copy-number duplications are not straightforward. Given that dup15q is a germline event, it was unexpected that neurons demonstrated more marked dysregulation of gene expression within the duplicated region than glia. Although this implicates neuronal dysfunction in dup15q pathogenesis, it also provokes questions about what endogenous factors related to the epigenetic landscape moderate the effects of this germline structural variant. It is interesting that we found that cell types with high gene expression at baseline may conversely engage homeostatic mechanisms to minimize further increases, which could be potentially toxic to the cell. Better understanding of these differences could be used in the future to guide therapeutic strategies, for example through the harnessing of glial homeostatic mechanisms to tamp down effects of the duplication in neurons. Our unbiased, empiric identification of cell-type-specific changes in gene expression and the epigenetic landscape can also guide future therapeutic work. For example, noncanonical cell types might be critical in mediating the pathophysiology of dup15q and thus could be targeted in future precision-medicine therapeutic interventions, results that could easily be otherwise missed.

Limitations of this report include a small sample size of affected individuals. Thus, certain features, such as individual variability of dup15q or the effects of the duplication on very rare cell types, cannot be completely parsed from our methodological approach. However, our main findings, of unexpected cell-type-specific determinants of the effect of dup15q, are unlikely to be artifacts of our sample size. Our "low-resolution" analysis of glia and neurons corroborate the findings of the more subtle "high-resolution" approach. It is possible that the unique microglial inflammatory signature in dup15q is an artifact of the relatively high nuclei number that this cell type comprises. This is unlikely given that other cell types (astrocytes, for example) are present at similar or even higher proportions and were not implicated in multi-omic analysis in the same way. Future work will be needed to study whether the changes we identified are a direct result of alterations in the integrity of the imprinting center or more general genomic alterations. Additionally, we identified significant individual heterogeneity even within the dup15 cases, and this finding warrants future investigation into how genome-wide determinants might influence the effects of dup15, such as single-nucleotide polymorphisms. Nonetheless, as an important report of the cell-type-specific effects of copy-number gains in the human brain in neurodevelopmental disorders, our work reveals unexpected gene-expression changes that could fuel novel paths of investigation for therapeutic approaches. We also demonstrate the importance of direct, unbiased, and cell-type-specific studies in the human brain to complement mouse and *in vitro* cellular models.

Conclusions

We identified marked cell-type-specific regulation of gene expression and chromatin accessibility in human brain specimens from dup15q vs. non-dup15q ASD individuals and neurotypical control individuals, both within the region of the duplication and genome-wide. This work demonstrates that gene-expression changes caused by copy-number variants in neurodevelopmental disorders result from a complex interplay of factors as opposed to simply reflecting gene dosage. Neurons demonstrate marked evidence of altered gene expression in dup15q, particularly within the PWACR. We also identified evidence of an unexpected microglial inflammatory response unique to dup15q. This unexpected pattern of genomic regulation demonstrates the importance of directly studying the functional effects of germline genetic structural variants in the human brain in neurodevelopment, particularly in the context of copy-number gains that might behave unexpectedly. Our findings also demonstrate the importance of studying primary brain tissue directly with techniques that afford cell-type specificity and unbiased approaches. Our work will guide future mechanistic studies as well as rational therapeutic development in dup15q.

Data and code availability

Sequencing data are made available to the research community as per the informed consent of the donors and

their families. Data from the NIH NeuroBioBank is available through dbGAP (accession number phs000639v3p1). Data from Autism BrainNet is deposited in SFARI Base, a publicly available database accessible at https://www.sfari.org/resource/sfari-base/.

Supplemental information

Supplemental information can be found online at https://doi.org/10.1016/j.ajhg.2024.07.002.

Acknowledgments

We are grateful and indebted to the families who donated tissue for research purposes to Autism BrainNet, the ATP, and the NIH Neurobiobank at the University of Maryland, Baltimore, MD. The Autism BrainNet is a resource of the Simons Foundation Autism Research Initiative (SFARI). Autism BrainNet also manages the Autism Tissue Program (ATP) collection, previously funded by Autism Speaks. We thank Jennifer E. Neil for assistance with human samples; Robert Sean Hill, Dilenny Gonzalez, Shyam Akula, and Sattar Khoshkoo for assistance in reagent ordering and sample sequencing; Sara Bizzoto and Sattar Khoskkoo for discussion of cell-type-specific markers in cortex, Ronald Mathieu and the Hematology/Oncology Flow Cytometry Research Facility for assistance with sorting; the Engle lab and the Harvard Biopolymers Facility for assistance with Chromium Controller use and sequencing; and Emre Caglayan, Martin Breuss, and Tamim Shaikh for thoughtful comments on the manuscript. Support for quantification of the molecular genetics library was provided by the Boston Children's Hospital Intellectual and Developmental Disabilities Research Center Molecular Genetics Core Facility, supported by grant U54HD090255 from the NIH Eunice Kennedy Shriver National Institute of Child Health and Human Development. The bioinformatics analysis was performed with the computational resources provided by the Research Computing Group at Boston Children's Hospital and Harvard Medical School, including High-Performance Computing Clusters Enkefalos 2 (E2), and the BioGrids scientific software made available for data analysis. This work was supported by a grant from the Simons Foundation and Autism BrainNet (953759, C.A.W.), as well as NINDS (R01NS035129, C.A.W.). C.A.W. is also supported by the NIMH (U01MH106883) and the Tan Yang Center for Autism Research at Harvard Medical School. C.A.W. is an investigator of the Howard Hughes Medical Institute. C.M.D. is a Boettcher Webb-Waring early career investigator and was previously supported in part by the NIMH Translational Post-doctoral Training Program in Neurodevelopment (T32MH112510).

Author contributions

C.M.D. performed the snRNA-seq experiments, analyzed the data, and wrote the manuscript draft. A.M. conducted multi-omic sequencing experiments and analyzed the data. C.C., L.S., and S.R conducted bioinformatic analysis of transcriptomic and multi-omic sequencing. C.B. and K.C. conducted optical mapping experiments and analysis. C.A.W, C.M.D., and A.M. conceptualized the project, and C.A.W supervised the project. All authors reviewed and contributed to revising and editing the manuscript draft.

Declaration of interests

Authors have no competing interests to disclose.

Received: April 2, 2024
Accepted: July 2, 2024
Published: August 8, 2024

References

1. Wang, N.J., Parokonny, A.S., Thatcher, K.N., Driscoll, J., Malone, B.M., Dorrani, N., Sigman, M., LaSalle, J.M., and Schanen, N.C. (2008). Multiple forms of atypical rearrangements generating supernumerary derivative chromosome 15. BMC Genet. *9*, 2. https://doi.org/10.1186/1471-2156-9-2.
2. Hogart, A., Wu, D., LaSalle, J.M., and Schanen, N.C. (2010). The comorbidity of autism with the genomic disorders of chromosome 15q11.2-q13. Neurobiol. Dis. *38*, 181–191. https://doi.org/10.1016/j.nbd.2008.08.011.
3. Sanders, S.J., Ercan-Sencicek, A.G., Hus, V., Luo, R., Murtha, M.T., Moreno-De-Luca, D., Chu, S.H., Moreau, M.P., Gupta, A.R., Thomson, S.A., et al. (2011). Multiple Recurrent De Novo CNVs, Including Duplications of the 7q11.23 Williams Syndrome Region, Are Strongly Associated with Autism. Neuron *70*, 863–885. https://doi.org/10.1016/j.neuron.2011.05.002.
4. Marshall, C.R., Noor, A., Vincent, J.B., Lionel, A.C., Feuk, L., Skaug, J., Shago, M., Moessner, R., Pinto, D., Ren, Y., et al. (2008). Structural Variation of Chromosomes in Autism Spectrum Disorder. Am. J. Hum. Genet. *82*, 477–488. https://doi.org/10.1016/j.ajhg.2007.12.009.
5. Lusk, L., Vogel-Farley, V., DiStefano, C., and Jeste, S. (1993). Maternal 15q Duplication Syndrome. In GeneReviews((R)), M.P. Adam, J. Feldman, G.M. Mirzaa, R.A. Pagon, S.E. Wallace, L.J.H. Bean, K.W. Gripp, and A. Amemiya, eds.
6. Scoles, H.A., Urraca, N., Chadwick, S.W., Reiter, L.T., and LaSalle, J.M. (2011). Increased copy-number for methylated maternal 15q duplications leads to changes in gene and protein expression in human cortical samples. Mol. Autism. *2*, 19. https://doi.org/10.1186/2040-2392-2-19.
7. Tran, S.S., Jun, H.I., Bahn, J.H., Azghadi, A., Ramaswami, G., Van Nostrand, E.L., Nguyen, T.B., Hsiao, Y.E., Lee, C., Pratt, G.A., et al. (2019). Widespread RNA editing dysregulation in brains from autistic individuals. Nat. Neurosci. *22*, 25–36. https://doi.org/10.1038/s41593-018-0287-x.
8. Wexler, E.M., Rosen, E., Lu, D., Osborn, G.E., Martin, E., Raybould, H., and Geschwind, D.H. (2011). Genome-wide analysis of a Wnt1-regulated transcriptional network implicates neurodegenerative pathways. Sci. Signal. *4*, ra65. https://doi.org/10.1126/scisignal.2002282.
9. Nishimura, Y., Martin, C.L., Vazquez-Lopez, A., Spence, S.J., Alvarez-Retuerto, A.I., Sigman, M., Steindler, C., Pellegrini, S., Schanen, N.C., Warren, S.T., and Geschwind, D.H. (2007). Genome-wide expression profiling of lymphoblastoid cell lines distinguishes different forms of autism and reveals shared pathways. Hum. Mol. Genet. *16*, 1682–1698. https://doi.org/10.1093/hmg/ddm116.
10. Simon, E.W., Haas-Givler, B., and Finucane, B. (2010). A longitudinal follow-up study of autistic symptoms in children and adults with duplications of 15q11-13. Am. J. Med. Genet. B Neuropsychiatr. Genet. *153B*, 463–467. https://doi.org/10.1002/ajmg.b.31000.

11. DiStefano, C., Gulsrud, A., Huberty, S., Kasari, C., Cook, E., Reiter, L.T., Thibert, R., and Jeste, S.S. (2016). Identification of a distinct developmental and behavioral profile in children with Dup15q syndrome. J. Neurodev. Disord. *8*, 19. https://doi.org/10.1186/s11689-016-9152-y.
12. Wong, C.C.Y., Smith, R.G., Hannon, E., Ramaswami, G., Parikshak, N.N., Assary, E., Troakes, C., Poschmann, J., Schalkwyk, L.C., Sun, W., et al. (2019). Genome-wide DNA methylation profiling identifies convergent molecular signatures associated with idiopathic and syndromic autism in post-mortem human brain tissue. Hum. Mol. Genet. *28*, 2201–2211. https://doi.org/10.1093/hmg/ddz052.
13. Dias, C.M., Issac, B., Sun, L., Lukowicz, A., Talukdar, M., Akula, S.K., Miller, M.B., Walsh, K., Rockowitz, S., and Walsh, C.A. (2023). Glial dysregulation in the human brain in fragile X-associated tremor/ataxia syndrome. Proc. Natl. Acad. Sci. USA *120*, e2300052120. https://doi.org/10.1073/pnas.2300052120.
14. Satija, R., Farrell, J.A., Gennert, D., Schier, A.F., and Regev, A. (2015). Spatial reconstruction of single-cell gene expression data. Nat. Biotechnol. *33*, 495–502. https://doi.org/10.1038/nbt.3192.
15. Young, M.D., and Behjati, S. (2020). SoupX removes ambient RNA contamination from droplet-based single-cell RNA sequencing data. GigaScience *9*, giaa151. https://doi.org/10.1093/gigascience/giaa151.
16. Bais, A.S., and Kostka, D. (2020). scds: computational annotation of doublets in single-cell RNA sequencing data. Bioinformatics *36*, 1150–1158. https://doi.org/10.1093/bioinformatics/btz698.
17. White, K., Yang, P., Li, L., Farshori, A., Medina, A.E., and Zielke, H.R. (2018). Effect of Postmortem Interval and Years in Storage on RNA Quality of Tissue at a Repository of the NIH NeuroBioBank. Biopreserv. Biobank. *16*, 148–157. https://doi.org/10.1089/bio.2017.0099.
18. Sonntag, K.C., Tejada, G., Subburaju, S., Berretta, S., Benes, F.M., and Woo, T.U. (2016). Limited predictability of postmortem human brain tissue quality by RNA integrity numbers. J. Neurochem. *138*, 53–59. https://doi.org/10.1111/jnc.13637.
19. Nagy, C., Maheu, M., Lopez, J.P., Vaillancourt, K., Cruceanu, C., Gross, J.A., Arnovitz, M., Mechawar, N., and Turecki, G. (2015). Effects of Postmortem Interval on Biomolecule Integrity in the Brain. J. Neuropathol. Exp. Neurol. *74*, 459–469. https://doi.org/10.1097/NEN.0000000000000190.
20. Durrenberger, P.F., Fernando, S., Kashefi, S.N., Ferrer, I., Hauw, J.J., Seilhean, D., Smith, C., Walker, R., Al-Sarraj, S., Troakes, C., et al. (2010). Effects of antemortem and postmortem variables on human brain mRNA quality: a BrainNet Europe study. J. Neuropathol. Exp. Neurol. *69*, 70–81. https://doi.org/10.1097/NEN.0b013e3181c7e32f.
21. Caglayan, E., Liu, Y., and Konopka, G. (2022). Neuronal ambient RNA contamination causes misinterpreted and masked cell types in brain single-nuclei datasets. Neuron *110*, 4043–4056.e5. https://doi.org/10.1016/j.neuron.2022.09.010.
22. Yu, G., Wang, L.G., Han, Y., and He, Q.Y. (2012). clusterProfiler: an R package for comparing biological themes among gene clusters. OMICS *16*, 284–287. https://doi.org/10.1089/omi.2011.0118.
23. Wu, T., Hu, E., Xu, S., Chen, M., Guo, P., Dai, Z., Feng, T., Zhou, L., Tang, W., Zhan, L., et al. (2021). clusterProfiler 4.0: A universal enrichment tool for interpreting omics data. Innovation *2*, 100141. https://doi.org/10.1016/j.xinn.2021.100141.
24. Buttner, M., Ostner, J., Muller, C.L., Theis, F.J., and Schubert, B. (2021). scCODA is a Bayesian model for compositional single-cell data analysis. Nat. Commun. *12*, 6876. https://doi.org/10.1038/s41467-021-27150-6.
25. Patel, A.P., Tirosh, I., Trombetta, J.J., Shalek, A.K., Gillespie, S.M., Wakimoto, H., Cahill, D.P., Nahed, B.V., Curry, W.T., Martuza, R.L., et al. (2014). Single-cell RNA-seq highlights intratumoral heterogeneity in primary glioblastoma. Science *344*, 1396–1401. https://doi.org/10.1126/science.1254257.
26. Ryan, M.C., Stucky, M., Wakefield, C., Melott, J.M., Akbani, R., Weinstein, J.N., and Broom, B.M. (2019). Interactive Clustered Heat Map Builder: An easy web-based tool for creating sophisticated clustered heat maps. F1000Res. *8*. ISCB Comm J-1750. https://doi.org/10.12688/f1000research.20590.2.
27. Stuart, T., Srivastava, A., Madad, S., Lareau, C.A., and Satija, R. (2021). Single-cell chromatin state analysis with Signac. Nat. Methods *18*, 1333–1341. https://doi.org/10.1038/s41592-021-01282-5.
28. Korsunsky, I., Millard, N., Fan, J., Slowikowski, K., Zhang, F., Wei, K., Baglaenko, Y., Brenner, M., Loh, P.R., and Raychaudhuri, S. (2019). Fast, sensitive and accurate integration of single-cell data with Harmony. Nat. Methods *16*, 1289–1296. https://doi.org/10.1038/s41592-019-0619-0.
29. Schep, A.N., Wu, B., Buenrostro, J.D., and Greenleaf, W.J. (2017). chromVAR: inferring transcription-factor-associated accessibility from single-cell epigenomic data. Nat. Methods *14*, 975–978. https://doi.org/10.1038/nmeth.4401.
30. Aibar, S., Gonzalez-Blas, C.B., Moerman, T., Huynh-Thu, V.A., Imrichova, H., Hulselmans, G., Rambow, F., Marine, J.C., Geurts, P., Aerts, J., et al. (2017). SCENIC: single-cell regulatory network inference and clustering. Nat. Methods *14*, 1083–1086. https://doi.org/10.1038/nmeth.4463.
31. Battaglia, A. (2008). The inv dup (15) or idic (15) syndrome (Tetrasomy 15q). Orphanet J. Rare Dis. *3*, 30. https://doi.org/10.1186/1750-1172-3-30.
32. Velmeshev, D., Schirmer, L., Jung, D., Haeussler, M., Perez, Y., Mayer, S., Bhaduri, A., Goyal, N., Rowitch, D.H., and Kriegstein, A.R. (2019). Single-cell genomics identifies cell type-specific molecular changes in autism. Science *364*, 685–689. https://doi.org/10.1126/science.aav8130.
33. Nagy, C., Maitra, M., Tanti, A., Suderman, M., Theroux, J.F., Davoli, M.A., Perlman, K., Yerko, V., Wang, Y.C., Tripathy, S.J., et al. (2020). Single-nucleus transcriptomics of the prefrontal cortex in major depressive disorder implicates oligodendrocyte precursor cells and excitatory neurons. Nat. Neurosci. *23*, 771–781. https://doi.org/10.1038/s41593-020-0621-y.
34. Langseth, C.M., Gyllborg, D., Miller, J.A., Close, J.L., Long, B., Lein, E.S., Hilscher, M.M., and Nilsson, M. (2021). Comprehensive in situ mapping of human cortical transcriptomic cell types. Commun. Biol. *4*, 998. https://doi.org/10.1038/s42003-021-02517-z.
35. Perez, Y., Velmeshev, D., Wang, L., White, M., Siebert, C., Baltazar, J., Dutton, N.G., Wang, S., Haeussler, M., Chamberlain, S., and Kriegstein, A. (2023). Single cell analysis of dup15q syndrome reveals developmental and postnatal molecular changes in autism. Preprint at bioRxiv. https://doi.org/10.1101/2023.09.22.559056.
36. Maitra, M., Mitsuhashi, H., Rahimian, R., Chawla, A., Yang, J., Fiori, L.M., Davoli, M.A., Perlman, K., Aouabed, Z., Mash,

D.C., et al. (2023). Cell type specific transcriptomic differences in depression show similar patterns between males and females but implicate distinct cell types and genes. Nat. Commun. *14*, 2912. https://doi.org/10.1038/s41467-023-38530-5.

37. Sheridan, S.D., Horng, J.E., Yeh, H., McCrea, L., Wang, J., Fu, T., and Perlis, R.H. (2024). Loss of Function in the Neurodevelopmental Disease and Schizophrenia-Associated Gene CYFIP1 in Human Microglia-like Cells Supports a Functional Role in Synaptic Engulfment. Biol. Psychiatry *95*, 676–686. https://doi.org/10.1016/j.biopsych.2023.07.022.
38. Haan, N., Westacott, L.J., Carter, J., Owen, M.J., Gray, W.P., Hall, J., and Wilkinson, L.S. (2021). Haploinsufficiency of the schizophrenia and autism risk gene Cyfip1 causes abnormal postnatal hippocampal neurogenesis through microglial and Arp2/3 mediated actin dependent mechanisms. Transl. Psychiatry *11*, 313. https://doi.org/10.1038/s41398-021-01415-6.
39. Collins, R.L., Glessner, J.T., Porcu, E., Lepamets, M., Brandon, R., Lauricella, C., Han, L., Morley, T., Niestroj, L.M., Ulirsch, J., et al. (2022). A cross-disorder dosage sensitivity map of the human genome. Cell *185*, 3041–3055.e25. https://doi.org/10.1016/j.cell.2022.06.036.
40. Conant, K.D., Finucane, B., Cleary, N., Martin, A., Muss, C., Delany, M., Murphy, E.K., Rabe, O., Luchsinger, K., Spence, S.J., et al. (2014). A survey of seizures and current treatments in 15q duplication syndrome. Epilepsia 55, 396–402.
41. Parikshak, N.N., Swarup, V., Belgard, T.G., Irimia, M., Ramaswami, G., Gandal, M.J., Hartl, C., Leppa, V., Ubieta, L.D.L.T., Huang, J., et al. (2016). Genome-wide changes in lncRNA, splicing, and regional gene expression patterns in autism. Nature *540*, 423–427. https://doi.org/10.1038/nature20612.
42. Germain, N.D., Chen, P.F., Plocik, A.M., Glatt-Deeley, H., Brown, J., Fink, J.J., Bolduc, K.A., Robinson, T.M., Levine, E.S., Reiter, L.T., et al. (2014). Gene expression analysis of human induced pluripotent stem cell-derived neurons carrying copy-number variants of chromosome 15q11-q13.1. Mol. Autism. *5*, 44. https://doi.org/10.1186/2040-2392-5-44.
43. Liu, X., Xu, W., Leng, F., Zhang, P., Guo, R., Zhang, Y., Hao, C., Ni, X., and Li, W. (2023). NeuroCNVscore: a tissue-specific framework to prioritise the pathogenicity of CNVs in neurodevelopmental disorders. BMJ Paediatr. Open *7*, e001966. https://doi.org/10.1136/bmjpo-2023-001966.
44. Sun, J., Noss, S., Banerjee, D., Das, M., and Girirajan, S. (2024). Strategies for dissecting the complexity of neurodevelopmental disorders. Trends Genet. *40*, 187–202. https://doi.org/10.1016/j.tig.2023.10.009.
45. Tian, Y., Xiao, X., Liu, W., Cheng, S., Qian, N., Wang, L., Liu, Y., Ai, R., and Zhu, X. (2024). TREM2 improves microglia function and synaptic development in autism spectrum disorders by regulating P38 MAPK signaling pathway. Mol. Brain *17*, 12. https://doi.org/10.1186/s13041-024-01081-x.
46. Prater, K.E., Green, K.J., Mamde, S., Sun, W., Cochoit, A., Smith, C.L., Chiou, K.L., Heath, L., Rose, S.E., Wiley, J., et al. (2023). Human microglia show unique transcriptional changes in Alzheimer's disease. Nat. Aging *3*, 894–907. https://doi.org/10.1038/s43587-023-00424-y.
47. Martinez Cerdeno, V., Hong, T., Amina, S., Lechpammer, M., Ariza, J., Tassone, F., Noctor, S.C., Hagerman, P., and Hagerman, R. (2018). Microglial cell activation and senescence are characteristic of the pathology FXTAS. Mov. Disord. *33*, 1887–1894. https://doi.org/10.1002/mds.27553.
48. Hogart, A., Nagarajan, R.P., Patzel, K.A., Yasui, D.H., and Lasalle, J.M. (2007). 15q11-13 GABAA receptor genes are normally biallelically expressed in brain yet are subject to epigenetic dysregulation in autism-spectrum disorders. Hum. Mol. Genet. *16*, 691–703. https://doi.org/10.1093/hmg/ddm014.
49. Frohlich, J., Reiter, L.T., Saravanapandian, V., DiStefano, C., Huberty, S., Hyde, C., Chamberlain, S., Bearden, C.E., Golshani, P., Irimia, A., et al. (2019). Mechanisms underlying the EEG biomarker in Dup15q syndrome. Mol. Autism. *10*, 29. https://doi.org/10.1186/s13229-019-0280-6.
50. Saravanapandian, V., Nadkarni, D., Hsu, S.H., Hussain, S.A., Maski, K., Golshani, P., Colwell, C.S., Balasubramanian, S., Dixon, A., Geschwind, D.H., and Jeste, S.S. (2021). Abnormal sleep physiology in children with 15q11.2-13.1 duplication (Dup15q) syndrome. Mol. Autism. *12*, 54. https://doi.org/10.1186/s13229-021-00460-8.
51. Wenderski, W., Wang, L., Krokhotin, A., Walsh, J.J., Li, H., Shoji, H., Ghosh, S., George, R.D., Miller, E.L., Elias, L., et al. (2020). Loss of the neural-specific BAF subunit ACTL6B relieves repression of early response genes and causes recessive autism. Proc. Natl. Acad. Sci. USA *117*, 10055–10066. https://doi.org/10.1073/pnas.1908238117.
52. Li, H., Wang, X., Hu, C., Li, H., Xu, Z., Lei, P., Luo, X., and Hao, Y. (2022). JUN and PDGFRA as Crucial Candidate Genes for Childhood Autism Spectrum Disorder. Front. Neuroinform. *16*, 800079. https://doi.org/10.3389/fninf.2022.800079.

The American Journal of Human Genetics, Volume *111*

Supplemental information

Cell-type-specific effects

# of autism-associated 15q duplication

syndrome in the human brain

Caroline Dias, Alisa Mo, Chunhui Cai, Liang Sun, Kristen Cabral, Catherine A. Brownstein, Shira Rockowitz, and Christopher A. Walsh

Supplemental Figures

A

	Prior Validation
**862K**	blood karyotype and FISH
**AN05983**	blood genotyping, FISH, southern blot, array CGH; brain WGS, RT-PCR of genomic DNA, RNA-seq
**AN06365**	blood genotyping, FISH, southern blot, array CGH; brain WGS, RT-PCR of genomic DNA, RNA-seq, RT-PCR
**AN11931**	blood genotyping, FISH, southern blot, array CGH; brain RT-PCR of genomic DNA, RNA-seq, RT-PCR
**AN14762**	blood genotyping, FISH, southern blot, array CGH; brain RT-PCR of genomic DNA, RNA-seq
**Z2WU**	blood karyotype and FISH

B

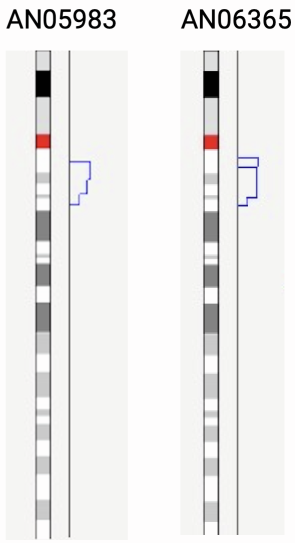

Figure S1: Dup15q sample validation. (All samples have tetrasomy of the PWACR) **A.** Dup15q samples have been extensively validated in the literature[6,41]. **B.** Two samples were also validated with optical genome mapping. Note that the ideogram represents only the copy number changes and not the chromosomal structure.

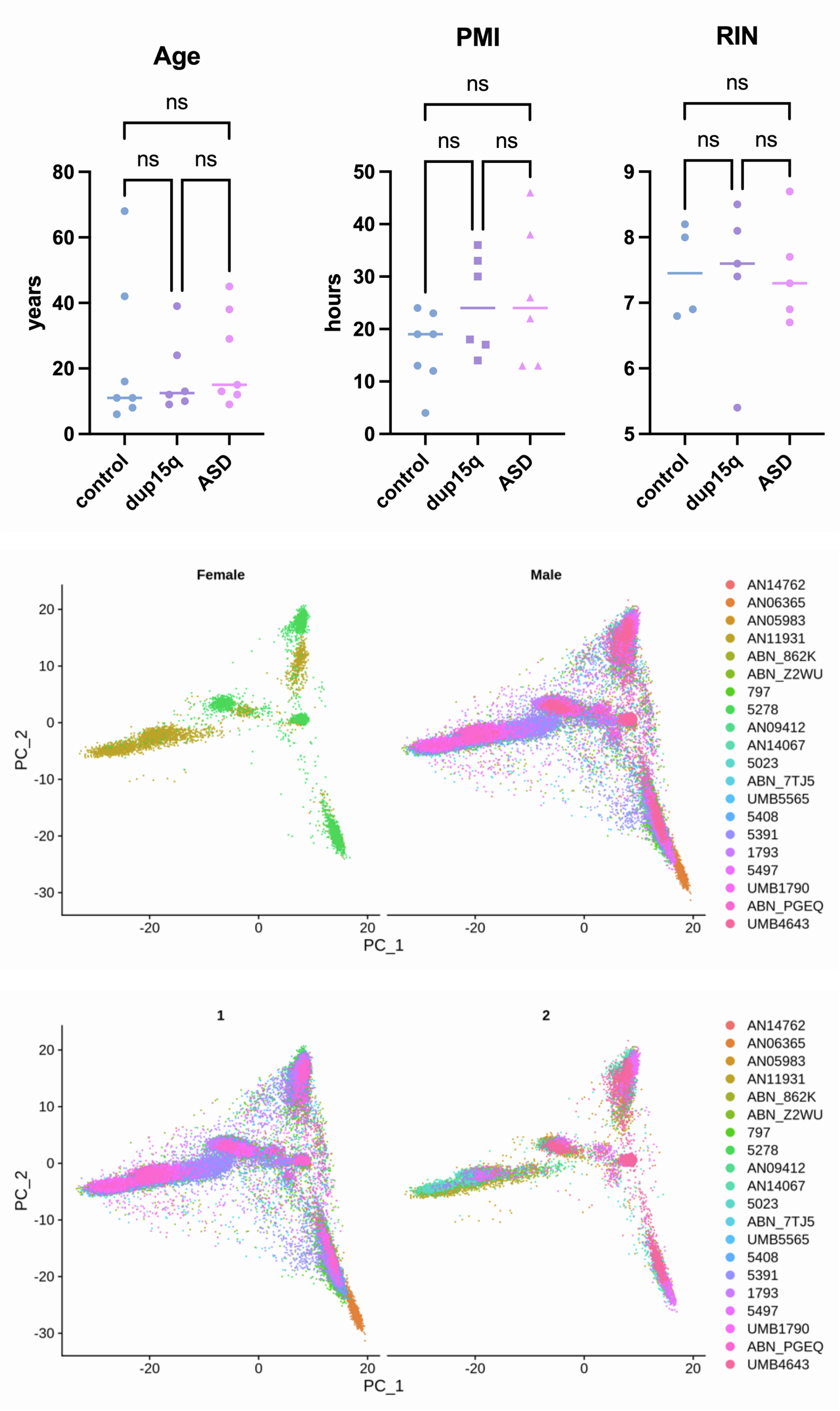

Figure S2: Impact of demographics. Top panel: Sample comparison. No significant differences in age, PMI or RIN. ($p > .05$, 1 way ANOVA, Tukey post-hoc test. There was also no difference in the female percentage between groups ($p > .05$, chi-square). Middle and bottom panel: PCA plots demonstrate sex and age (split as < and > 21 years of age) are not major determinants of gene expression variability.

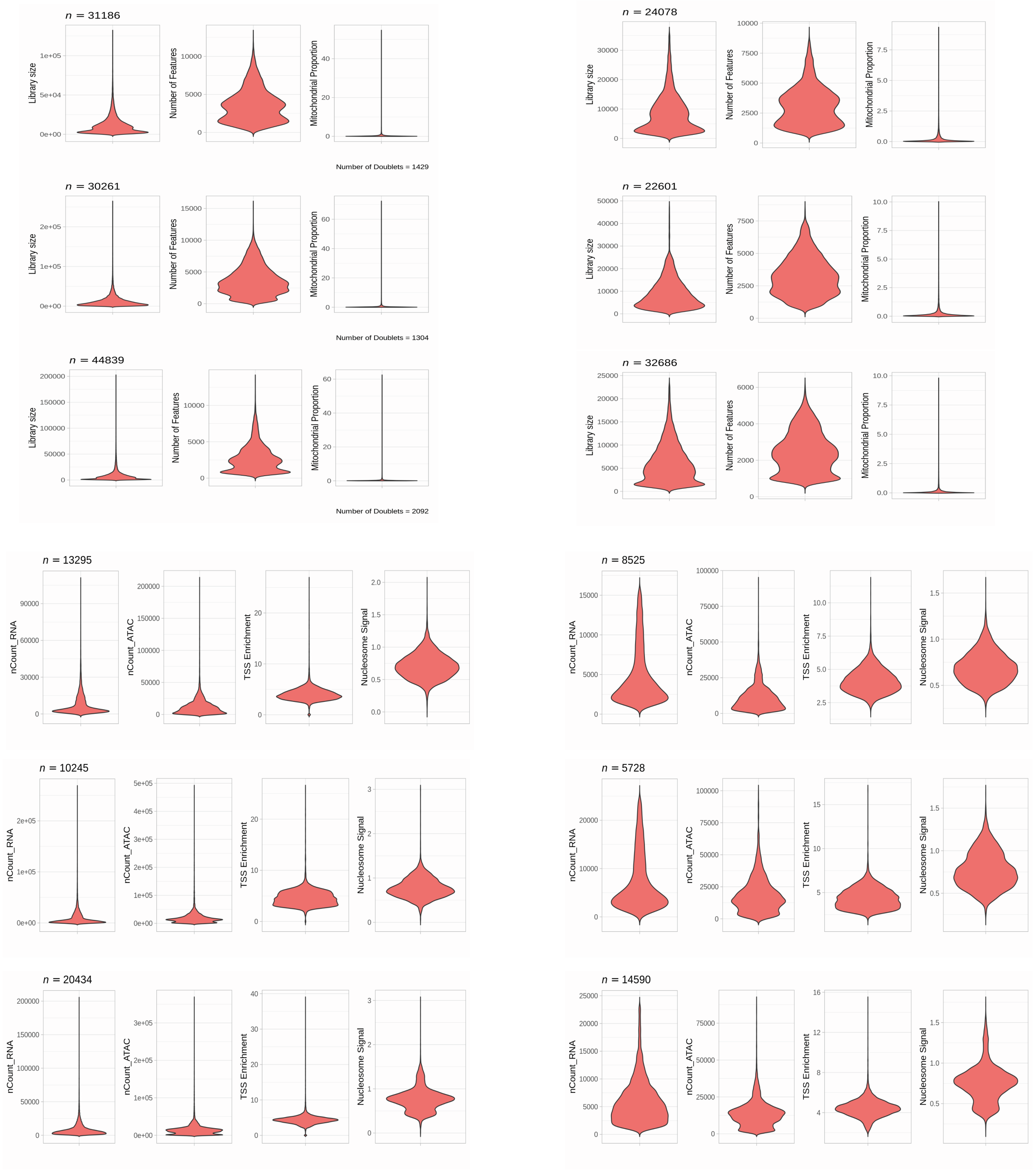

Figure S3: Quality control and filtering. snRNA-seq (top 3) and multi-omic (bottom 3) quality metrics of raw (left) and filtered (right) nuclei. Each panel demonstrates library size, gene number, and proportion of mitochondrial genes (from left to right) and for ATAC-seq- ncount RNA, ATAC, TSS enrichment and nucleosome signal are shown. Top is dup15q, middle is ASD, and bottom is control. Number of nuclei in top left corner, number of doublets in each group prior to filtering also indicated.

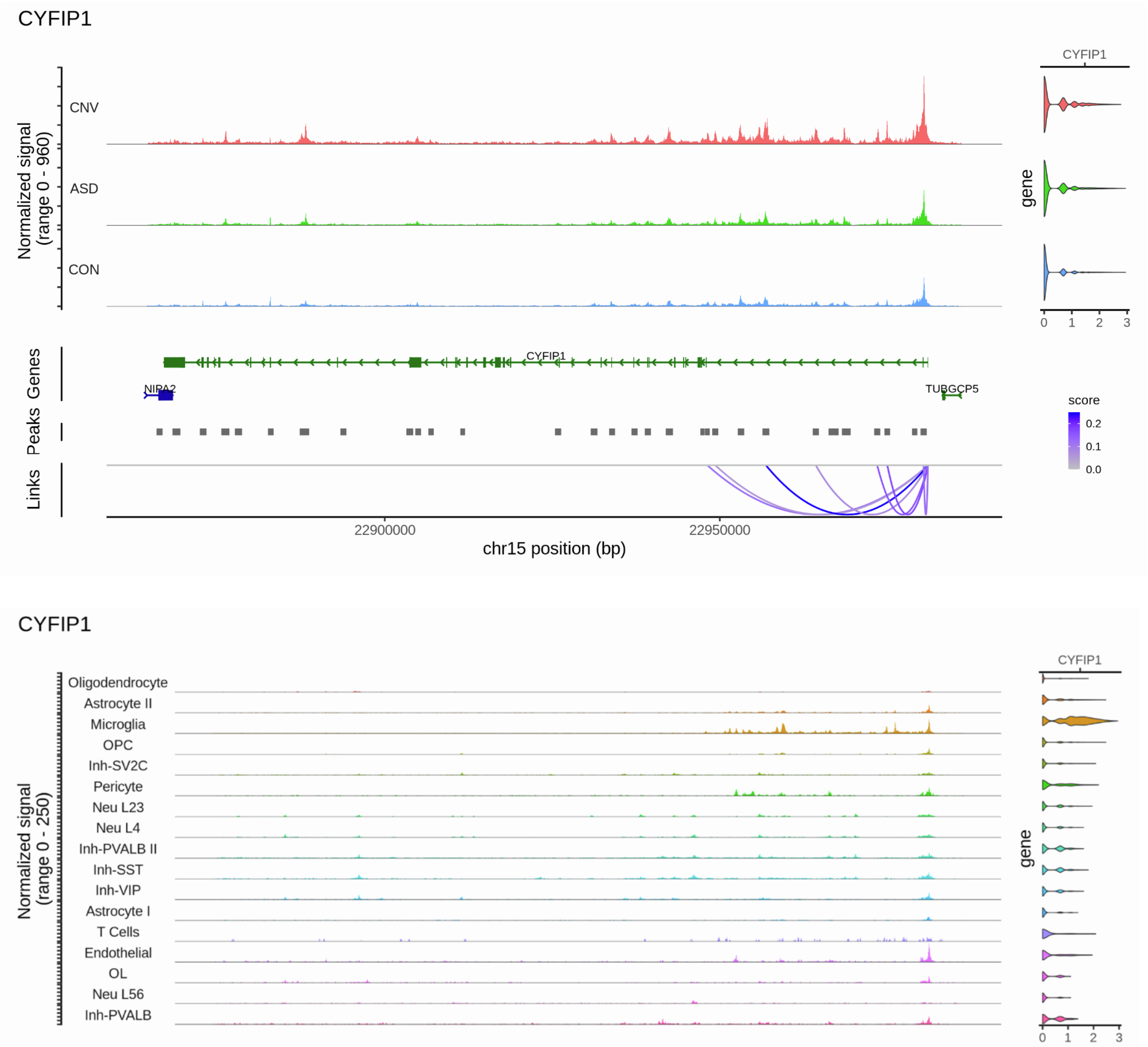

Figure S4: *CYFIP1* chromatin accessibility. Increased expression and chromatin accessibility at *CYFIP1* in dup15q samples shown, as well as peak to peak linkage and cell-type-specific chromatin accessibility. Top panels represent signal from all nuclei combined.

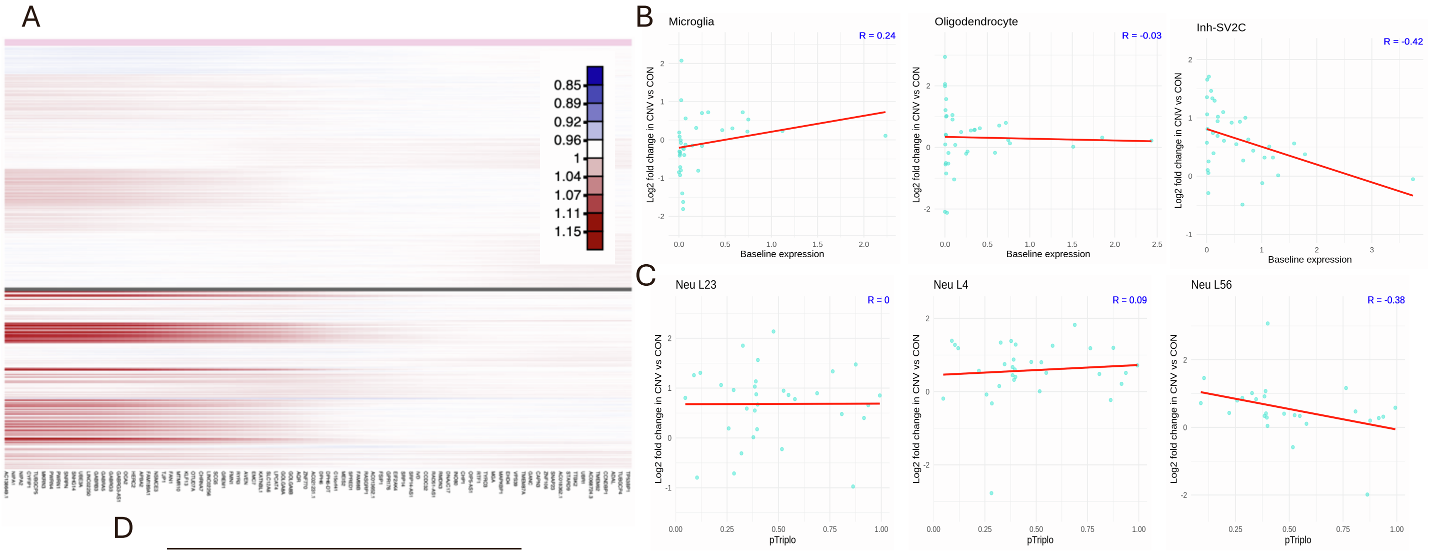

D

Gene	pTriplo
TUBGCP5	0.3846449
CYFIP1	0.87568458
NIPA1	0.52507227
NIPA2	0.39983122
MAGEL2	0.35396615
SNRPN	0.22130561
UBE3A	0.93655862
ATP10A	0.68789539
GABRB3	0.99263489
GABRA5	0.32659342
GABRG3	0.39635259
OCA2	0.08859845
HERC2	0.7635295
APBA2	0.38461863
TJP1	0.91646147
KLF13	0.47624642
OTUD7A	0.80825112
CHRNA7	0.57981061

Figure S5. Assessing mediating factors to dup15q expression changes. A. inferCNV reveals heterogeneous expression increases in region of duplication in dup15q cases (below grey line) across samples as compared to control (above grey line) within all nuclei (each row). Heatmap color scale indicates decreased (blue) or increased expression. **B.** Cell-type-specific examples of the association between baseline expression in controls and the fold change expression in dup15q. Within the duplicated region, genes that are highly expressed in different cell types demonstrate modest changes in expression in dup15q cases. p-values are non-significant except for in Inh-SV2C (p=.01, not adjusted for multiple comparisons) **C.** Association of pTriplo in cell-type-specific examples. All p-values > .050. **D.** pTriplo metric for select dup15q genes

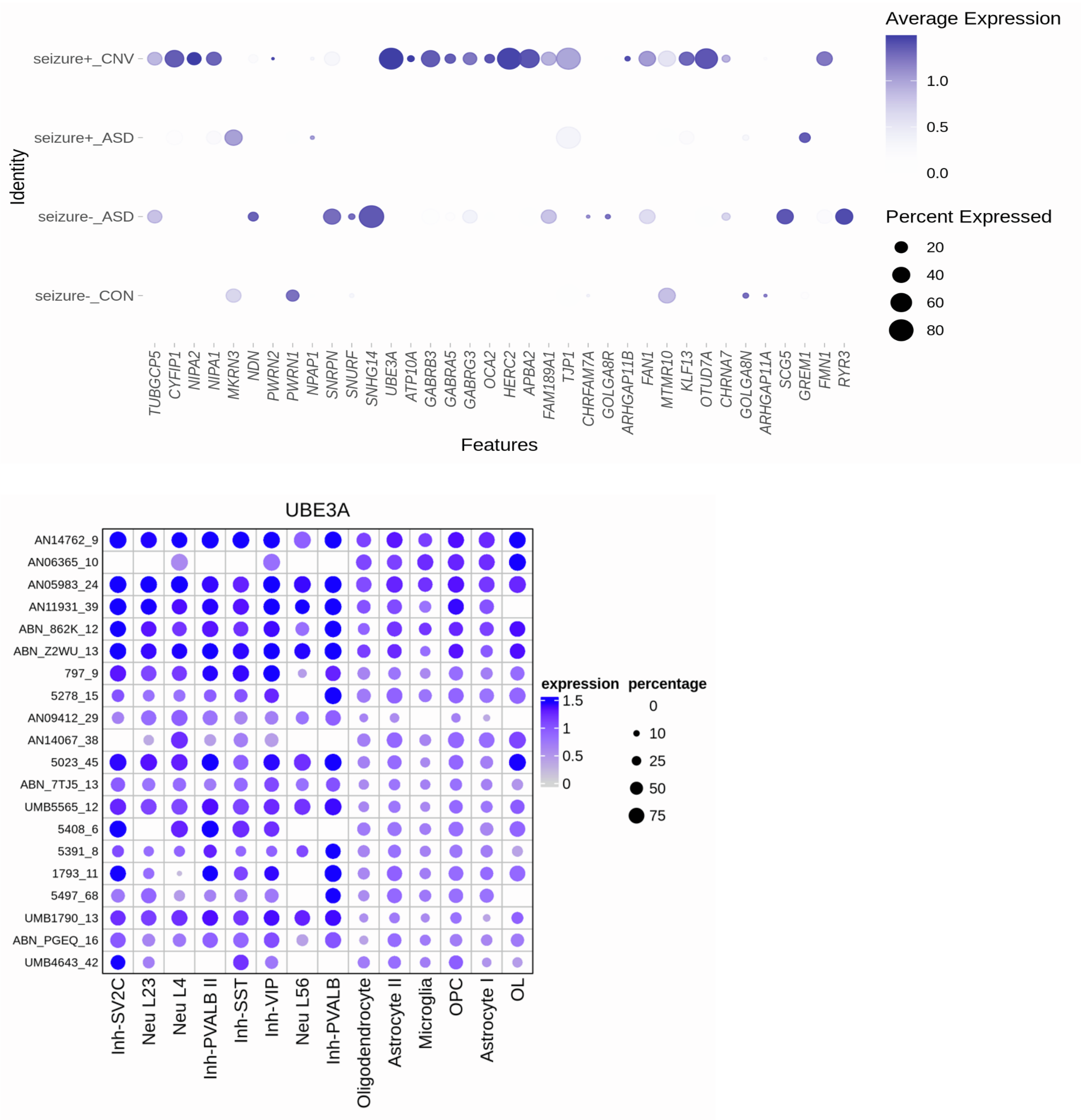

Figure S6: Impact of seizure diagnosis or other sources of individual variability on dup15q gene expression changes. Top: Presence of seizure diagnosis in ASD cases does not recapitulate dup15q gene expression changes observed within the duplicated (CNV) region as visualized with dot plot. Bottom: UBE3A expression is separated out by each individual sample (sample_age) and cell type to demonstrate that although there is notable heterogeneity between samples, effects are not being driven by an individual sample or factor such as age.

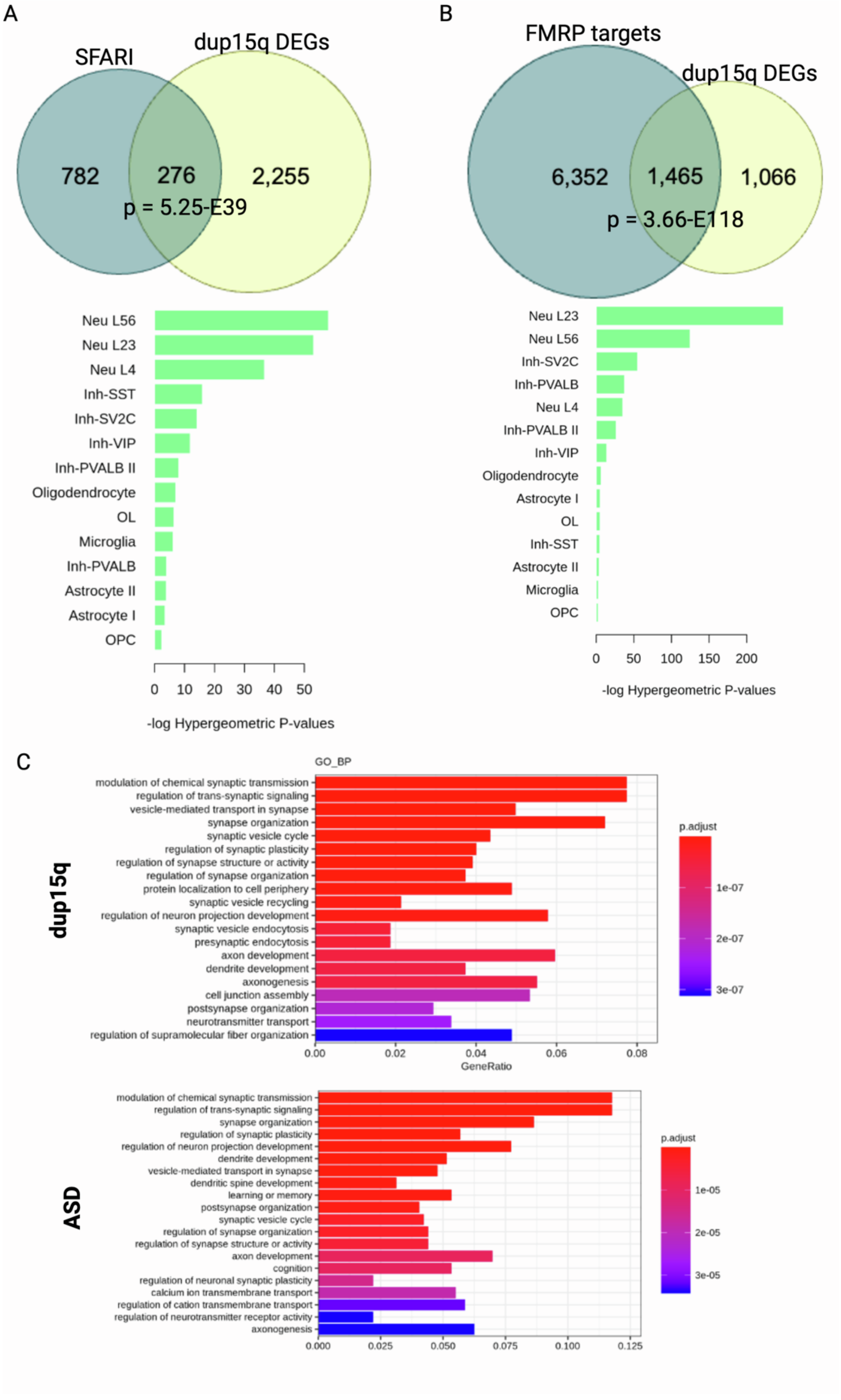

Figure S7: Neurobiological substrates in dup15q. Overlap of known (A) SFARI pathogenic ASD risk genes as well as (B) FMR1 protein target genes, demonstrates significant enrichment (p-value indicated in figure, hypergeometric test) These genes demonstrate notable enrichment in excitatory neuron subtypes. **C.** Using gene ontology analysis, we also found evidence for biological process terms involved in synaptic function. Presented are results from differential expression analysis in layer 2/3 neurons in each condition vs. control.

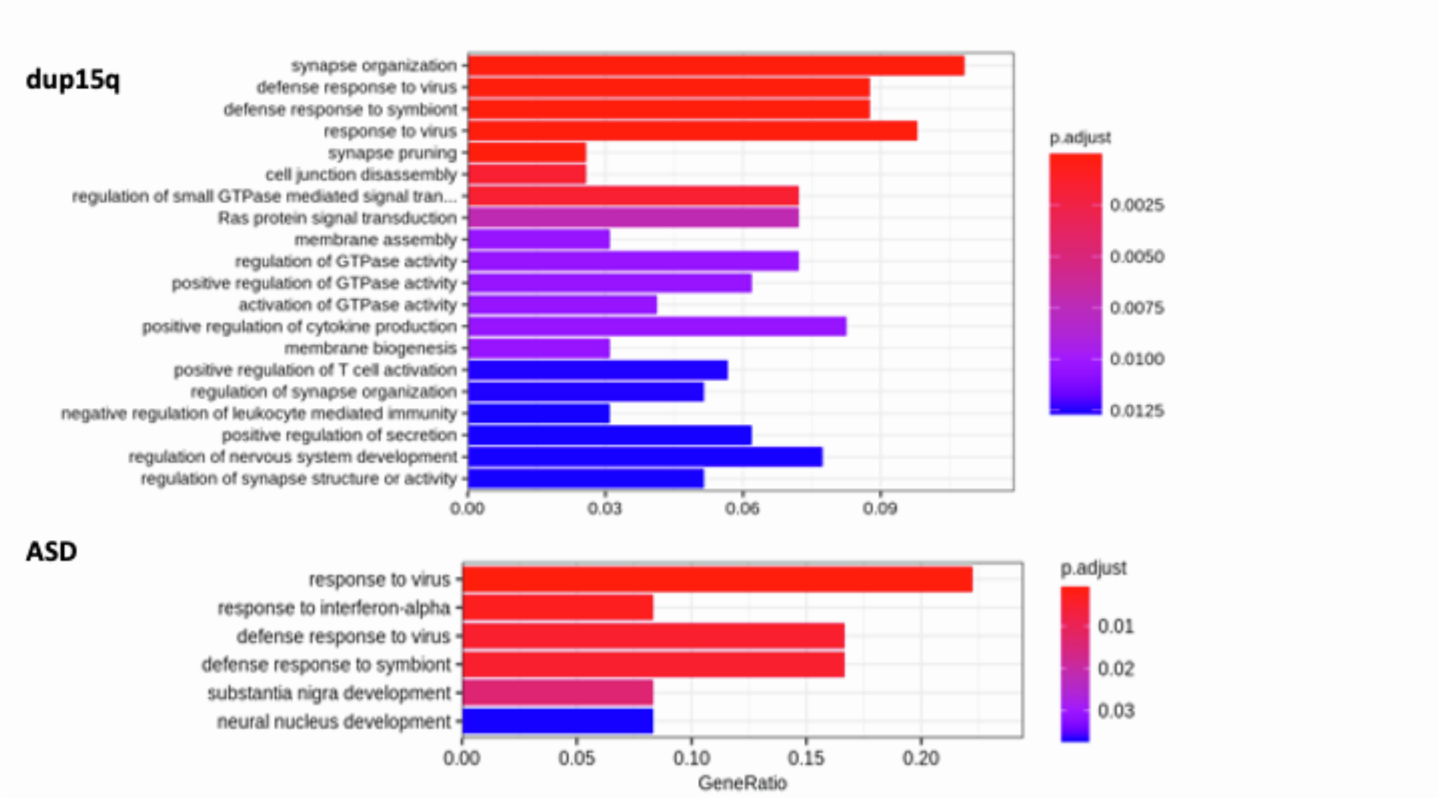

Figure S8: Microglia gene ontology analysis. Using gene ontology analysis, we found evidence suggesting dysregulation of synaptic pruning only in dup15q microglia, but not ASD (compared to control).

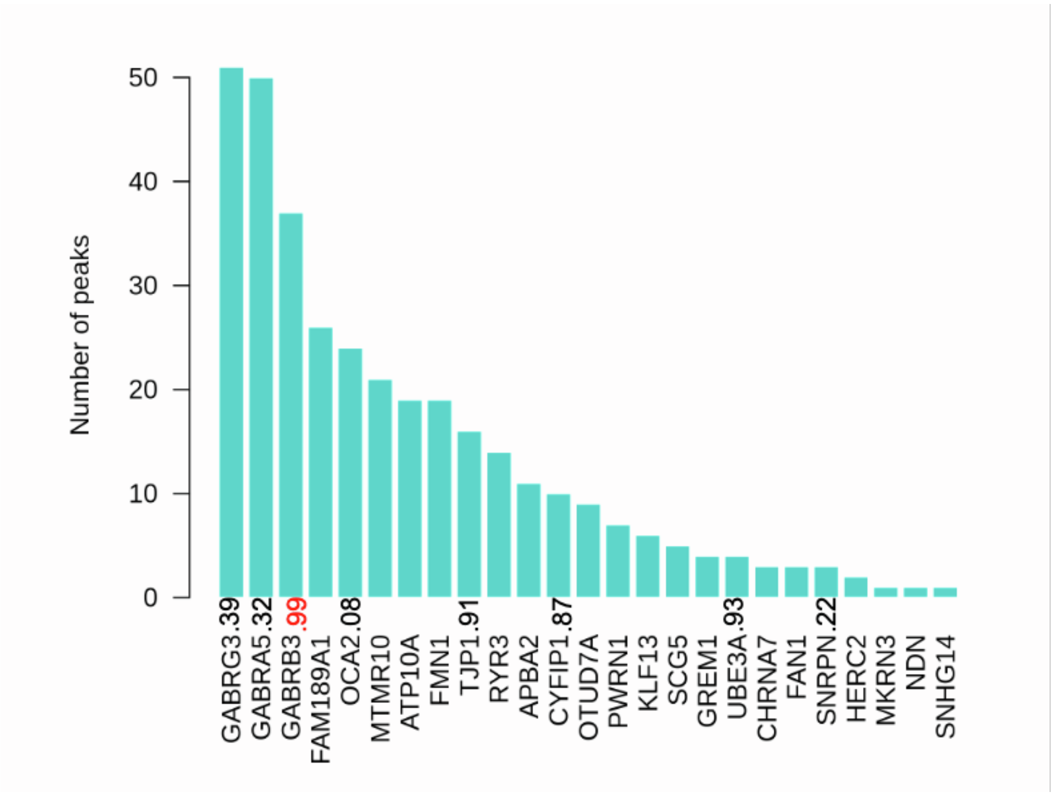

Figure S9: Dup15q gene-peak analysis. The LinkPeaks functionality in Signac was applied to the integrated multi-omic data-set, to identify significant peaks associated with genes within the duplicated region, which was calculated given GC content, accessibility, and length of the peak. The default thresholding was used which included p value < .05, Z-score < .05, and distance = 500,000 base pairs. There was variability in the number of peaks associated with genes within the duplication; there was not a clear correlation between number of peaks and pTriplo (Select pTriplo metrics shown above gene name for clarity, with significant pTriplo score in red.)

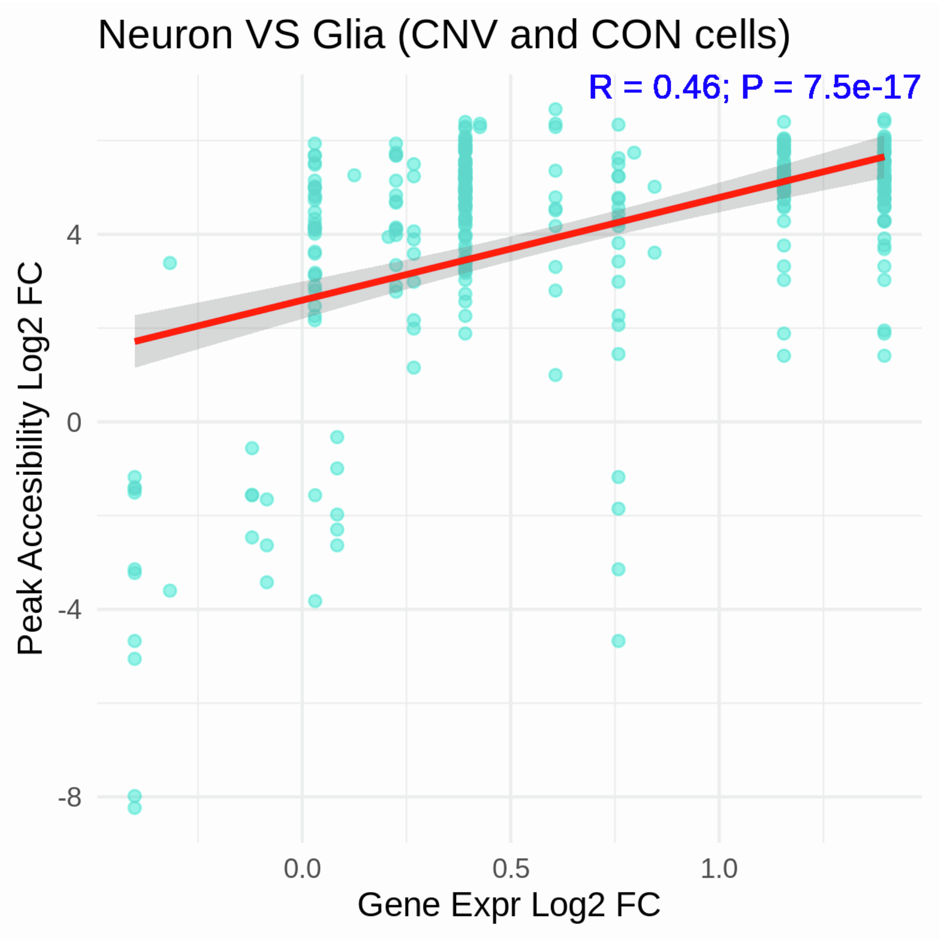

Figure S10: Association between differential chromatin accessibility and gene expression for peaks significantly associated with dup15q genes in the CNV vs Control comparison. Correlation analysis demonstrated a significant positive relationship (R=0.46, p < .001) between differential peak accessibility and gene expression for dup15q associated genes/peaks in neuron vs. glia comparison. After exclusion of ASD samples, the intersection of dup15q DEG lists between 1) CNV and control and 2) neurons and glia (both padj <.05 using FindMarkers function) was used to then create a list of linked peaks (with the LinkPeaks function) associated with dup15q region DEGs. Significantly differentially accessible peaks for the above DEG associated peaks were identified as above. Finally, the fold change expression in dup15q region DEGs was correlated with the differential accessibility of the associated peaks.

Supplemental Tables

Logfc = 0.2	# DEGs (all cells)	# DEGs (neuron cells)	# DEGs (glia cells)
CNV vs CON	126	623	206
ASD vs CON	34	329	45
CNV vs ASD	52	101	113

Table S1: **Differentially expressed genes.** Number of significant differentially expressed genes per comparison.

Cell Type	# DEGs	# Upreg	# Downreg	Cell Type	# DEGs	# Upreg	# Downreg
Non-neuronal				Neuronal			
Pericyte	48	23	25	Neu L56	3	2	1
Endothelial	11	7	4	Neu L4	308	239	69
Microglia	240	124	116	Neu L23	1,307	1,162	145
Astrocyte I	115	60	55	Inh-SV2C	315	290	25
Astrocyte II	124	82	42	Inh-VIP	107	84	23
Oligodendrocyte	119	78	41	Inh-PVALB	175	165	10
OL	5	3	2	Inh-SST	74	40	34
OPC	22	14	8	Inh-PVALB II	205	193	12
T Cells	1	1	0				

Table S2: **Cell-type-specific differentially expressed genes.** Number of significant differentially expressed genes in high resolution differential expression analysis, in dup15q vs control comparison (p-value adj<0.05).

Cell Ranger (RNA-seq)	Estimated Number of Cells	Mean Reads per Cell or ATAC Mean raw read pairs per cell	Median Genes per Cell or ATAC # peaks	Number of Reads or ATAC sequenced read pairs
AN14762	4,545	62,551	3,226	284,295,419
AN06365	3,823	79,570	3,669	304,195,930
AN05983	5,447	71,327	4,181	388,517,150
AN11931	4,336	67,944	5,156	294,603,243
797	6,304	63,125	3,064	397,937,622
5278	5,781	144,309	3,778	834,250,234
AN09412	1,458	176,120	3,969	256,783,196
AN14067	3,036	102,840	3,491	312,221,137
5023	4,285	65,205	3,529	279,402,186
5408	4,390	59,474	3,149	261,092,263
5391	11,489	29,686	1,052	341,060,553
1793	3,151	73,108	3,218	230,363,514
5497	5,557	72,214	3,029	401,292,261
**Arc (ATAC-seq)**				
ABN_862K	6019	41998.3567	107796	252,788,109
ABN_Z2WU	7276	38336.3718	184784	278,935,441
ABN_7TJ5	5867	40012.9637	195266	234,756,058
UMB5565	4378	59595.0763	155245	260,907,244
UMB1790	7189	34747.195	209764	249,797,585
ABN_PGEQ	4918	48866.7477	133719	240,326,665
UMB4643	8327	27741.7383	129083	231,005,455

Table S3: Technical sample information. Read coverage breakdown by sample for snRNA-seq and snATAC-seq components as indicated from Cell Ranger and Arc output.

Supplemental Methods

Optical Mapping of genomic DNA from post-mortem brain: 15-20 mg of brain tissue was homogenized for 10 seconds in Bionano Genomics Homogenization Buffer (P/N 20406) using a TissueRuptor, passed through a 40μm filter, then pelleted at 2000 x g for 5 minutes. The pellet was resuspended and re-pelleted from Bionano Genomics Wash Buffer A (P/N 20407). Homogenized tissue was then digested in 50μL of Protease solution and Bionano Genomics Lysis and Binding Buffer (LBB) (P/N 20375) on a HulaMixer for 15 minutes at 10 rpm. Tissue was further digested in Proteinase K on a HulaMixer for 15 minutes at 10 rpm. Enzymes were inhibited by PMSF.

gDNA binding was performed in the presence of Bionano Genomics Salting Buffer (P/N 20404) and 100% Isopropanol to a Bionano Genomics Nanobind Disk (P/N 20402) on a HulaMixer for 30 minutes at 10 rpm. Nanobinds were washed with Bionano Genomics Wash Buffer 1 (P/N 20376) and Wash Buffer 2 (P/N 20377) (2 rounds each) using a Dynamag Tube Rack. gDNA was eluted from Nanobind using Bionano Genomics Elution Buffer (P/N 20378). gDNA was homogenized for 1 hour at 15 rpm and incubated at room temperature for at least 3 days before labeling and staining. gDNA was fluorescently labeled and stained using the Bionano Genomics Direct Label and Stain Kit (P/N 80005) following Bionano Prep Direct Label and Stain protocol (P/N 30206).

Data was collected on the Saphyr instrument to a target throughput of 1500 Gbp or 400X effective coverage of the GRCh38 reference. Data was submitted to the Rare Variant Analysis pipeline on Bionano Solve version 3.6.1. The Rare Variant Pipeline aligns molecules to the reference to detect relative differences in the sample, forms consensus maps of molecule support in these loci and in the presence of sufficient molecule support, a structural variant call is made. The molecule data was subsequently down-sampled to 400Gbp or 100X coverage of the GRCh38 reference and submitted to the De Novo Assembly pipeline in Bionano Solve version 3.6.1 during which molecules were assembled de novo to form haplotype consensus maps, which were then aligned to the GRCh38 reference for structural variant detection and calling. In both scenarios, global reference coverage is used to determine copy number aberrations in addition to the aforementioned structural variant calling. Results were reviewed in Bionano Access 1.6.1. During review, structural variant and copy number calls were filtered against the Bionano provided mask files which remove structural variant and copy number calls in complex or poorly constructed regions of the GRCh38 reference. Variant calls were filtered against the Bionano control database of healthy individuals retaining only variants present in less than 1% of the control database. Variants were also filtered according to recommended confidence score filtering; 0 for indels, 0.7 for inversions, 0.3 for intrachromosomal fusions, 0.65 for intrachromosomal translocations, and no confidence scores were calculated for duplications where a placeholder value of -1 is used. Variants remaining after filtering were further curated by careful review of the data.
